# Fluorinated rhamnosides inhibit cellular fucosylation

**DOI:** 10.1038/s41467-021-27355-9

**Published:** 2021-12-02

**Authors:** Johan F. A. Pijnenborg, Emiel Rossing, Jona Merx, Marek J. Noga, Willem H. C. Titulaer, Nienke Eerden, Raisa Veizaj, Paul B. White, Dirk J. Lefeber, Thomas J. Boltje

**Affiliations:** 1 Department of Synthetic Organic Chemistry, Institute for Molecules and Materials, Radboud University, Heyendaalseweg 135, 6525AJ Nijmegen, The Netherlands; 2 Department of Laboratory Medicine, Translational Metabolic Laboratory, Radboud University Medical Center, Geert Grooteplein Zuid 10, 6525GA Nijmegen, The Netherlands; 3 Department of Neurology, Donders Institute for Brain, Cognition and Behavior, Radboud University Medical Center, Geert Grooteplein Zuid 10, 6525GA Nijmegen, The Netherlands

**Keywords:** Monosaccharides, Drug discovery and development, Cell biology

## Abstract

The sugar fucose is expressed on mammalian cell membranes as part of glycoconjugates and mediates essential physiological processes. The aberrant expression of fucosylated glycans has been linked to pathologies such as cancer, inflammation, infection, and genetic disorders. Tools to modulate fucose expression on living cells are needed to elucidate the biological role of fucose sugars and the development of potential therapeutics. Herein, we report a class of fucosylation inhibitors directly targeting de novo GDP-fucose biosynthesis via competitive GMDS inhibition. We demonstrate that cell permeable fluorinated rhamnose 1-phosphate derivatives (Fucotrim I & II) are metabolic prodrugs that are metabolized to their respective GDP-mannose derivatives and efficiently inhibit cellular fucosylation.

## Abstract (web summary)

Aberrant expression of fucosylated glycans has been linked to several disease states. Control of fucose expression on live cells is needed to aid research and therapy development. Here the authors report on the development of a class of fucosylation metabolic prodrug inhibitors and demonstrated inhibition of cellular fucosylation.

## Introduction

**Fig. 1:**
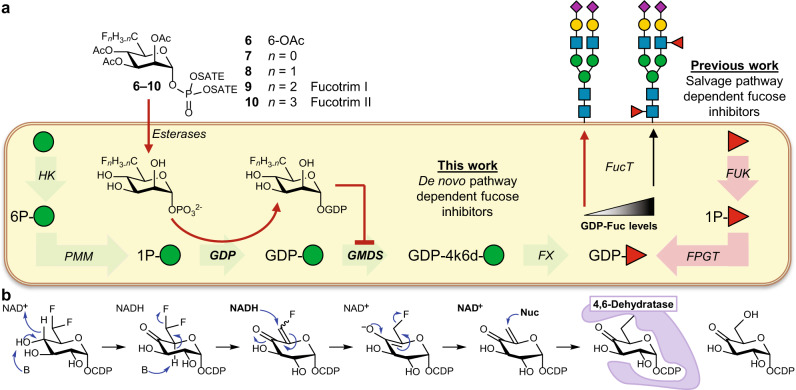
Schematic overview of inhibitor design. **a** Working model of metabolic fucosylation inhibitors. **b** Previous work showing the mechanism of action of different 4,6-dehydratase inhibitor acting on CDP-d-glucose 4,6-dehydratase. Abbreviations: HK hexokinase, PMM, phosphomannomutase, GDP, mannose-1-phosphate guanylyltransferase, GMDS GDP-mannose 4,6 dehydratase, FX GDP-4-keto-6-deoxy-d-mannose-3,5-epimerase-4-reductase (GMER), FUK fucokinase, FPGT fucose-1-phosphate guanylyltransferase, SATE *S*-acetyl-2-thioethyl.

l-Fucose (Fuc) is a 6-deoxyhexose expressed at the termini of glycan chains that decorate cell surface proteins and lipids^1^. The fucose residues on glycoconjugates are essential mediators of physiological processes. For example, the fucose moiety in the tetrasaccharide sialyl Lewis^x^ (sLe^x^) expressed on leukocytes is recognized by selectin receptors that regulate leukocyte recruitment and extravasation. The aberrant expression of sLe^x^ and increased fucosylation has been linked to pathologies, most notably cancer, and has been shown to promote tumor progression^2,3^. Reducing fucosylation of glycans in cancer using inhibitors of the fucose biosynthesis has therefore been recognized as a promising therapeutic option. Glycans or proteins are fucosylated by the action of thirteen fucosyltransferases (FucTs) that differ in acceptor glycan preference but all utilize GDP-fucose as the donor substrate^4^. Cells generate GDP-fucose either via the recycling of fucose released during glycan turnover in the lysosome (salvage pathway) or via de novo biosynthesis from mannose 1-phosphate (Fig. 1a).

To decrease cellular fucosylation, fucosyltransferase (FucT) inhibitors have attracted considerable attention^2^. A common strategy has been mimicking the natural substrate guanosine diphosphate fucose (GDP-Fuc). However, the essential GDP moiety in these compounds is associated with a high polarity and low stability thereby limiting their use in vivo by poor penetration of the cell membrane. This hurdle was recently overcome with the development of less polar, cell-permeable fucose derivatives which are metabolized to the corresponding active GDP-fucose analogs through the *salvage pathway*^5–9^. These compounds target FucTs by competitive inhibition and the de novo enzymes GMDS and FX by feedback inhibition. This strategy led to fucosylation inhibitors that are active in vitro and in vivo, showing promising anticancer effects in liver, breast and blood cancer models^10–14^. Moreover, combining a fucosylation inhibitor with immunotherapy vaccination in immunocompetent mice completely protected against tumor growth due to enhanced antibody-dependent cellular cytotoxicity (ADCC) with LS174T colorectal carcinoma and A20 lymphoma cells^12^. These inhibitors enter via the fucose salvage pathway yet it is estimated that ~90% of the GDP-Fuc pool is biosynthesized via de novo biosynthesis from GDP-mannose^15^. Thus, direct inhibition of de novo GDP-fucose biosynthesis could result in more potent inhibitors yet this approach remains unexplored so far to the best of our knowledge. GDP-fucose is biosynthesized from GDP-mannose by oxidation of the 4-carbon to a ketone and dehydration of the 6-position by GDP-mannose-4,6-dehydratase (GMDS, Supplementary Fig. 8a). Subsequent epimerization of the 3- and 5-position followed by the reduction of the 4-ketone by FX leads to GDP-Fucose. Hence, GMDS and FX are prime targets to develop de novo pathway inhibitors.

We were inspired by a mechanism-based inhibitor for CDP-d-glucose 4,6-dehydratase isolated from *Yersinia pseudotuberculosis*. Use of a 6-deoxy-difluoride modified CDP-glucose formed a reactive Michael acceptor inside the active site leading to entrapment (Fig. 1b)^16^. We hypothesized that GDP-d-6-deoxy-difluoro-mannose (GDP-d-Rha6F_2_) could inhibit GMDS, also a 4,6-dehydratase, through a similar mechanism. To allow passive diffusion over the cell membrane, lipophilic metabolic precursors were designed. Two entry points in the de novo biosynthesis were considered with precursors based on mannose (**1**–**5**) and mannose-1-phosphate (**6**–**10**). The lipophilic (thio)ester protecting groups are expected to be cleaved by esterases inside the cell, allowing the derivatives to enter the endogenous GDP-mannose biosynthetic pathway. To investigate the effect of degree of fluorination on the inhibitory potency we modified the 6-position with none to three fluorides (**2**–**5** & **7**–**10**, Fig. 1a)^17^.

In this work, we demonstrate that cell-permeable fluorinated rhamnose 1-phosphate derivatives (Fucotrim I & II) are metabolic prodrugs that are metabolized to their respective GDP-mannose derivatives and efficiently inhibit cellular fucosylation. We show that Fucotrim I is metabolized to its GDP-analog and targets de novo GDP-fucose biosynthesis via competitive GMDS inhibition.

## Results and discussion

**Fig. 2:**
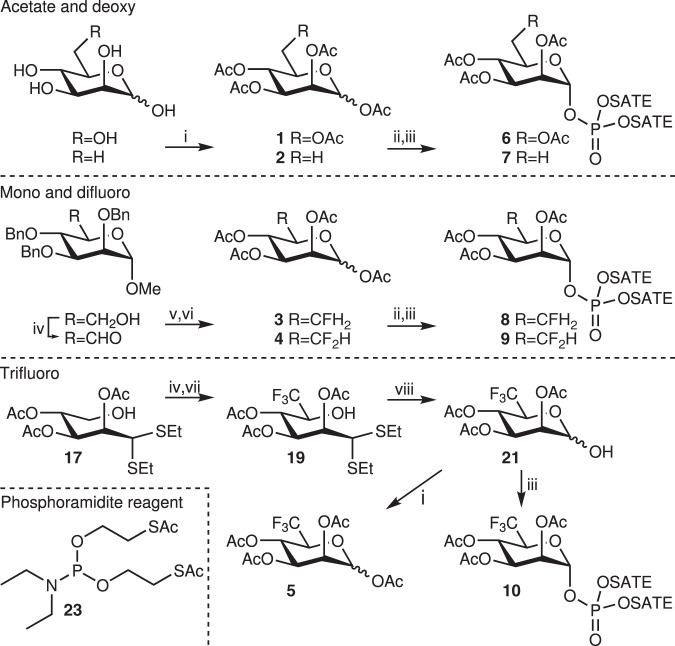
Synthesis of molecules **1**–**10**. i) Ac_2_O, Pyr; ii) H_2_NNH_2_·HOAc, DMF; iii) bis(S-acetyl-2-thioethyl)N,N-diethylphosphoramidite (**23**), 1*H*-tetrazole, ACN, then mCPBA; iv) DMP, NaHCO_3_, DCM; v) DAST, DCM; vi) Ac_2_O, H_2_SO_4_, AcOH; vii) TMSCF_3_, TBAF, THF, then AcOH; viii) NBS, H_2_O/acetone.

Compounds **1**–**10** were synthesized as described in Fig. 2. Elaborate synthetic procedures and analysis data of all compounds can be found in the Supplementary Methods. Compounds **1** and **2** were obtained by acetylation of d-mannose and d-rhamnose, respectively. Mono- and difluorides **3** and **4** were obtained via DAST mediated fluorination of the 6-OH or aldehyde, respectively, followed by acetolysis. The synthesis of protected 1-phosphate analogs **6**–**9** was achieved in a two-step sequence from their respective precursors **1**–**4**. Selective deprotection of the anomeric acetyl ester was achieved using hydrazinium acetate. The resulting lactol was reacted with phosphoramidite reagent **23** bearing two *S*-acetyl-2-thioethyl (SATE) groups and subsequently oxidized to corresponding phosphate triesters **6**–**9**. Trifluorides **5** and **10** were prepared via a homologation approach by introducing a CF_3_-group at the C-5 position of the pentose d-lyxose. To this end, derivative **17** was oxidized and reacted with a trifluoromethyl anion affording a separable mixture of the desired 6-trifluoro d-rhamnose (**19**) and the byproduct l-gulose (**20**) derivative. Hydrolysis of the thioacetal with NBS and water resulted in ring closure and the resulting lactol was acetylated or phosphorylated to afford **5** and **10**, respectively. In these synthetic routes, the 4-OH is always protected which ensures the formation of pyranoses, even when the 5-oxygen electrophilicity is decreased.

**Fig. 3:**
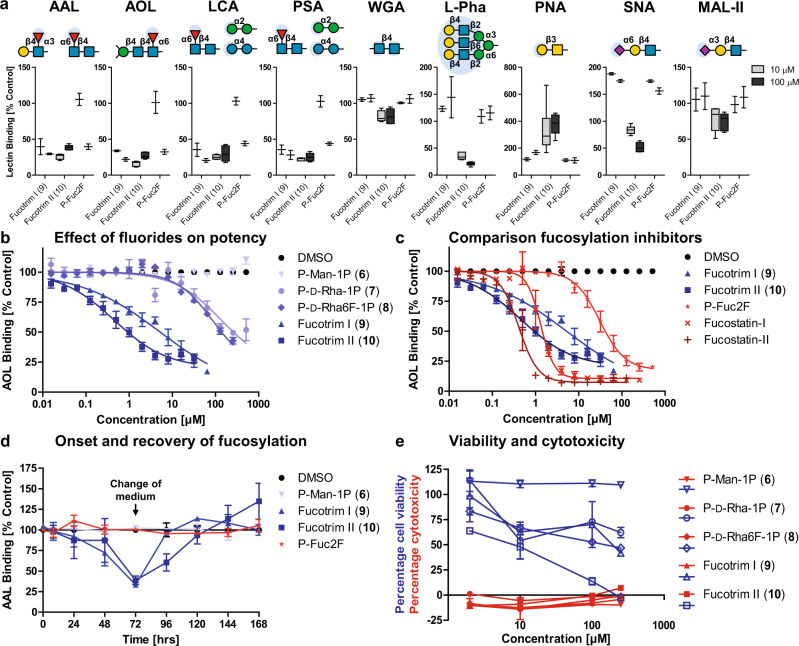
In vitro evaluation of fucosylation inhibitors. **a** Effect of 10 or 100 µM Fucotrim I (**9**) & II (**10**) and P-Fuc2F on cell surface glycosylation using different lectins, presented as percentage lectin binding compared to DMSO control in a Box and Whisker Plot with 5–95 percentiles (Supplementary Fig. 2). *N* ≥ 3 biologically independent experiments of 10.000 gated cells per sample and only *N* = 2 technical replicates per experiment for DMSO control. (**b**) Effect of C6-modifications on potency of inhibition presented as mean ± SEM. *N* ≥ 3 biologically independent experiments of 10.000 gated cells per sample and *N* = 2 technical replicates for each experiment. **c** Potency of Fucotrim I (**9**) and II (**10**) (blue) compared to known salvage pathway-dependent fucose inhibitors P-Fuc2F, Fucostatin-I and Fucostatin-II (red) using AOL lectin (*n* = 3) presented as mean ± SEM. *N* ≥ 3 biologically independent experiments of 10.000 gated cells per sample and *N* = 2 technical replicates for each experiment. **d** Onset and recovery of defucosylation. THP1 cells were incubated with 10 or 100 µM (Supplementary Fig. 3a, c) compound or DMSO control and fucosylation levels were determined with AAL lectins or AOL lectins (Supplementary Fig. 3b, c) for 6 days, presented as mean ± SEM. *N* ≥ 3 biologically independent experiments of 10.000 gated cells per sample and *N* = 2 technical replicates for each experiment. **e** The effect on viability (blue) and cytotoxicity (red) on THP1 cells after incubation with 2, 10, 100, or 250 µM **6**–**10** or **1**–**5** (Supplementary Fig. 4) for 3 days was determined with an XTT and LDH respectively and presented as percentage cell viability or cytotoxicity compared to DMSO control, presented as mean ± SEM. *N* = 1 biologically independent experiments of 10.000 gated cells per sample and *N* = 2 technical replicates.

The inhibitory potency of **1**–**10** was assessed at different concentrations in human THP-1 cells with fucose specific AAL and AOL lectins (Supplementary Fig. 1a–d). The EC_50_ values were determined, defined as the concentration where a 50% decrease in lectin binding compared to control was observed (Table 1). The non-phosphorylated compounds **1**–**5** did not show any inhibition of fucosylation. In contrast, from the phosphorylated set **6**–**10**, the mono-, di-, and trifluorinated derivatives **7**–**10** displayed inhibition of fucosylation. A trend was observed where increasing the number of C-6 fluorides increased its potency (Fig. 3b). Inhibitors P-d-Rha6F_2_-1P (**9**) and P-d-Rha6F_3_-1P (**10**) afforded (sub)micromolar inhibition of fucosylation and were dubbed Fucotrim I (**9**) and Fucotrim II (**10**), respectively. Both Fucotrim I (**9**) & II (**10**) were more potent than fucosylation inhibitor P-Fuc2F (1,3,4-tri-*O*-acetyl-2-deoxy-2-fluoro-l-fucose), a known salvage pathway-dependent inhibitor. Also in human Jurkat cells potent inhibition for Fucotrim I (**9**) and II (**10**) was observed (Table 1; Supplementary Fig. 1e, f). In mouse cell line EL4 however, only Fucotrim I (**9**) inhibited fucosylation while both Fucotrim II (**10**) and P-Fuc2F did not inhibit fucosylation (Table 1; Supplementary Fig. 1g, h).

**Table 1:** EC_50_ values in micromolar for fucose expression inhibition^[a]^.

	THP-1		Jurkat		EL4	
Compound	AAL	AOL	AAL	AOL	AAL	AOL
DMSO	NI	NI	NI	NI	NI	NI
P-d-Man-1P (**6**)	NI	NI	NI	NI	NI	NI
P-d-Rha-1P (**7**)	NI	137	NI	153	NI	NI
P-d-Rha6F-1P (**8**)	267	84	174	345	NI	NI
P-d-Rha6F_2_-1P (**9**)	2.0	5.3	5.9	4.1	14	38
P-d-Rha6F_3_-1P (**10**)	0.61	0.45	28	13	NI	NI
P-Fuc2F	45	31	115	121	NI	NI

[a] Three cell lines (THP-1, Jurkat, EL4) were cultured for 3 days with 0–512 µM of compound **6**–**10**, P-Fuc2F or DMSO control. The cells were stained with two fucose specific lectins (AAL & AOL) and analyzed by flow cytometry, presented as mean percentage lectin binding normalized to control. *N* ≥ 3 biologically independent experiments of 10.000 gated cells per sample and *N* = 2 technical replicates for each experiment. NI, no inhibition (EC_50_ > 500 µM).

Since Fucotrim I and II (**9**&**10**) are mannose-1-phosphate derivatives, it is possible that other glycosylation pathways such as mannosylation would be affected. Having established the ability to inhibit cellular fucosylation with AAL and AOL lectins, the effect on total cell surface glycosylation was evaluated using additional lectins after 3 days incubation (Fig. 3a; Supplementary Fig. 2). *N*-glycans consist of a biantennary mannose containing backbone, recognized by WGA, and can be branched, recognized by L-Pha. These branches can be terminated with galactose, recognized by PNA, or α2,6- and α2,3-linked sialic acids recognized by SNA and MAL-II, respectively^18,19^. LCA and PSA did show decreased binding for Fucotrim I (**9**), however, while they resemble the branching preference of WGA, their binding is restricted to *N*-glycans with α1,6-linked core fucosylation^18^. The decrease in binding for these lectins can thus be attributed to defucosylation as confirmed by a decrease in AAL and AOL binding and no effect on WGA binding. SNA binding is increased for both Fucotrim I (**9**) and P-Fuc2F. Fucotrim II (**10**) showed similar defucosylation as for Fucotrim I (**9**), however, it also decreased L-Pha and SNA binding and to a lesser extent WGA and MAL-II. Also an increase in PNA binding was observed, while this promiscuity was not observed for Fucotrim I (**9**) and known fucosylation inhibitor P-Fuc2F. To further evaluate the potency of Fucotrim I (**9**) and II (**10**), we made a direct comparison with known fucosylation inhibitors P-Fuc2F, P-l-Fuc6F_3_ (Fucostatin I) and P-l-Fuc-1CH_2_P (Fucostatin II) (Fig. 3c)^5–7^. The EC_50_ values for known Fucostatin I and II were determined at 1.3 and 0.41 µM, respectively, which is in the same range as Fucotrim I (**9**) and II (**10**) under these conditions (Table 1). P-Fuc2F was significantly less potent with an EC_50_ of 31 µM. The onset of inhibition and duration for recovery was evaluated on THP-1 cells with 10 and 100 µM compound using the AAL lectin (Fig. 3d; Supplementary Fig. 3a). In line with the potency data, no inhibition was observed for known inhibitor P-Fuc2F at 10 µM concentration. At the same concentration, Fucotrim I (**9**) and II (**10**) decreased lectin binding by over 50% after 3 days with full recovery to normal fucosylation levels after 4–5 days. At 100 µM concentration Fucotrim I (**9**) and II (**10**) inhibit fucosylation after 1 day and to over 50% after 3 days. Fucosylation was fully recovered to normal levels after 6 days. Similar results were obtained using the AOL lectin (Supplementary Fig. 3b, c). Finally, a toxicity profile of **1**–**10** was established by monitoring the metabolic activity and cell death of THP-1 cells after three days of treatment (Fig. 3e; Supplementary Fig. 4). Importantly, none of the compounds were cytotoxic at concentrations up to 250 μM. However, the cell viability was decreased which might be attributed to the known relation between fucosylation and proliferation^10,11,20^. The effect on cell viability of Fucotrim I and II becomes especially important in the investigation of its therapeutic potential, targeting cancer cells. It has been suggested that the reduced proliferation after incubation with known fucosylation inhibitors is caused by acting on the de novo biosynthesis of GDP-fucose rather than acting on fucosyltransferases, which correlates with our findings^21^.

**Fig. 4:**
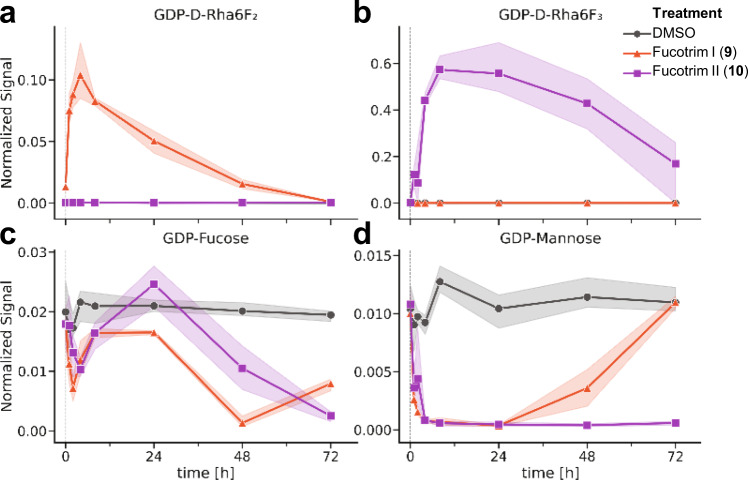
Nucleotide sugar analysis. THP-1 cells were incubated for indicated time points with 10 µM Fucotrim I (**9**) or Fucotrim II (**10**) or with DMSO control. After sample preparation, the GDP-Rha6F_2_ (**a**), GDP-Rha6F_3_ (**b**), GDP-Fuc (**c**), and GDP-Man (**d**) levels were analyzed using reverse-phase ion-pairing chromatography coupled to a triple quadrupole mass spectrometer operating in negative ion mode and presented as their mean abundance in the nucleotide sugar pool (line) with 95% confidence interval (shading) (Supplementary Fig. 5). *N* = 3 biologically independent experiments and *N* = 1 technical replicates.

To test our hypothesized prodrug strategy, we studied the metabolism of Fucotrim I (**9**) and II (**10**) to their corresponding GDP-analogs inside the cell. To this end, intracellular nucleotide sugar levels were analyzed. THP-1 cells were treated for different time points with Fucotrim I (**9**) and II (**10**) and extracted metabolites analyzed using reverse-phase ion-pairing chromatography coupled to a triple quadrupole mass spectrometer operating in negative ion mode (Fig. 4; Supplementary Fig. 5). For analysis of GDP-d-Rha6F_2_ and GDP-d-Rha6F_3_ analogs, theoretical mass transitions were programmed. After incubations for only 1 h, both Fucotrim GDP-analogs were observed with a maximum abundance after 4–8 h (Fig. 4a). Subsequently, GDP-fucose levels decreased after 1–2 days for both inhibitors, indicating inhibition of GDP-fucose biosynthesis by Fucotrim I (**9**) and II (**10**) (Fig. 4b). The time lag observed for the appearance of the Fucotrim GDP-analogs (4–8 h) and lowering of the GDP-fucose levels (1–2 days) might be explained by initial supplementation of the GDP-fucose levels via the salvage pathway in the intermediate period. GDP-mannose levels were also affected by both inhibitors. This may be explained by the large pulse of intracellular d-Rha6F_2_-1-phosphate and d-Rha6F_3_-1-phosphate which are competing with mannose-1-phosphate for conversion by GDP to their respective GDP-analogs thereby lowering GDP-mannose levels. For Fucotrim I (**9**), the GDP-mannose levels recovered after 3 days and for Fucotrim II (**10**), GDP-mannose levels did not recover within the 3 days of the experiment. This correlates well with the lectin panel (Fig. 3a) where after 3 days incubation Fucotrim I (**9**) only shows an effect on fucosylation and Fucotrim II (**10**) shows an effect on multiple forms of glycosylation. Importantly, for both Fucotrim I (**9**) and II (**10**) no changes were observed for other nucleotide sugar levels such as UDP-glucose and UDP-galactose (Supplementary Fig. 5). From these results, it is clear that Fucotrim I (**9**) is a more specific inhibitor of fucosylation.

**Fig. 5:**
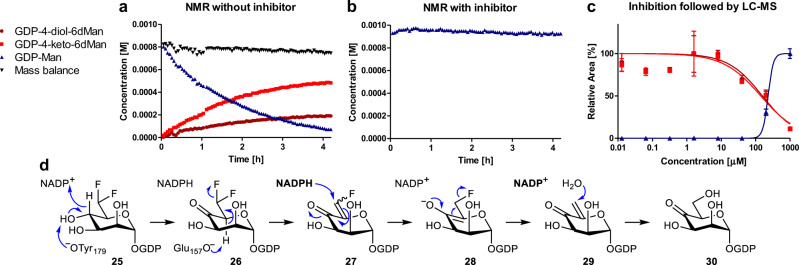
Enzymatic assays of the consumption of GDP-mannose by GMDS. GMDS (21 μM) was incubated with either (**a**) GDP-mannose (1 mM) or (**b**) preincubated with GDP-d-Rha6F_2_ (**25**) (0.5 mM) for 10 min before addition of GDP-mannose (1 mM) and reactions were followed by ^1^H-NMR for 4 h. Concentrations were determined relative to an internal standard and plotted over time. *N* = 1 experiment. (**c**) GMDS (21 μM) and GDP-mannose (1 mM) were incubated with 12.5 nM–1 mM GDP-d-Rha6F_2_ (**25**) for 1 h at 30 °C. Samples were quenched with 8 M urea and GDP-containing intermediates were analyzed with LCMS. Substrate and products were normalized and plotted against the concentration of inhibitor and presented as mean ± SEM. *N* = 2 independent experiments. (**d**) proposed mechanism of consumption of GDP-d-Rha6F_2_ by GMDS.

Fucotrim I (**9**) was designed to act as a mechanism-based inhibitor of GMDS as it has been shown that CDP-6-difluoromethylglucose acts as a mechanism-based inhibitor of the CDP-glucose 4,6-dehydratase (E_od_) enzyme. To investigate whether Fucotrim I (**9**) is indeed a GMDS inhibitor and establish its mechanism of inhibition, we recombinantly expressed GMDS. In addition, we prepared GDP-d-Rha6F_2_ (**25**) from **9** by deacetylation to afford the intermediate d-Rha6F_2_-1-phosphate followed by reaction with imidazole-GMP^22^. We validated the ability of recombinant GMDS (21 µM) to convert GDP-mannose to GDP-6-deoxy-4-keto-mannose using NMR (Fig. 5a; Supplementary Fig. 6)^23^. GDP-mannose (1 mM) was converted to GDP-6-deoxy-4-keto-mannose and its corresponding hydrate within ~4 h which is in line with an earlier report^23^. We repeated this experiment in the presence of 0.5 mM of GDP-d-Rha6F_2_ (**25**) which completely abolished the conversion of GDP-mannose thereby confirming that GDP-d-Rha6F_2_ is an inhibitor of GMDS (Fig. 5b; Supplementary Fig. 7). To establish the IC_50_ of GDP-d-Rha6F_2_, GMDS (21 µM) was incubated for 1 hr with 12.8 nM – 1 mM GDP-d-Rha6F_2_ and 1 mM of GDP-Man. The concentration of GDP-Man and both products, GDP-6-deoxy-4-keto-mannose and the corresponding hydrate were followed by LC-MS. Peak areas were normalized and plotted against the concentration of GDP-d-Rha6F_2_ (Fig. 5c) to obtain an IC_50_ value of 231 µM under these conditions.

Next, we investigated the ability of GDP-d-Rha6F_2_ to act as a mechanism-based inhibitor as previously described for a bacterial CDP-glucose 4,6-dehydratase (Fig. 1b)^16^. Molecular modeling of GDP-d-Rha6F_2_ and its putative metabolites in silico was performed by docking the natural substrate GDP-mannose at the 4-fluoro-GDP-mannose (G4F) binding site in a human GMDS crystal structure (6GPJ) in which cofactor NADP^+^ was co-crystallized (Supplementary Fig. 8)^23^. Subsequent docking of GDP-d-Rha6F_2_ and its putative metabolites resulting from GMDS mediated defluorination afforded binding poses with catalytic residues positioned such that oxidation, enolization, elimination and reduction do not seem to be hindered sterically. For the endogenous substrate, enolization would yield the final product. For the difluoride derivative, however, a second elimination might occur, affording electrophilic intermediate **29** that cannot be reduced due to the lack of reductant NADPH in the active site. In case of E_od_, nucleophilic addition of a sidechain residue to this intermediate afforded a covalent adduct^23^. To investigate the possibility of covalent adduct formation in our case we incubated GMDS with GDP-d-Rha6F_2_ and performed a tryptic digest followed by LCMS analysis. The active site fragment containing putative nucleophile Ser^156^ was retrieved but no covalent adduct could be detected (Supplementary Figs. 9–11). To investigate if GDP-d-Rha6F_2_ can be processed by GMDS we analyzed its nucleotide sugar metabolites using the aforementioned reverse-phase ion-pairing LCMS methodology. Upon incubation of GDP-d-Rha6F_2_ (100 µM) with an equimolar concentration of GMDS, metabolites at retention time 5.4 min were observed after incubation for 21 h (Supplementary Fig. 12). The m/z values of these metabolites corresponded to intermediate **27**, GDP-4-keto-mannose and their corresponding hydrates but due to their isobaric nature a definitive assignment could not be made. However, the formation of metabolites indicate that GDP-d-Rha6F_2_ can bind the active site of GMDS. Possibly, GDP-d-Rha6F_2_ can also bind the allosteric site of GMDS as demonstrated for GDP-l-Fuc6F_3_ although this would not be expected as the former is a substrate analogue and the latter a product analogue^7^. As GDP-d-Rha6F_2_ is metabolized in only minor quantities by GMDS, we conclude Fucotrim I is metabolized to GDP-d-Rha6F_2_ in cells, acting as a competitive inhibitor of GMDS and thereby decreasing the GDP-fucose pool.

In conclusion, we have developed in vitro validated de novo pathway-dependent fucosylation inhibitors. Fucotrim I (P-d-Rha6F_2_-1P, **9**) and Fucotrim II (P-d-Rha6F_3_-1P, **10**) are tools to study fucosylation function and are good candidates for further therapeutic development. Additionally, this approach could be considered for targeting other 4,6-dehydratases affording cell-active inhibitors.

## Methods

### Reagents

Biotinylated AAL, SNA, MAL-II, WGA, LCA, PSA, PNA, PHA-L, and GSL-1 lectins and 10x Carbo-free Blocking Buffer were purchased from Vector laboratories Inc. Biotinylated AOL lectin was purchased from TCI Europe. 0.2 mg/mL Streptavidin-phycoerythrin conjugate was purchased from Fischer Scientific (Invitrogen, eBioscience). Unnatural sugar derivatives were synthesized as described under synthetic procedures and stored at −20 °C at a concentration of 100 mM in DMSO.

### Cell culture

THP-1 cells (TIB-202, ATCC) and Jurkat cells (TIB-152, ATCC) were cultured in RPMI-1640 medium containing 2 mM Glutamine and 25 mM HEPES (Gibco^TM^, Life Technologies), supplemented with 10% v/v heat-inactivated fetal bovine serum (FBS) (Gibco^TM^, Life Technologies) and 1X antibiotic-antimycotic solution (100 units/mL of penicillin, 100 μg/mL of streptomycin, and 0.25 μg/mL Fungizone) (Gibco^TM^, Life Technologies) and passaged every 3-4 day by seeding a fraction of 0.5–2 mln cells of the culture per 10 mL medium. EL-4 cells (TIB-39, ATCC) were cultured in DMEM medium (Gibco^TM^, Life Technologies), supplemented with 10% v/v heat-inactivated fetal bovine serum (FBS) (Gibco^TM^, Life Technologies), 1X antibiotic-antimycotic solution (100 units/mL of penicillin, 100 μg/mL of streptomycin, and 0.25 μg/mL Fungizone) (Gibco^TM^, Life Technologies) and 2 mM Glutamine (Gibco^TM^, Life Technologies) and passaged every 2–4 days by seeding a fraction of 0.5–1 mln cells of the culture per 10 mL medium.

All cells were cultured at 37 °C, 5% CO_2_ in a humidified incubator.

### Lectin staining of cells

Protocol below was repeated until *n* > =3 for each compounds using cells at different passage numbers.

Cells were cultured in medium containing different concentrations of unnatural sugar derivatives on 96-wells plates (suspension cell lines; 13,000–20,000 cells per well) (Thermo Scientific). DMSO, at the same dilution as the unnatural sugar derivative stock solutions, was used as a positive control for lectin staining; P-Fuc2F was used for all concentrations as the negative control. Cells were incubated for three days at 37 °C and 5% CO_2_ in a humidified incubator. Cells were harvested and washed with 100 μl 1X CF-blocking buffer (Vector Laboratories Inc.) containing 1 mM CaCl_2_ and 1 mM MgCl_2_. The cells were then resuspended in 50 μl 0.5 μg/mL of 0.5 ng/mL biotinylated lectin in 1X carbo-free blocking buffer and incubated at 4–8 °C for 45–60 min. Cells were washed with 3 × 100 μl PBA (PBS containing 1% v/v FBS and 0.1% w/w sodium azide), incubated with 40 μl 1 μg/mL Streptavidin-phycoerythrin conjugate (Invitrogen, eBioscience) in PBA for 10–15 min at 4–8 °C. Cells were then washed again with 3 × 100 μl PBA, resuspended in PBA and fluorescence was measured with a flow cytometer (Beckman & Dickinson FACS-Calibur). Each replicate for each condition with >10,000 gated events. Data was processed using FlowJo V10 (FlowJo LLC). The percentage of lectin binding was obtained by normalizing the MFI values to the MFI values of the respective DMSO control.

### Lectin specificity assay

Protocol below was repeated until *n* > = 3 for each compounds using cells at different passage numbers. The culture medium was prepared in 96-wells plates (Thermo Scientific). For every unnatural sugar derivative, 11 wells containing 100 μM of unnatural sugar derivatives and 11 wells containing 10 μM of the unnatural sugar derivatives were prepared. DMSO, at same dilution as the tested probes, was used as a positive control for lectin staining. THP-1 Cells were cultured in the plates (20,000 cells per well) and the cells were incubated for three days at 37 °C and 5% CO_2_. Cells were harvested and washed with 100 μl 1X CF-blocking buffer (Vector Laboratories Inc.) containing 1 mM CaCl_2_ and 1 mM MgCl_2_. The cells were then resuspended in 50 μl 0.5 μg/mL of either 0.5 ng/mL biotinylated AAL, AOL, SNA, MAL-II, WGA, LCA, PSA, PNA, PHA-L or GSL-1 lectin in 1X carbo-free blocking buffer or with 50 μl of nonsupplemented 1X carbo-free blocking buffer and incubated at 4–8 °C for 45–60 min. Cells were washed with 3 × 100 μl PBA (PBS containing 1% v/v FBS and 0.1% w/w sodium azide), incubated with 40 μl 1 μg/mL Streptavidin-phycoerythrin conjugate (Invitrogen, eBioscience) in PBA for 10–15 min at 4–8 °C. Cells were then washed again with 3 × 100 μl PBA, resuspended in PBA and fluorescence was measured with a flow cytometer (Beckman & Dickinson FACS-Calibur). Each replicate for each condition with >10,000 gated events. Data was processed using FlowJo V10 (FlowJo LLC). The percentage of lectin binding was obtained by normalizing the MFI values to the MFI values of the respective DMSO control.

### Onset and recovery of inhibition

THP-1 cells were cultured in medium containing different concentrations of unnatural sugar derivatives on 96-wells plates (suspension cell lines; 13,000-20,000 cells per well) (Thermo Scientific). DMSO, at same dilution as the unnatural sugar derivative stock solutions, was used as a positive control for lectin staining; P-Fuc2F was used for all concentrations as negative control. Cells were incubated for eight days at 37 °C and 5% CO_2_ in a humidified incubator. The medium was changed after three and six days of incubation with cell culture medium without unnatural sugar derivatives. Cells were passaged 1:2 after 4, 6 and 8 days of incubation. Every day cells were harvested and washed with 100 μl 1X CF-blocking buffer (Vector Laboratories Inc.) containing 1 mM CaCl_2_ and 1 mM MgCl_2_. The cells were then resuspended in 50 μl 0.5 μg/mL of either 0.5 ng/mL biotinylated AAL, AOL, SNA, MAL-II, WGA, LCA, PSA, PNA, PHA-L or GSL-1 lectin in 1X carbo-free blocking buffer or with 50 μl of non-supplemented 1X carbo-free blocking buffer and incubated at 4–8 °C for 45-60 min. Cells were washed with 3×100 μl PBA (PBS containing 1% v/v FBS and 0.1% w/w sodium azide), incubated with 40 μl 1 μg/mL Streptavidin-phycoerythrin conjugate (Invitrogen, eBioscience) in PBA for 10-15 min at 4-8 °C. Cells were then washed again with 3 × 100 μl PBA, resuspended in PBA and fluorescence was measured with a flow cytometer (Beckman & Dickinson FACS-Calibur). Each replicate for each condition with >10,000 gated events. Data were processed using FlowJo V10 (FlowJo LLC). Percentage of lectin binding was obtained by normalizing the MFI values to the MFI values of the respective DMSO control.

### Viability and toxicity assays

Assays were performed using an XTT Cell Viability Assay kit (Invitrogen, CyQUANT) or an LDH-toxicity assay kit (Thermo Scientific) respectively. Assays were performed according to enclosed manuals. THP-1 cells were treated for 3 days with unnatural sugar analogs at 37 °C, 5% CO_2_ in a humidified incubator before adding the XTT/LDH reagents. For the control, cells were treated with DMSO. %Viability and %Cytotoxicity were calculated relative to the DMSO control for XTT and lysed untreated cells for the LDH assay.

### Nucleotide sugar analysis

THP-1 cells were incubated for 0, 1, 2, 4, 8, 24, 48, or 72 h with 10 μM Fucotrim I, Fucotrim II or DMSO control in triplo. For the 0, 1, 2, 4, 8 h timepoints, 3 mln cells were plated in 6-well plates, for the 24 h timepoint, 1.5 mln cells were plated per well, for the 48 h timepoint, 1 million cells were plated and for the 72 h timepoint, 750,000 cells were plated per well to ensure a total of 3 to 3.5 million cells at time of harvesting. At each timepoint, cells were harvested, medium was removed and the cells were washed twice with 2 mL 75 mM ammonium carbonate pH 7.4 buffer in MilliQ water. After removing the washing buffer, cells were snap-frozen in liquid nitrogen. Samples then were stored at −80 °C until workup. Samples were thawed on −20 °C ice-salt and the pellets were resuspended in 1 mL of extraction buffer (1:2:2 MilliQ water: acetonitrile: methanol v-v:v). The samples were incubated for 5 min at −20 °C. Samples were then spun down at 11696 × g in a tabletop centrifuge (Hettich MIKRO 20) and supernatant was transferred to 1.5 mL eppendorf tubes and solvent was removed with a speedvac (Savant SC100, RVT 100 with an external oil pump). Upon redissolving in 100 μL of MilliQ water, 10 μL was injected in to a Agilent 1290 LC-MS system using a modified version of previously published method for analysis of nucleotide sugars^24^. Agilent 6490 A QQQ mass spectrometer was operated in dynamic multiple-reaction monitoring MRM mode using in silico generated transitions. These operations were performed by the use of a script written in Python (v 3.9.2), an RDkit library (2020.09.5), and chemical structures of the target compound as the input (SMILES-codes). LC-MS data files were processed in Skyline version 20.2 using a target list chosen on the basis of the aforementioned *in silico*-generated transition list^25^. Resulting peak areas were normalized to the sum of all nucleotide sugars detected, excluding metabolites in pathways affected by treatment (all metabolites bearing a GDP-nucleotide) to obtain relative abundances. Plots of the data were generated in Python (v 3.8.5) using Pandas (v 1.3.2) and Seaborn (v 0.11.1) libraries.

### GMDS protein expression

Human GMDS was expressed as described in literature^23^. E. Coli BL21 competent cells were transfected with commercially available plasmid (Addgene plasmid # 39152) containing the gene for hGMDS and a His-tag. Expression was induced in sterilized 2x1L LB medium (1% w/v sodium chloride, 1% w/v tryptone, and 0.5% w/v yeast extract (Sigma Aldrich), containing 50 μg/ml Ampicillin) at OD_600_ ~ 2 by addition of 0.1 mM isopropyl-β-d-1-thio-galactopyranoside (IPTG) for 16 h at 16 °C. Cells were harvested by centrifugation for 45 min at 4 °C, 1993 × *g.* Pellet was taken up in buffer A (500 mM Sodium Chloride, 10 mM HEPES, 10% v/v Glycerol, pH 7.5) and cells were lysed by sonication for 10 min. Supernatant was recovered by centrifugation for 45 min at 4 °C, 1993 × *g*. Supernatant was loaded onto a 5 mL His-Trap FF column from (GE Healthcare, Little S7 Chalfont, UK) on a peristaltic pump, operated at ~3 mL/min. Columns were equilibrated for 20 min with buffer A. Column was washed with 15 column volumes of buffer B (500 mM NaCl, 10 mM HEPES, 30 mM imidazole, 0.1 mM TCEP, pH 7.5). GMDS was eluted using five column volumes of buffer C (500 mM NaCl, 10 mM HEPES, 300 mM imidazole, 0.1 mM TCEP, pH 7.5). Fractions containing the target protein were pooled and concentrated with Amicon Ultra-15 Centrifugal Filter Units (Merck Millipore, Billerica, MA, USA) to a concentration of ~185 μM, based on nanodrop and stored at -80 °C in aliquots of 55 μl. Total yield of GMDS was ~55 mg, based on nanodrop concentration.

### GMDS enzymatic assay using NMR

Consumption of GDP-mannose by GMDS with or without GDP-d-Rha6F_2_ (**25**) was monitored by NMR. The conversion and inhibition of GDP-Man to GDP-4′′-keto-6′′-deoxy-mannose and its corresponding hydrate was followed in time by ^1^H NMR based on a procedure from Pfeiffer et al^23^. The enzymatic reactions were performed in 50 mM potassium phosphate buffer (pD 7.5) at 25 °C. all nonenzymatic reagents were dissolved in D_2_O right before use. The buffer of the enzyme was exchanged by three successive washes with a tenfold excess of potassium phosphate buffer (50 mM; pD 7.5) using a Vivaspin 6 centrifugal concentrator (13000 x *g*, 10 Kd MWCO PES). The reactions were carried out with a concentration of 1 mM GDP-Man and 100 µM DSS (3-(trimethylsilyl)propionic-2,2,3,3-d_4_ acid sodium salt) as internal standard. Upon addition of hGDMS (21 µM, final volume 600 µL) the reaction was followed in time for 4 h. For the inhibition assay, GMDS was preincubated with GDP-d-Rha6F_2_ (**25**) (1 mM) for 15 mins before addition of the substrate (1 mM GDP-Man), the reaction was followed for 4 h after addition of GDP-Man. Spectra were recorded on a Bruker Avance III 500 MHz spectrometer. A ^1^H-NMR spectrum was recorded every 187 seconds after addition of substrate. Concentrations were calculated relative to the internal standard, using the integrals of the ^1^H-resonances at 5.51 ppm (GDP-4-keto-6-deoxy-Man), 5.44 ppm (GDP-Man), and 5.38 ppm (GDP-4-diol-6-deoxy-Man). Mass balance was calculated by comparing the sum of these integrals with the integral of the internal standard.

### GMDS enzymatic assay using LC-MS

IC_50_ determination by LC-MS: GMDS (21 μM) was incubated with GDP-mannose (1 mM) and varying concentrations of GDP-d-Rha6F_2_ (**25**) (12.8 nM–1 mM) in 50 mM phosphate buffer (pH 7.5) for 1 h. 2 μl of the reaction mixture was quenched by diluting with 8 M urea to a volume of 4 μl, snap-frozen in LN2 and stored at −80 °C. Samples were defrosted on ice and diluted 10X with MilliQ water before acquisition. Time course of enzymatic conversion by LC-MS: GMDS (21 μM) was incubated with either GDP-mannose (1 mM) or GDP-d-Rha6F_2_ (**25**) (1 mM) or both in 50 mM phosphate buffer (pH 7.5) for 4 h. At the different time points (0, 15, 60, 90, 120, 150, 180, 210 and 240 min), 2 μl of the reaction mixture was quenched by diluting with 8 M urea to a volume of 4 μl, snap-frozen in LN2 and stored at -80 °C. Samples were defrosted on ice and diluted 10X with MilliQ water before acquisition. Detection of possible metabolites of GDP-d-Rha6F_2_ **(25)** with LC-MS: GMDS (100 μM) was incubated either with or without GDP-d-Rha6F_2_ (**25**) (100 μM) in 50 mM phosphate buffer (pH 7.5) for 24 h. At varying time points (1, 15, 30, 60, 195 min and overnight at 1255 min), 10 μl of the reaction mixture was quenched by diluting with 10 μl 8 M urea and measured with LC-MS. General method for detection of nucleotide sugar levels from the enzymatic assays with LC-MS: For all enzymatic reactions described above the following LC-MS procedure was used: For each sample, 10 μL was analyzed by targeted ion-pairing LC-MS/MS using an Agilent 1290 Infinity UPLC and 6490 A QQQ Mass Spectrometer. G4220A Binary Pump was set to deliver mobile phase A (10 mM tributylamine, 12 mM acetic acid, 2 mM acetylacetone, 3% MeOH) and B (10 mM tributylamine, 12 mM acetic acid, 2 mM acetylacetone, 3% MeOH, 80% acetonitrile) at 0.5 mL/min. Injection volume was set to 1 μL. Gradient separation was performed with initial setting of 0% B, 20% B at 1 min, 30% B at 9 min, 100% B at 10 min, followed by 3.5 min re-equilibration at 0% B. QQQ mass spectrometer operated in multiple-reaction monitoring MRM mode using transitions generated in silico by the use of a script written in Python (3.9.2), an RDkit library (2020.09.5), and chemical structures of the possible GMDS reaction intermediates as the input^25^. Data were processed in Skyline 20.2. Plots of the data were generated Python (v 3.9.2) using Pandas (v 1.1.2) and Seaborn (v 0.11) libraries or (for the IC50-measurements) in Microsoft Excel 365 and Graphpad Prism 5.

GMDS digestion and proteomics: Either GDP-d-Rha6F_2_ (1.85 mM), GDP-Man (1.85 mM) or an equal volume of water was incubated with GMDS (185 µM) in HEPES (10 mM, 150 mM NaCl, pH 7.5), with a total volume of 60 μl. After 16 h, the inhibitor was washed away with four successive washes with a tenfold excess of Tris (10 mM; pH 8.0) using a amicon ultra-0.5 centrifugal filter unit (10000 x g, 10 Kd MWCO). After lyophilization, the samples were dissolved in 7.5 μl Tris (10 mM; pH 8.0), denatured by addition of 7.5 μl of Urea (8 M) and reduced by addition of 7.5 μl DTT (100 mM). After 30 min, 7.5 μl of 2-Chloroacatemide (50 mM) in (NH_4_)_2_CO_3_ (50 mM) was added and incubated in the dark for 30 mins. The solution was diluted by addition of 100 μl (NH_4_)_2_CO_3_ (50 mM), 3 μl of Trypsin (Promega, Sequencing Grade Modified) was added and incubated for 16 h at 37 ^o^C. The samples were filtered with a 0.22 µm syringe filter before LCMS analysis. The tryptic digests were analyzed with LCMS on a high-resolution timsTOF (Bruker) in positive ion mode. The digests were eluted using a linear gradient of 0-100% B in 30 min, of A: 0.1% formic acid in water and B: 0.1 % Formic Acid in MeCN, with a flow rate of 300 μl/min using an Extend C18 RRHD column (2.1 ×150 mm, Agilent) at 40 ^o^C on a UHPLC system (Elute UHPLC). Included at the end of the LC run, the flow is switched to 0.1% sodium formate in water using the syringe pump of the timsTOF operating at 3 μl/min. The MS data were processed using Compass Data Analysis (Bruker) version 5.1. The spectra were calibrated with the internal calibrant sodium formate in HPC mode. Deconvolution of the tryptic peptides was done using the Peptides/Small Molecules deconvolution with the cutoff set to 0.05%. The exported data was subjected to a Mascot MS/MS ion search against the SwissProt data bank using the following parameters: Trypsin, maximum missed cleavages = 1, variable modifications = carbamylation. Extracted Ion chromatograms were generated (with a mass width of ±0.1 m/z) corresponding to the identified tryptic peptide FYQASTSELYGK, spanning the active site sequence 150–161, with a mass increase corresponding to the covalent addition of GDP-d-Rha6F_2_ or metabolites thereof at charge states (+1, +2, +3). The tryptic digest resulting from the incubation of GDP-d-Rha6F_2_, did not have masses corresponding to covalent addition of GDP-d-Rha6F_2_ in a higher abundance then the GMDS or GMDS treated with GDP-Man controls.

### In silico modelling

Protein preparation for docking: The crystal structure of GMDS in complex with 4-fluoro-α-D-mannopyrranosyl guanoside was downloaded from the PDB-redo database (https://pdb-redo.eu/, PDB code 6GPJ)^23^ and loaded into MOE 2020. The structure was preprocessed by deleting all non- NADP^+^ and G4F ligands. Next, the potential was set up to AMBER14:EHT as force field with R-field solvation model. Addition of hydrogen atoms, removal of water molecules farther than 4.5 Å away from ligand or receptor, correction of library errors and tethered energy minimization up to a gradient of 0.1 kcal mol^−1^ Å^2^ was performed using MOE’s 2020 QuickPrep module. Afterwards, only the C-chain residues, ligands and solvent molecules were kept. To mimic each intermediate step in the reaction pathway of GMDS, changes were made to both the receptor and ligands, based on earlier finding by Pfeiffer et al. using the MOE 2020 Builder module^23^. Regarding the protein this concerned the (de)protonation of Tyr179 and Glu157 and regarding the ligands the presence of either NADP^+^ or NADPH. After making the adjustments, MOE’s 2020 QuickPrep module was ran again without protonation, changing the final RMS gradient for the energy minimization to 1×10^−5 ^kcal mol^−1^ Å^2^. Ligand preparation: Ligand structures were drawn in ChemDraw 18.1, imported into a MOE 2020 database in sdf-file format, and energy minimized up to a gradient of 1×10^−5^ using the MMFF94x force field. The saved mdb-file was used for the docking studies. Docking: The ligands were docked into the appropriate protein model, using cocrystalized 4-fluoro-GDP-mannose as template for placement. After 30 placements the placements were refined to 5 poses using the Induced Fit method with GBVI/WSA rescoring. From the 5 poses, the best pose was chosen by visual inspection for each ligand by a combination of their displacement compared to the template ligand and the calculated score.

### Synthetic procedures and abbreviations

^1^H and ^13^C NMR spectra were recorded on a Varian Iinova 400 MHz or Bruker Avance III 500 MHz spectrometer and analyzed using MestReNova v14.1.0-24037. Chemical shifts are reported in parts per million (ppm) relative to tetramethylsilane (TMS) as the internal standard. NMR data is presented as follows: Chemical shift, multiplicity (s = singlet, bs = broad singlet, d = doublet, t = triplet, dd = doublet of doublet, dt = doublet of triplet, m = multiplet and/or multiple resonances), integration, coupling constant in Hertz (Hz). All NMR signals were assigned on the basis of ^1^H, ^13^C, ^19^F NMR, COSY and HSQC experiments. Mass spectra were recorded on a JEOL JMS-T100CS AccuTOF mass spectrometer. Automatic column chromatography was performed on Biotage Isolera Spektra One, using SNAP cartridges 10-50 g filled with normal silica (Biotage, 30–100 μm, 60 Å) or water resistant iatro beads. Microwave reactions were perfOCH_3_d on a Biotage Initiator 4.1.3. TLC analysis was conducted on TLC Silicagel, 60, F254, Merck, with detection by UV absorption (254 nm) where applicable, and by spraying with 20% sulfuric acid in methanol followed by charring at ~150 °C or by spraying with a solution of (NH-4)_6_Mo_7_O_24_^.^H_2_O (25 g l-1) in 10% sulfuric acid in methanol followed by charring at ~300 °C. DCM, ACN and Tol were freshly distilled. All reactions were carried out under an argon atmosphere. All synthesized samples that were used for cell tests were dissolved in water or a mixture of dioxane/water and subsequently lyophilized. Abbreviations: Ac_,_ acetyl; ACN, Acetonitrile; AcOH Acetic acid; aq., aqueous; DCM dichloromethane; DMAP, dimethylaminopyridine; DMF, *N*,*N*-Dimethylformamide; DMP, Dess–Martin periodinane; EtOAc, ethyl acetate; eq., equivalent; Hept, heptane; hrs, hours; mCPBA, meta-chloroperoxybenzoic acid; min, minutes; NBS, N-bromosuccinimide; Pyr, pyridine; r.t., room temperature; sat., saturated; TBDMS, tert-Butyldiphenylsilyl; TEA, triethylamine; THF, tetrahydrofuran; TIPS, triisopropylsilyl; TMS, trimethylsilyl; Tol, toluene. Detailed synthetic procedures and data is available in the Supplementary Information.

### Reporting Summary

Further information on research design is available in the Nature Research Reporting Summary linked to this article.

## Supplementary information

Supplementary Information

Reporting Summary

## Data Availability

The authors declare that the data supporting the findings of this study are available within the paper and its supplementary information files. All raw data files are stored on the Radboud University data management server and available upon request. For in silico experiments we obtained data from the PDB-redo database (https://pdb-redo.eu/, PDB code 6GPJ). The GMDS digestion data was subjected to a Mascot MS/MS ion search against the SwissProt data bank (https://www.matrixscience.com/).
