# Supplementary material for: Fluorinated rhamnosides inhibit cellular fucosylation
Source: Nat Commun. 2021 Dec 2;12:7024. doi: 10.1038/s41467-021-27355-9 (PMC8640046; doi:10.1038/s41467-021-27355-9)
Supplement: Supplementary file 1 — Supplementary Information [file 41467_2021_27355_MOESM1_ESM.pdf]

Fluorinated rhamnosides inhibit cellular fucosylation.
Supporting information

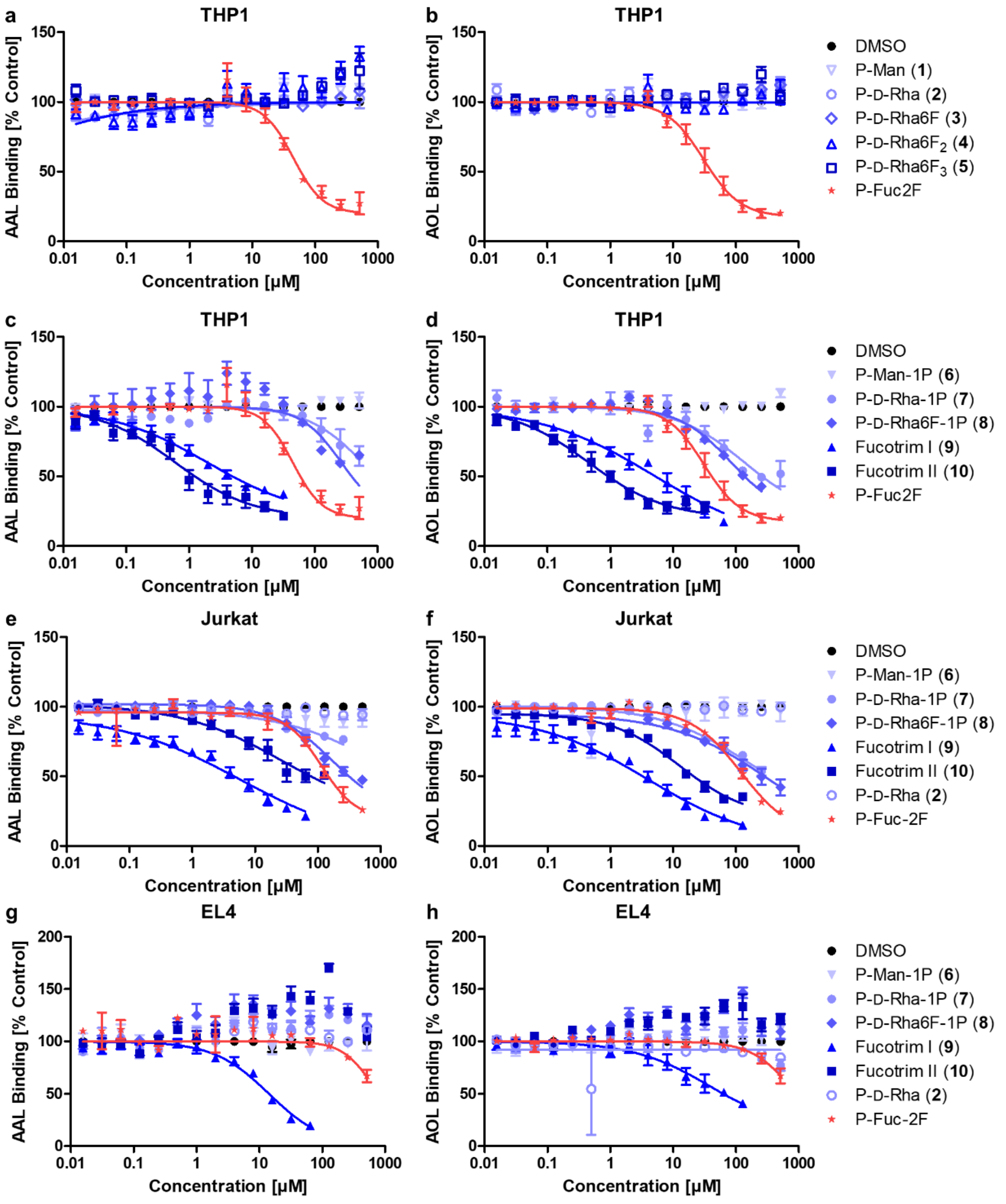

Supplementary Figure 1: Evaluation of defucosylation potency. Cell lines THP-1 (a-d), Jurkat (e-f) and EL4 (g-h) were cultured for 3 days with 0–512 µM compound or DMSO control. The cells were stained with two fucose specific lectins, AAL (a, c, e, g) & AOL (b, d, f, h) and analysed by flow cytometry, presented as mean percentage lectin binding normalized to control ± SEM. N ≥ 3 biologically independent experiments of 10.000 gated cells per sample and N = 2 technical replicates for each experiment.

Fluorinated rhamnosides inhibit cellular fucosylation.
Supporting information

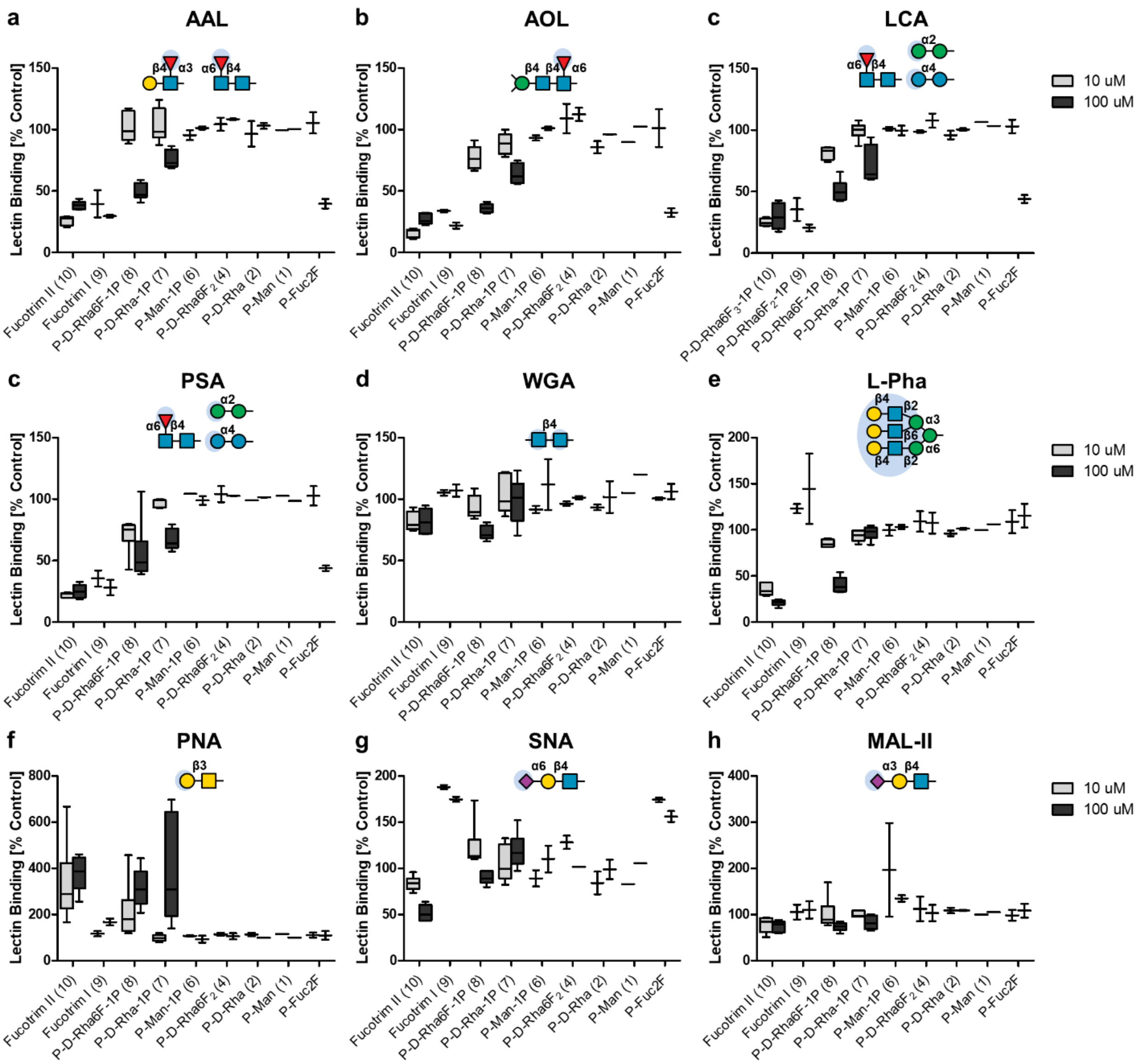

Supplementary Figure 2: Effect of **1**, **2**, **4**, **6**–**10**, P-Fuc2F and DMSO control on total cell surface glycosylation. THP-1 cells treated for three days with 10 or 100 µM compound or DMSO control were stained with a panel of biotinylated lectins and streptavidin-PE. The Box and Whisker Plots with 5–95 percentiles show binding of AAL (a), AOL (b), LCA (c), PSA (d), WGA (e), L-Pha (f), PNA (g), SNA (h) and MAL-II (i). Schematic representation of binding affinity per lectin is indicated by blue shading. N ≥ 3 biologically independent experiments of 10.000 gated cells per sample and N = 1 technical replicates for each experiment, presented as mean fluorescent intensity relative to DMSO control ± SEM.

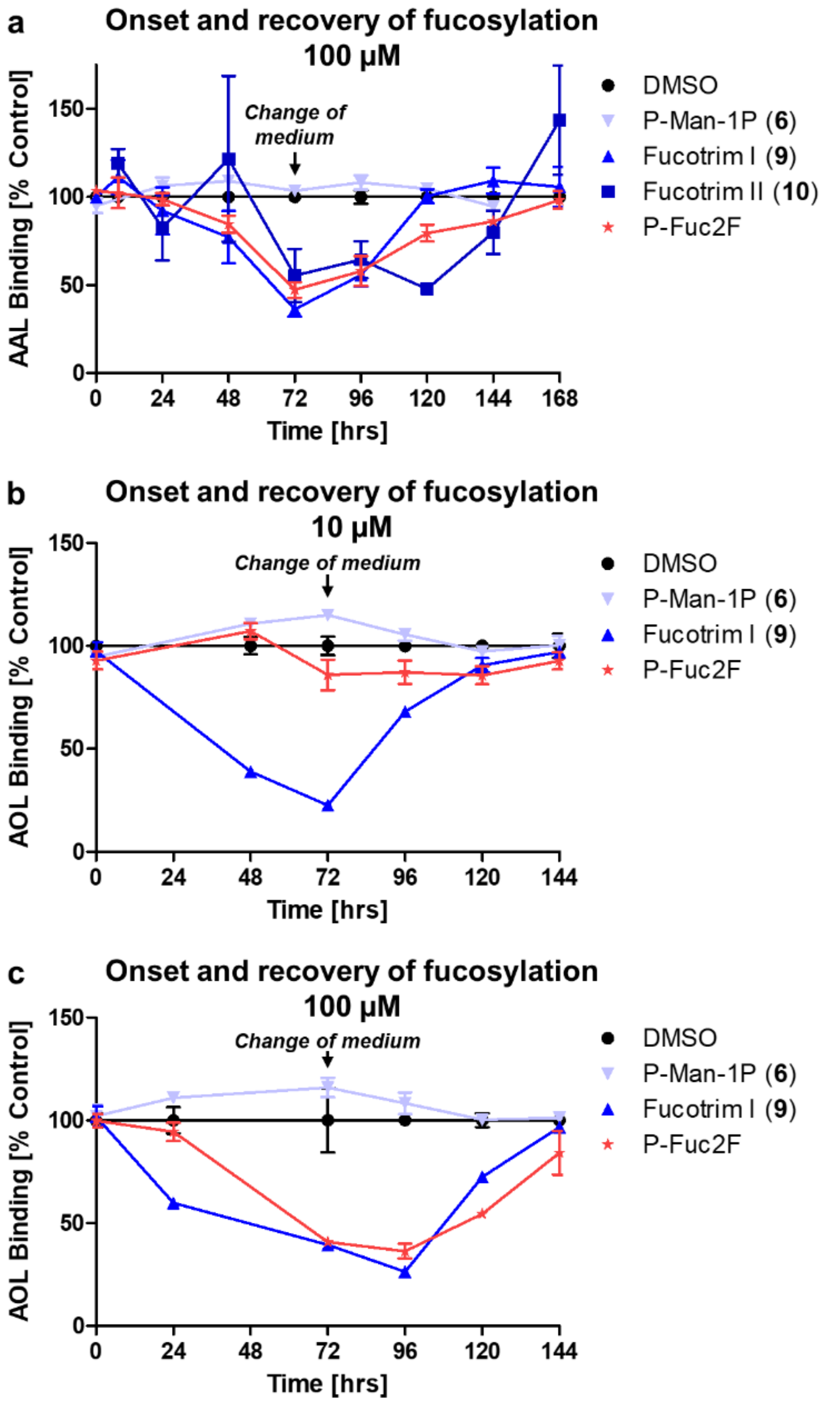

Supplementary Figure 3: Onset and recovery of defucosylation. THP1 cells were incubated with 10 µM (b, Figure 3d) or 100 µM (a,c) compound or DMSO control and fucosylation levels were determined with AAL (a) and AOL (b,c) lectins for six days. N ≥ 3 biologically independent experiments of 10.000 gated cells per sample and N = 2 technical replicates for each experiment, presented as mean fluorescent intensity relative to DMSO control ± SEM.

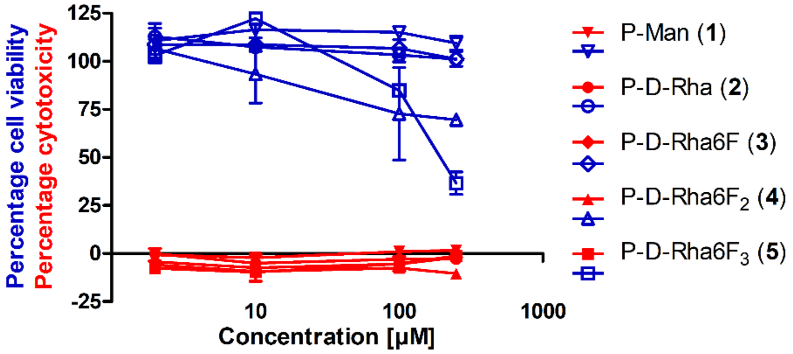

Supplementary Figure 4: Evaluation of effect on viability (blue) and cytotoxicity (red). THP1 cells were incubated with 2, 10, 100 or 250 μM **1**–**5** and analyzed with an XTT and LDH assay respectively, presented as mean percentage cell viability or cytotoxicity compared to DMSO control ± SEM. N = 1 biologically independent experiments of 10.000 gated cells per sample and N = 2 technical replicates.

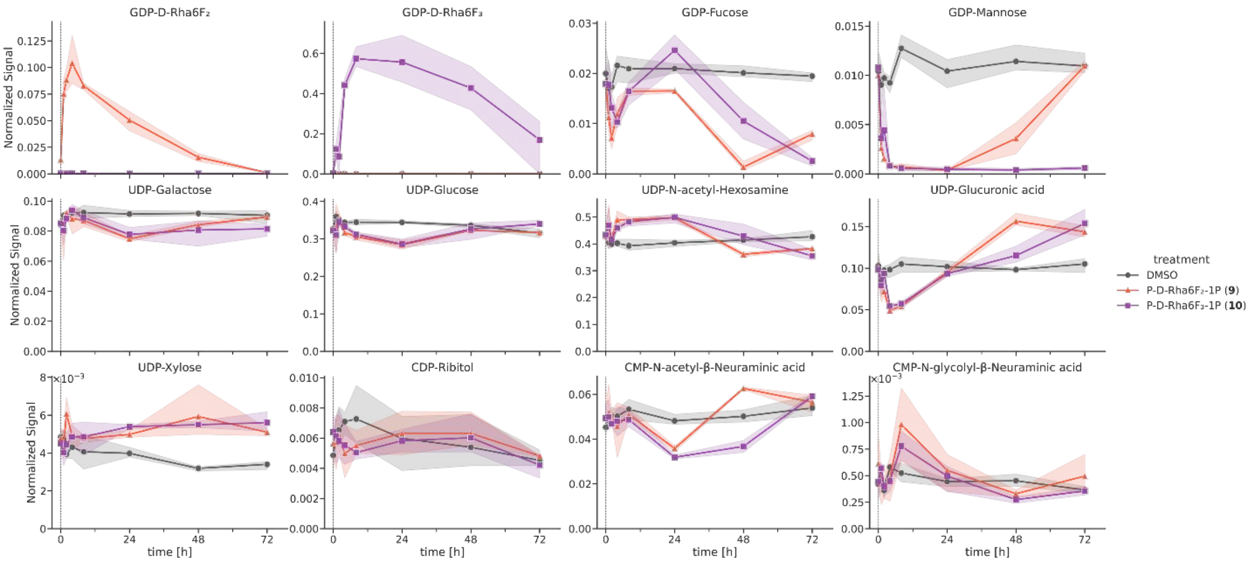

Supplementary Figure 5: Full nucleotide sugar analysis. THP-1 cells were incubated for indicated time points with 10 µM Fucotrim I or II or with DMSO control. After sample preparation, the different nucleotide levels were analyzed using reverse-phase ion pairing chromatography coupled to a triple quadrupole mass spectrometer operating in negative ion mode and presented as their abundance in the nucleotide sugar pool, presented as mean (line) with 95% confidence interval (shading). N = 3 biologically independent experiments and N = 1 technical replicates.

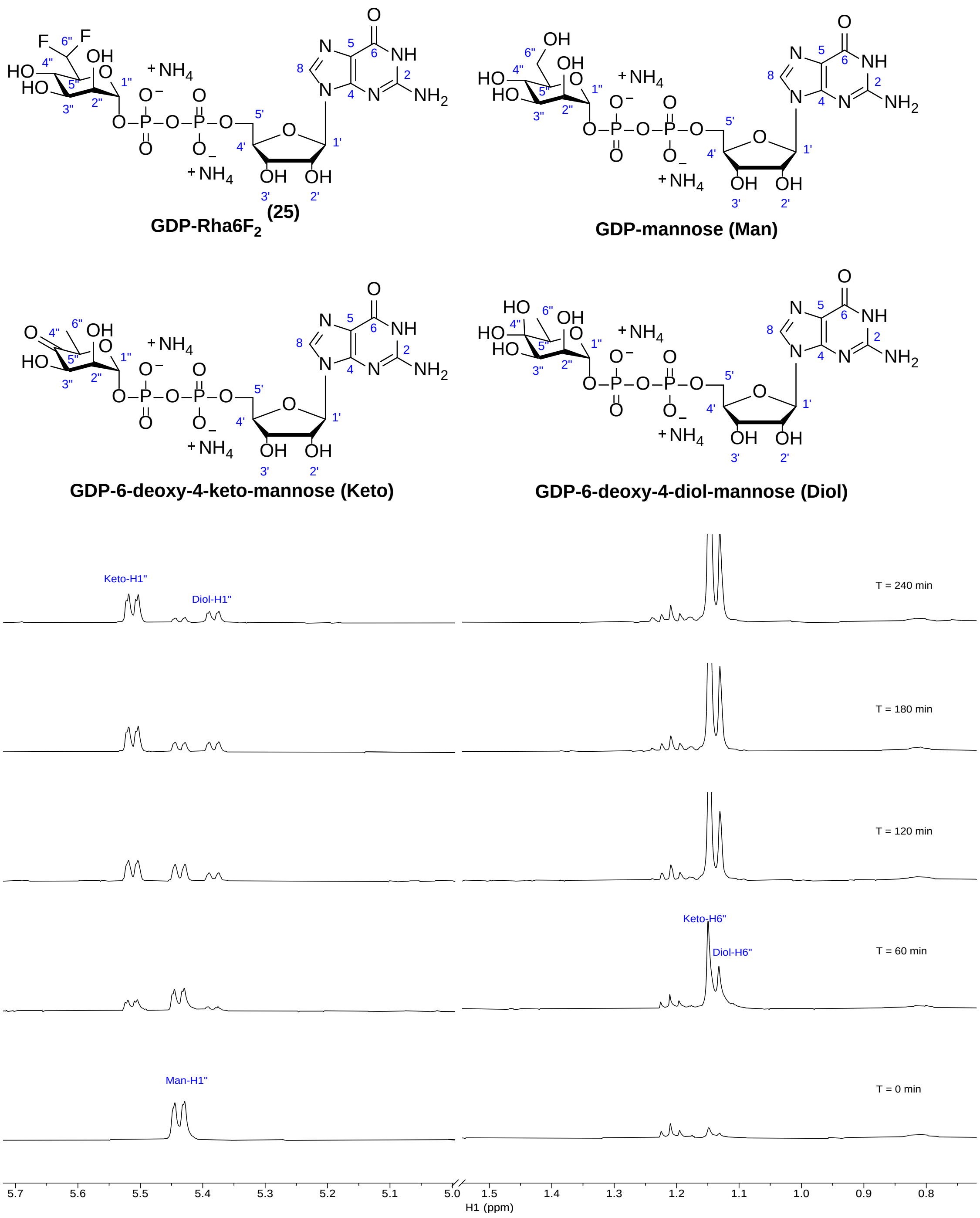

Supplementary Figure 6. ^{1}H NMR time course experiment of GDP-mannose and GMDS at 25℃, forming GDP-6-deoxy-4-keto-mannose and its corresponding hydrate, GDP-6-deoxy-4-diol-mannose.

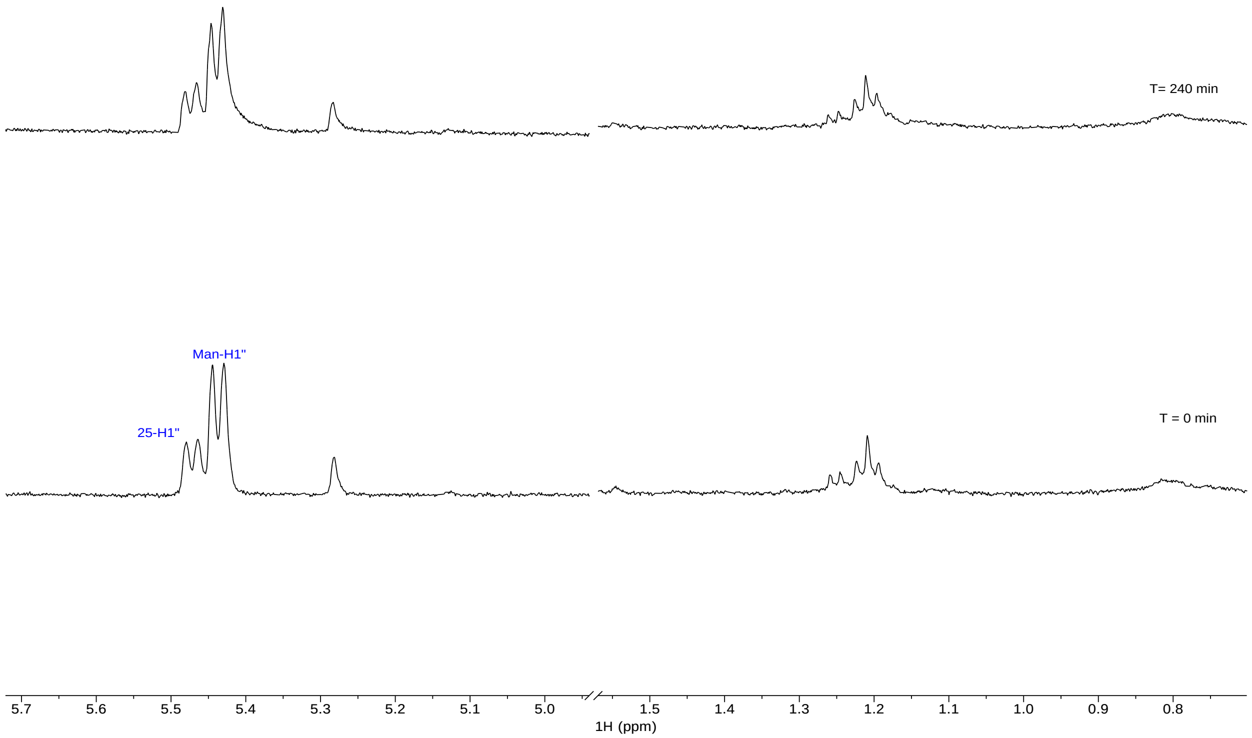

Supplementary Figure 7: ^{1}H NMR time course experiment of GDP-mannose, GDP-Rha6F$_2$ (**25**) and GMDS at 25℃. No conversion of GDP-mannose was observed. N = 1 experiment.

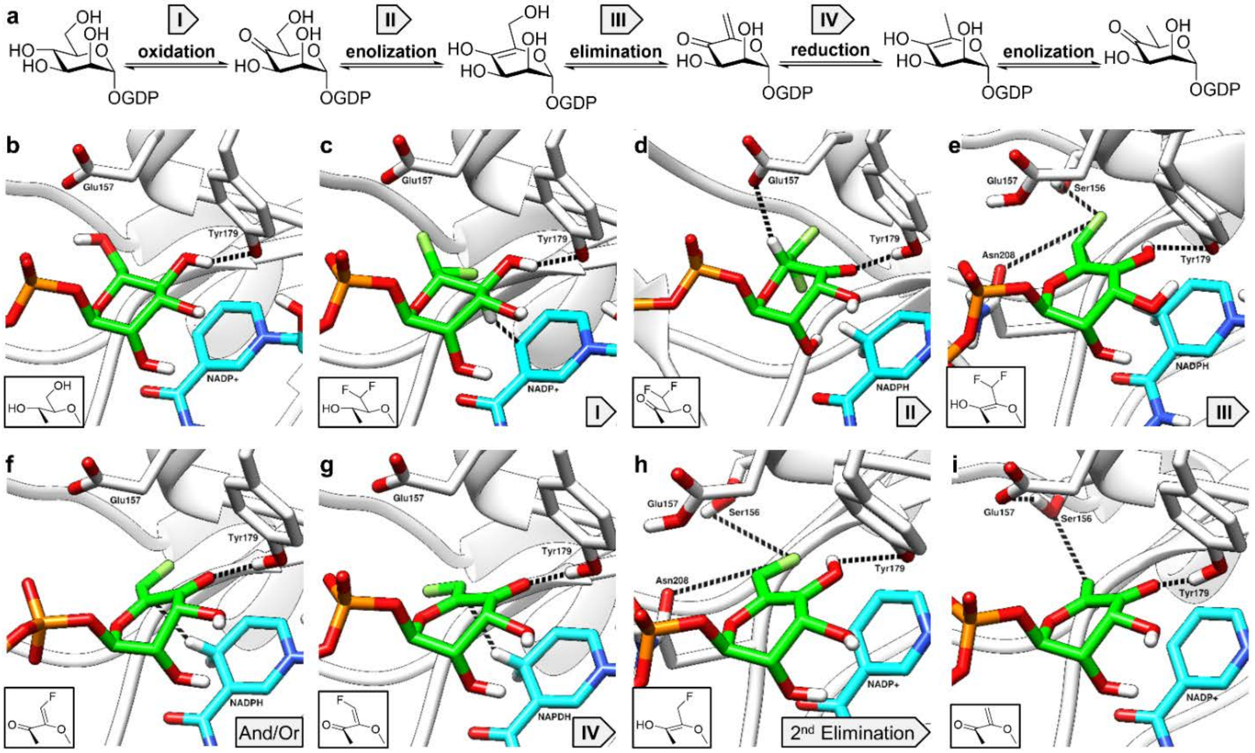

Supplementary Figure 8: *In silico* evaluation of Fucotrim I in GMDS. (a) Endogenous mechanism of action for human GMDS. (b) Natural substrate GDP-mannose (green carbons) was docked at the 4-fluoro-GDP-mannose (G4F) binding site in human GMDS (6GPJ[1], white carbons) in which also $NADP^+$ (cyan carbons) was cocrystalized. (c–i) Unnatural substrate GDP-D-Rha6F_2 (c, green carbons) and the proposed metabolic intermediates (d–i, green carbons) were docked in the same active site. For each structure, the active site residues Glu157 and Tyr179 were first protonated or deprotonated and either NADPH or $NADP^+$ was present accordingly with the known mechanism of action on GDP-mannose. Black dotted lines depict expected interactions between atoms leading to a new product, based on the natural mechanism.

A) GMDS

1 MAHAPARCPS ARGSGDGEMG KPR**NVALITG** **ITGQDGSYLA** **EFLLEKGYEV**
51 **HGIVR**R**SSSF** **NTGR**IEHLYK **NPQAHIEGNM** **KLHYGDLTDS** **TCLVKIINEV**
101 **KPTEIYNLGA** **QSHVKISFDL** **AEYTADVDGV** **GTLR**LLDAVK **TCGLINSVKF**
151 **YQASTSELYG** **KVQEIPQKET** **TPFYPR**SPYG AAKLYAYWIV VNFR**EAYNLF**
201 **AVNGILFNHE** **SPRRGANFVT** **R**KISRSVAK**I** **YLGQLECFSL** **GNLDAK**RDWG
251 HAK**DYVEAMW** **LMLQNDEPED** **FVIATGEVHS** **VR**EFVEK**SFL** **HIGKTIVWEG**
301 **KNENEVGR**CK ETGK**VHVTVD** **LKYYRPTEVD** **FLQGDCTK**AK QKLNWKPR**VA**
351 **FDELVREMVH** **ADVELMR**TNP NA

B) GMDS + GDP-Man

1 MAHAPARCPS ARGSGDGEMG KPR**NVALITG** **ITGQDGSYLA** **EFLLEKGYEV**
51 **HGIVR**R**SSSF** **NTGR**IEHLYK **NPQAHIEGNM** **KLHYGDLTDS** **TCLVKIINEV**
101 **KPTEIYNLGA** **QSHVKISFDL** **AEYTADVDGV** **GTLR**LLDAVK **TCGLINSVKF**
151 **YQASTSELYG** **KVQEIPQKET** **TPFYPR**SPYG AAKLYAYWIV VNFR**EAYNLF**
201 **AVNGILFNHE** **SPRRGANFVT** **R**KISRSVAK**I** **YLGQLECFSL** **GNLDAK**RDWG
251 HAK**DYVEAMW** **LMLQNDEPED** **FVIATGEVHS** **VR**EFVEK**SFL** **HIGKTIVWEG**
301 **KNENEVGR**CK ETGK**VHVTVD** **LKYYRPTEVD** **FLQGDCTK**AK QKLNWKPR**VA**
351 **FDELVREMVH** **ADVELMR**TNP NA

C) GMDS + GDP-D-Rha6F$_2$

1 MAHAPARCPS ARGSGDGEMG KPR**NVALITG** **ITGQDGSYLA** **EFLLEKGYEV**
51 **HGIVR**R**SSSF** **NTGR**IEHLYK **NPQAHIEGNM** **KLHYGDLTDS** **TCLVKIINEV**
101 **KPTEIYNLGA** **QSHVKISFDL** **AEYTADVDGV** **GTLR**LLDAVK **TCGLINSVKF**
151 **YQASTSELYG** **KVQEIPQKET** **TPFYPR**SPYG AAKLYAYWIV VNFR**EAYNLF**
201 **AVNGILFNHE** **SPRRGANFVT** **R**KISRSVAK**I** **YLGQLECFSL** **GNLDAKRDWG**
251 **HAKDYVEAMW** **LMLQNDEPED** **FVIATGEVHS** **VR**EFVEK**SFL** **HIGKTIVWEG**
301 **KNENEVGR**CK ETGK**VHVTVD** **LKYYRPTEVD** **FLQGDCTK**AK QKLNWKPR**VA**
351 **FDELVREMVH** **ADVELMR**TNP NA

Supplementary Figure 9: Sequence coverage (in bold) after the tryptic digest of A) GMDS, B) GMDS treated with GDP-Man and C) GMDS treated with GDP-D-Rha6F$_2$ resulting from a Mascot database search engine. In blue, the tryptic peptide spanning the active site serine 156.

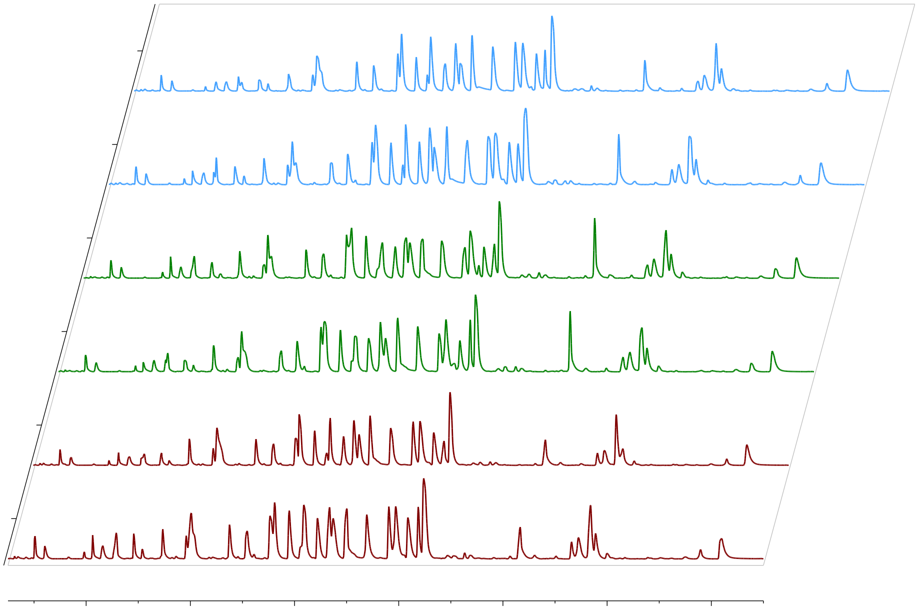

Supplementary Figure 10: HPLC traces in duplicate of digestions of GMDS (traces 1&2, red), GMDS incubated with GDP-Man (traces 3&4, green) or GMDS incubated with GDP-D-Rha6F$_2$ (traces 5&6, blue).

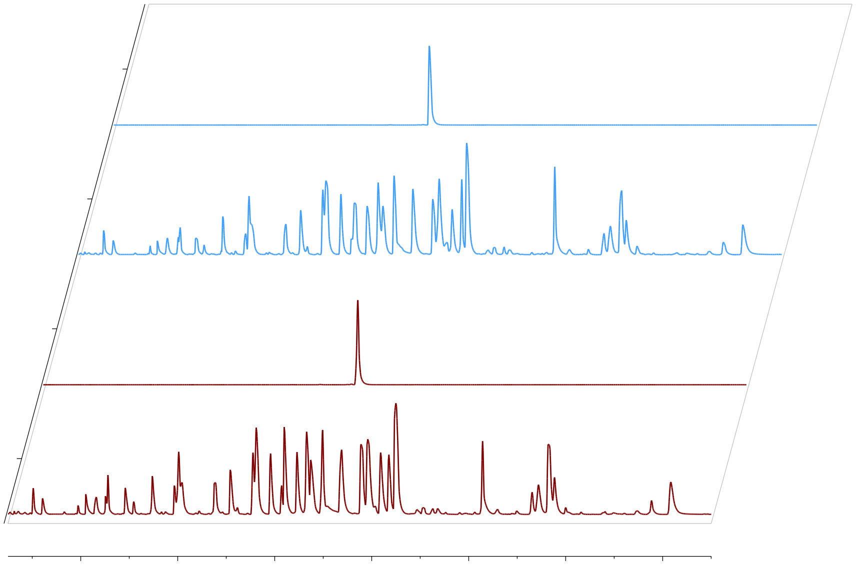

Supplementary Figure 11: HPLC trace of digestions of GMDS (trace 1, red), the extracted ion chromatograms (EIC) for 697.33 *m/z* (trace 2, red) GMDS incubated with GDP-D-Rha6F$_2$ (trace 3, blue) and the EIC of 697.33 *m/z* (trace 4, blue). The mass of 697.33 *m/z* corresponds to FYQASTSELYGK, the active site sequence 150-161. N=2 experiments, individual traces shown.

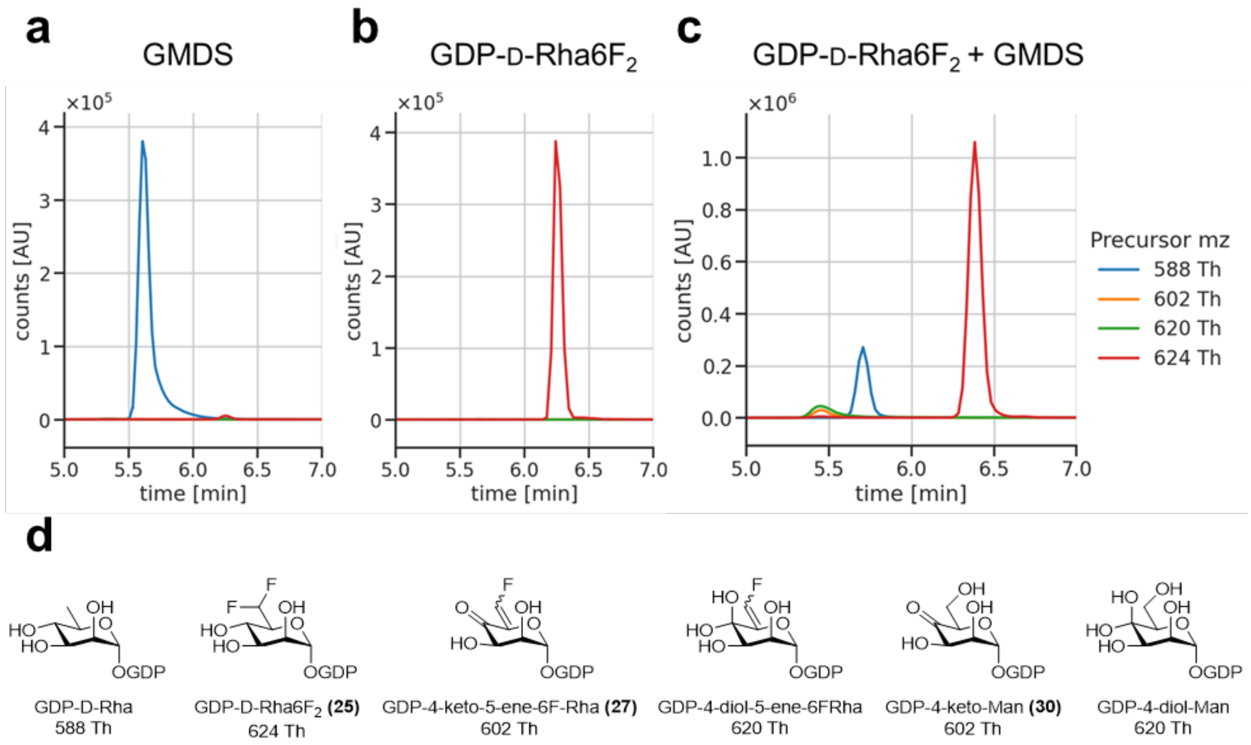

Supplementary Figure 12: Determining possible inhibitor metabolites. Shown chromatograms with precursor ion traces (negative mode) at retention times between 5 and 7 minutes of **A)** GMDS (100 µM) incubated for 21 hours in buffer (50 mM phosphate buffer, pH 7.5), **B)** GDP- D-Rha6F$_2$ (**25**), incubated overnight in buffer and **C)** GMDS (100 µM) incubated for 21 hours with D-Rha (**25**) (100 µM) in buffer. **D)** Structures of the intermediates shown in Figure 5d and their theoretical m/z (negative mode, M-H). Both mixtures containing GMDS show a GDP-sugar with precursor m/z of 588. This is likely GDP-6-deoxy-hexose, bound in GMDS during protein expression. N = 1 experiment.

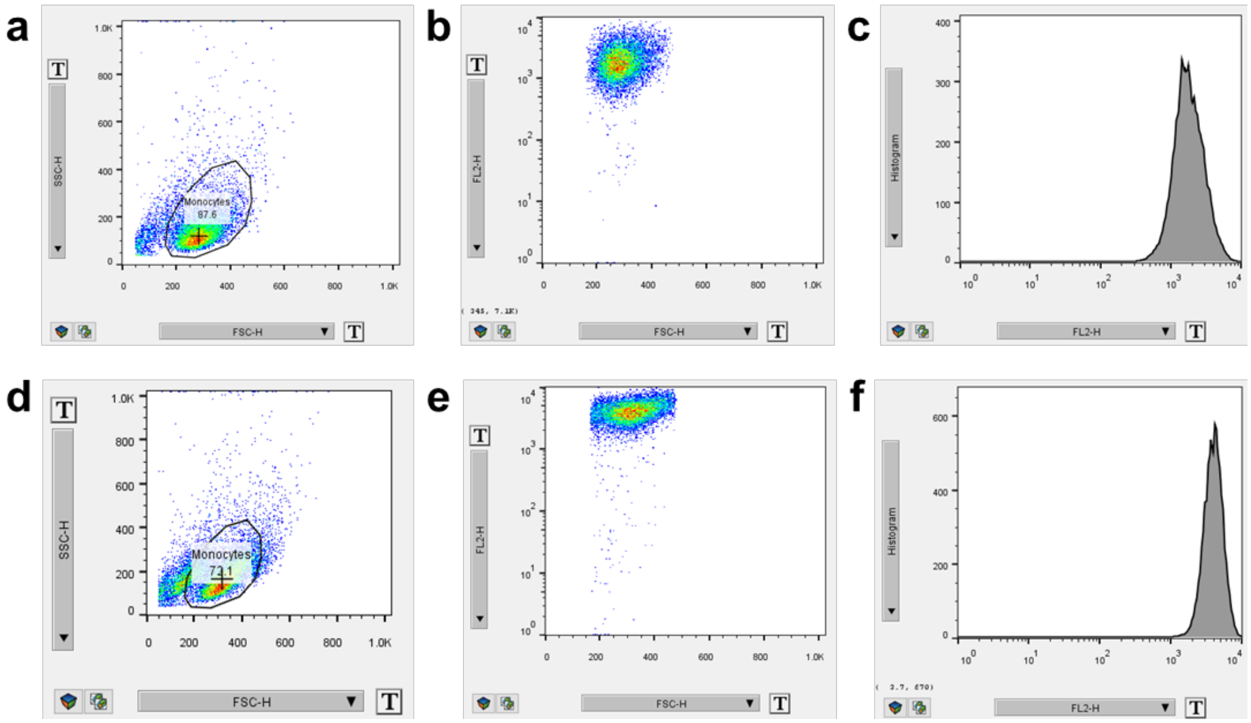

Supplementary Figure 13: Example of gating strategy, representative for the gating strategies used in the manuscript. Data shown correspond to THP-1 cells, treated with either 8 µM FucoTrim I (a–c) or to the respective dose of 1/12800 v/v DMSO (d–f) of which the mean fluorescence shown in (c) and (f) respectively were used for the data in Table 1 and Figure 3b. (a) and (c) show the FSC-H/SSC-H plot in which singular monocytes were gated from debris. (b) and (d) show FSC-H/FL2-H (channel used to measure the streptavidin-phycoerythrin fluorescence) and (c) and (f) show histograms of the streptavidin-phycoerythrin fluorescence of the populations.

Synthetic procedures

General procedure A: Selective 1-deacetylation (Supplementary Figure 14)

Compounds **2**–**4** (1 eq.) were dissolved DMF (0.3 M) and hydrazine acetate (1.2 eq.) was added at 0 ℃. After stirring for 5 minutes, the mixture was stirred till completion at r.t. which was typically after 3 hrs. The mixture was diluted with DCM, washed with sat. aq. $NaHCO_3$ and brine. The organic layer was dried over anhydrous Na_2SO_4, filtered concentrated *in vacuo*. The residue was purified by silicagel flash column chromatography (EtOAc in Hept) to afford **11**–**13**.

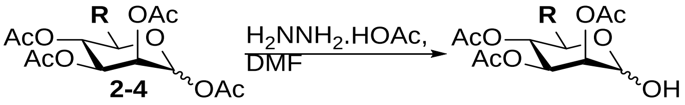

Supplementary Figure 14: General procedure A: Selective 1-deacetylation.

General procedure B: 1-Phosphorylation (Supplementary Figure 15)

Similar as described previously,[2] 1-hydroxy-derivatives of **6**–**10** (1 eq.) and di-(S-acetyl-2-thioethyl)-N,N-diethylphosphoroamidite **23** (1.1 eq) were dissolved in in ACN (0.35 M) at 0 °C and 1*H*-tetrazole (1.4 eq, 0.45 M in acetonitrile) was slowly added. After full conversion, typically after 1 hr, mCPBA (2.4 eq, 77% wt.) was added at 0 °C and stirred for 30 min. The mixture was diluted with EtOAc and 10% aq. Na_2SO_3 and the organic layer was washed with sat. aq. $NaHCO_3$ and brine. The aqueous phases were extracted two more times with fresh EtOAc and the combined organic layers were dried over anhydrous Na_2SO_4, filtered concentrated *in vacuo*. The residue was purified by silicagel flash column chromatography (EtOAc in Hept) to afford **6**–**10**.

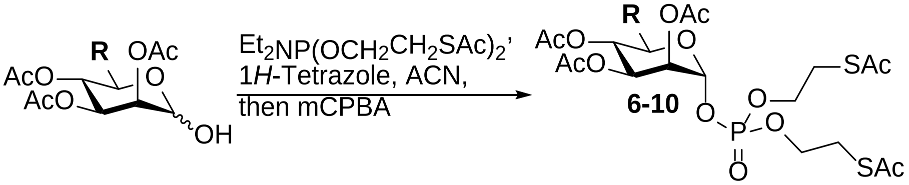

Supplementary Figure 15: General procedure B: 1-Phosphorylation

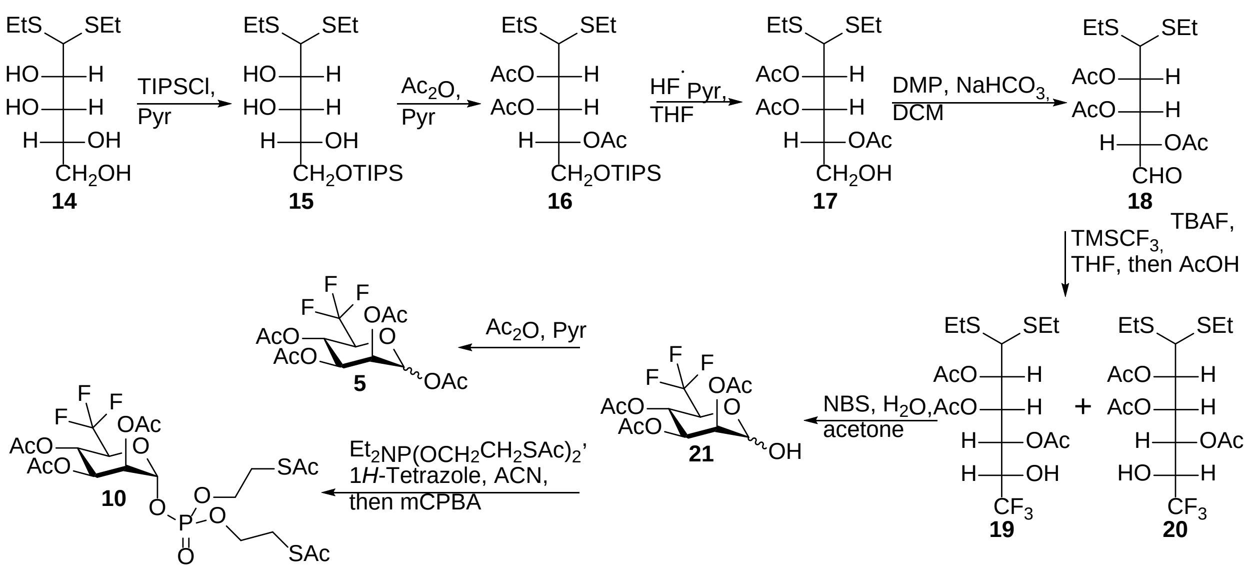

Supplementary Figure 16: Synthetic route towards trifluoro derivatives **5** and **10**.

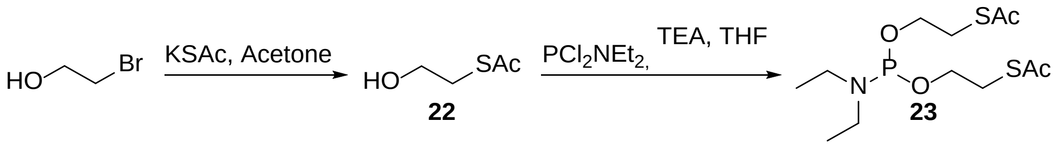

Supplementary Figure 17: Synthetic route towards phosphoramidite reagent **23**.

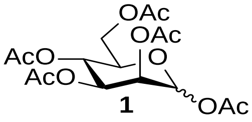

1,2,3,4,6-penta-O-acetyl-D-mannopyranoside (P-D-Man, 1). Synthesis was performed as described previously and the data is identical to the data previously reported.[3] **TLC:** (60:40, EtOAc:Hept v/v) R_f = 0.53. **^{1}H NMR** (500 MHz, $CDCl_3$, major anomer) δ 6.09 (d, *J* = 1.9 Hz, 1H, H1), 5.36 – 5.34 (m, 2H, H3 & H4), 5.27 – 5.26 (m, 1H, H2), 4.29 (dd, *J* = 12.4, 4.8 Hz, 1H, H6a), 4.11 (dd, *J* = 12.4, 2.5 Hz, 1H, H6b), 4.06 (dtd, *J* = 6.2, 4.6, 2.5 Hz, 1H, H5), 2.18 (s, 3H, CH_3 Ac), 2.17 (s, 3H, CH_3 Ac), 2.10 (s, 3H, CH_3 Ac), 2.06 (s, 3H, CH_3 Ac), 2.01 (s, 3H, CH_3 Ac); **^{13}C NMR** (126 MHz, $CDCl_3$, major anomer) δ 170.73 (CO Ac), 170.08 (CO Ac), 169.83 (CO Ac), 169.63 (CO Ac), 168.15 (CO Ac), 90.71 (C1), 70.72 (C5), 68.85 (C3), 68.45 (C2), 65.66 (C4), 62.21 (C6), 20.96 (CH_3 Ac), 20.87 (CH_3 Ac), 20.81 (CH_3 Ac), 20.76 (CH_3 Ac), 20.74 (CH_3 Ac); **HRMS** *(m/z)*: $[M+Na]^+$ calcd for $C_{16}H_{22}NaO_{11}$, 413.10598; found, 413.10835.

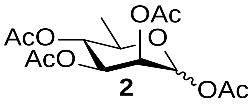

1,2,3,4-tetra-O-acetyl-D-rhamnopyranoside (P-D-Rha, 2). D-Rhamnose (230 mg; 1.52 mmol) was dissolved in pyridine (2.2 mL; 18 eq.) and acetic anhydride (1.7 mL; 12 eq.) was slowly added at 0℃. The mixture was stirred for 16 hrs while slowly warming to r.t.. The mixture was concentrated *in vacuo* and the residue was dissolved in DCM, washed with aq. HCl (1 M), sat. aq.

$NaHCO_3$ and brine. The organic layer was dried over anhydrous $MgSO_4$, filtered and concentrated *in vacuo*. The residue was purified by silicagel flash column chromatography (0% → 20 EtOAc in Hept) to afford **2** (466 mg; 1.40 mmol; qu.). **TLC:** (50:50, EtOAc:Hept v/v) R_f = 0.44. **^{1}H NMR** (500 MHz, $CDCl_3$, major anomer) δ 6.02 (d, *J* = 1.9 Hz, 1H, H1), 5.32 – 5.29 (m, 1H, H3), 5.25 (dd, *J* = 3.5, 2.0 Hz, 1H, H2), 5.12 (t, *J* = 10.0 Hz, 1H, H4), 3.94 (dq, *J* = 9.8, 6.2 Hz, 1H, H5), 2.17 (s, 3H, CH_3 Ac), 2.16 (s, 3H, CH_3 Ac), 2.07 (s, 3H, CH_3 Ac), 2.01 (s, 3H, CH_3 Ac), 1.24 (d, *J* = 6.2 Hz, 3H, 3x H6); **^{13}C NMR** (126 MHz, $CDCl_3$, major anomer) δ 170.17 (CO Ac), 169.92 (CO Ac), 169.90 (CO Ac), 168.47 (CO Ac), 90.76 (C1), 70.59 (C4), 68.89 (C3), 68.83 (C5), 68.76 (C2), 21.02 (CH_3 Ac), 20.89 (CH_3 Ac), 20.87 (CH_3 Ac), 20.79 (CH_3 Ac), 17.56 (C6). **HRMS** *(m/z)*: $[M+Na]^+$ calcd for $C_{14}H_{20}NaO_9$, 355.10050; found, 355.10295.

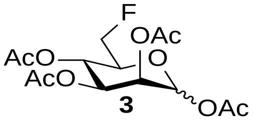

3 **1,2,3,4-tetra-O-acetyl-6-fluoro-D-rhamnopyranoside (P-D-Rha6F, 3)**. Synthesis was performed as described previously and the data is identical to the data previously reported.[4] **TLC:** (60:40, EtOAc:Hept v/v) R_f = 0.53. **^{1}H NMR** (500 MHz, $CDCl_3$, major anomer) δ 6.10 (d, *J* = 1.9 Hz, 1H, H1), 5.39 – 5.36 (m, 2H, H3 & H4), 5.27 (td, *J* = 2.2, 0.6 Hz, 1H, H2), 4.54 – 4.42 (m, 2H, H6a & H6b), 4.08 – 3.99 (m, 1H, H5), 2.18 (s, 3H, CH_3 Ac), 2.17 (s, 3H, CH_3 Ac), 2.08 (s, 3H, CH_3 Ac), 2.02 (s, 3H, CH_3 Ac); **^{13}C NMR** (126 MHz, $CDCl_3$, major anomer) δ 170.11 (CO Ac), 169.87 (CO Ac), 169.57 (CO Ac), 168.14 (CO Ac), 90.62 (C1), 81.29 (d, *J* = 175.8 Hz, C6), 71.42 (d, *J* = 19.5 Hz, C5), 68.78 (C3), 68.36 (C2), 65.24 (d, *J* = 6.6 Hz, C4), 20.94 (CH_3 Ac), 20.83 (CH_3 Ac), 20.74 (2x CH_3 Ac); **^{19}F NMR** (470 MHz, $CDCl_3$, major anomer) δ -232.46 (td, *J* = 47.1, 22.9 Hz, 6F). **HRMS** *(m/z)*: $[M+Na]^+$ calcd for $C_{14}H_{19}FNaO_9$, 373.09108; found, 373.09255.

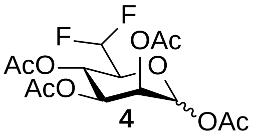

4 **1,2,3,4-tetra-O-acetyl-6,6-difluoro-D-rhamnopyranoside (P-D-Rha6F$_2$, 4)**. Synthesis was performed as described previously.[5] **TLC:** (50:50, EtOAc:Hept v/v) R_f = 0.55. **HRMS** *(m/z)*: $[M+Na]^+$ calcd for $C_{14}H_{18}F_2NaO_9$, 391.08166; found, 391.08289. **^{1}H NMR** (500 MHz, $CDCl_3$) δ 6.12 (d, *J* = 2.0 Hz, 1H, H1), 5.83 (td, *J* = 54.2, 3.1 Hz, 1H, H6), 5.53 (t, *J* = 10.0 Hz, 1H, H4), 5.37 (dd, *J* = 10.0, 3.5 Hz, 1H, H3), 5.26 (dd, *J* = 3.5, 2.0 Hz, 1H, H2), 4.04 (qd, *J* = 9.9, 3.1 Hz, 1H, H5), 2.19 (s, 3H, CH_3 Ac), 2.18 (s, 3H, CH_3 Ac), 2.06 (s, 3H, CH_3 Ac), 2.02 (s, 3H, CH_3 Ac). **^{13}C NMR** (126 MHz, $CDCl_3$) δ 170.04 (CO Ac), 169.80 (CO Ac), 169.42 (CO Ac), 167.93 (CO Ac), 113.62 (t, *J* = 245.6 Hz, C6), 90.27 (C1), 70.79 (t, *J* = 24.3 Hz, C5), 68.28 (C3), 68.02 (C2), 63.97 (dd, *J* = 3.7, 2.0 Hz, C4), 20.91 (CH_3 Ac), 20.82 (CH_3 Ac), 20.72 (CH_3 Ac), 20.67 (CH_3 Ac); **^{19}F NMR** (471 MHz, $CDCl_3$) δ -126.43 (ddd, *J* = 295.5, 53.8, 9.4 Hz, $6F_a$), -130.59 (ddd, *J* = 295.6, 54.5, 11.2 Hz, $6F_b$); **HRMS** *(m/z)*: $[M+Na]^+$ calcd for $C_{14}H_{18}F_2NaO_9$, 391.08166; found, 391.08289.

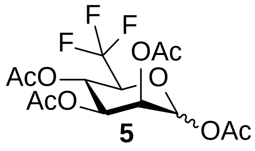

5 **1,2,3,4-tetra-O-acetyl-6,6,6-trifluoro-D-rhamnopyranoside (P-D-Rha6F$_3$, 5)**. Compound **21** (40 mg; 0.12 mmol) was dissolved in pyridine (190 µL; 20 eq.) and acetic anhydride

(110 µL; 10 eq.) was slowly added at 0℃. The mixture was then stirred for 4 hrs at r.t.. The mixture was concentrated *in vacuo* and the residue was dissolved in DCM, washed with aq. HCl (1 M) and sat. aq. $NaHCO_3$. The organic layer was dried over anhydrous $MgSO_4$, filtered and concentrated *in vacuo*. The residue was purified by silicagel flash column chromatography (0% → 30% EtOAc in Hept) to afford **5** (39.4 mg; 102 µmol; 88%). **TLC:** (50:50, EtOAc:Hept v/v) R_f = 0.46. **^{1}H-NMR** (500 MHz, $CDCl_3$) δ 6.17 (d, *J* = 2.0 Hz, 1H, H1), 5.59 (t, *J* = 10.0 Hz, 1H, H4), 5.37 (dd, *J* = 10.0, 3.4 Hz, 1H, H3), 5.26 (dd, *J* = 3.4, 2.1 Hz, 1H, H2), 4.22 (dt, *J* = 10.1, 5.6 Hz, 1H, H5), 2.20 (s, 3H, CH_3 Ac), 2.19 (s, 3H, CH_3 Ac), 2.06 (s, 3H, CH_3 Ac), 2.03 (s, 3H, CH_3 Ac). **^{13}C-NMR** (126 MHz, $CDCl_3$) δ 169.97 (CO Ac), 169.79 (CO Ac), 169.02 (CO Ac), 167.64 (CO Ac), 122.84 (q, *J* = 280.8 Hz, C6), 90.16 (C1), 70.22 (q, *J* = 31.5 Hz, C5), 68.09 (C3), 67.80 (C2), 63.71 (C4), 20.87 (CH_3 Ac), 20.81 (CH_3 Ac), 20.69 (CH_3 Ac), 20.58 (CH_3 Ac); **^{19}F NMR** (471 MHz, $CDCl_3$) δ -75.56 (d, *J* = 5.8 Hz, CF_3); **HRMS** *(m/z)*: $[M+Na]^+$ calcd for $C_{14}H_{17}F_3NaO_9$, 409.07224; found, 409.07328.

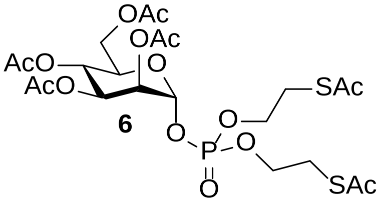

6

Di-(S-acetyl-2-thioethyl)-(2,3,4,6-tetra-O-acetyl-α-D-mannopyranoside) phosphate (P-D-Man-1P, 6). 2,3,4,6-tetra-O-acetyl-D-mannopyranoside[6] was reacted as described in general procedure B and purified by silicagel flash column chromatography (0% → 50% EtOAc in Hept) affording **6** (61.6 mg; 97.4 µmol; 42%). **TLC:** before oxidation (80:20, EtOAc:Hept v/v) R_f = 0.76 & after oxidation (80:20, EtOAc:Hept v/v) R_f = 0.51. **^{1}H NMR** (500 MHz, $CDCl_3$) δ 5.65 (dd, *J* = 6.5, 1.7 Hz, 1H, H1), 5.38 – 5.32 (m, 3H, H2 & H3 & H4), 4.31 (dd, *J* = 12.4, 4.7 Hz, 1H, H6a), 4.24 – 4.16 (m, 5H, H5 & 2x CH_2-O), 4.14 (dd, *J* = 12.4, 2.4 Hz, 1H, H6b), 3.20 (td, *J* = 6.4, 4.7 Hz, 4H, 2x CH_2-S), 2.37 (s, 6H, 2x CH_3 SAc), 2.18 (s, 3H, CH_3 OAc), 2.11 (s, 3H, CH_3 OAc), 2.06 (s, 3H, CH_3 OAc), 2.00 (s, 3H, CH_3 OAc); **^{13}C NMR** (126 MHz, $CDCl_3$) δ 194.72 (CO SAc), 194.70 (CO SAc), 170.64 (CO OAc), 169.87 (CO OAc), 169.68 (CO OAc), 169.66 (CO OAc), 95.44 (d, *J* = 5.4 Hz, C1), 70.59 (C5), 68.85 (d, *J* = 10.9 Hz, C2), 68.34 (C3), 66.83 – 66.59 (m, 2x CH_2-O), 65.41 (C4), 62.13 (C6), 30.66 (CH_3 SAc), 30.64 (CH_3 SAc), 29.22 (d, *J* = 7.3 Hz, 2x CH_2-S), 20.86 (CH_3 OAc), 20.83 (CH_3 OAc), 20.78 (CH_3 OAc), 20.72 (CH_3 OAc). **^{31}P NMR** (202 MHz, $CDCl_3$) δ -4.14 (1-OP); **HRMS** *(m/z)*: $[M+Na]^+$ calcd for $C_{22}H_{33}NaO_{15}PS_2$, 655.08962; found, 655.08718.

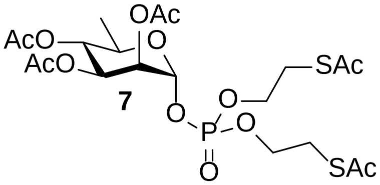

7

Di-(S-acetyl-2-thioethyl)-(2,3,4-tri-O-acetyl-α-D-rhamnopyranoside) phosphate (P-D-Rha-1P, 7). Compound **11** was reacted as described in general procedure B and purified by silicagel flash column chromatography (0% → 50% EtOAc in Hept) affording **7** (234 mg; 407 µmol; 43%). **TLC:** before oxidation (60:40, EtOAc:Hept v/v) R_f = 0.61 & after oxidation (60:40, EtOAc:Hept v/v) R_f = 0.25. **^{1}H NMR** (500 MHz, $CDCl_3$) δ 5.58 (dd, *J* = 6.3, 1.9 Hz, 1H, H1), 5.34 – 5.29 (m, 2H, H2 & H3), 5.11 (t, *J* = 10.0 Hz, 1H, H4), 4.23 – 4.05 (m, 5H, 2x CH_2-O & H5), 3.20 (td, *J* = 6.4, 4.6 Hz, 4H, 2x CH_2-S), 2.37 (s, 6H, 2x CH_3 SAc), 2.16 (s, 3H, CH_3 OAc), 2.07 (s, 3H, CH_3 OAc), 1.99 (s, 3H, CH_3 OAc), 1.26 (d, *J* = 6.2 Hz, 3H, 3x H6); **^{13}C NMR** (126 MHz, $CDCl_3$)

δ 194.79 (CO SAc), 194.76 (CO SAc), 169.97 (2x CO OAc), 169.78 (CO OAc), 95.56 (d, *J* = 5.3 Hz, C1), 70.35 (C4), 69.14 (d, *J* = 11.0 Hz, C2), 68.73 (C5), 68.38 (C3), 66.72 – 66.48 (m, 2x CH_2-O), 30.66 (2x CH_3 SAc), 29.25 (d, *J* = 7.4 Hz, 2x CH2-S), 20.88 (2x CH_3 OAc), 20.77 (CH_3 OAc), 17.48 (C6); ^{31}**P NMR** (202 MHz, $CDCl_3$) δ -3.97 (1-OP); **HRMS** *(m/z)*: $[M+Na]^+$ calcd for $C_{20}H_{31}NaO_{13}PS_2$, 597.08414; found, 597.08291.

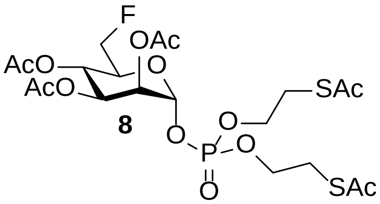

8

Di-(S-acetyl-2-thioethyl)-(2,3,4-tri-O-acetyl-6-fluoro-α-D-rhamnopyranoside) phosphate (P-D-Rha6F-1P, 8). Compound **12** was reacted as described in general procedure B and purified by silicagel flash column chromatography (0% → 60% EtOAc in Hept) affording **8** (10.7 mg; 18.0 µmol; 19%). **TLC:** before oxidation (60:40, EtOAc:Hept v/v) R_f = 0.61 & after oxidation (60:40, EtOAc:Hept v/v) R_f = 0.34. ^{1}**H NMR** (500 MHz, $CDCl_3$) δ 5.66 (dd, *J* = 6.5, 1.8 Hz, 1H, H1), 5.40 – 5.32 (m, 3H, H2 & H3 & H4), 4.58 – 4.42 (m, 2H, H6a & H6b), 4.24 – 4.15 (m, 5H, H5 & 2x CH_2-O), 3.23 – 3.17 (m, 4H, 2x CH_2-S), 2.37 (s, 3H, CH_3 SAc), 2.36 (s, 2H, CH_3 SAc), 2.17 (s, 3H, CH_3 OAc), 2.08 (s, 3H, CH_3 OAc), 2.01 (s, 3H, CH_3 OAc); ^{13}**C NMR** (126 MHz, $CDCl_3$) δ 194.79 (CO SAc), 194.78 (CO SAc), 169.92 (CO OAc), 169.75 (CO OAc), 169.65 (CO OAc), 95.39 (d, *J* = 5.4 Hz, C1), 81.21 (d, *J* = 176.0 Hz, C6), 71.31 (d, *J* = 19.2 Hz, C5), 68.79 (d, *J* = 11.2 Hz, C3), 68.31 (C2), 66.85 – 66.62 (m, 2x CH_2-O), 64.97 (d, *J* = 6.7 Hz, C4), 30.66 (2x CH_3 SAc), 29.27 – 29.15 (m, 2x CH_2-S), 20.86 (CH_3 Ac), 20.79 (CH_3 Ac), 20.74 (CH_3 Ac); ^{31}**P NMR** (202 MHz, $CDCl_3$) δ -4.05 (1-OP); ^{19}**F NMR** (470 MHz, $CDCl_3$) δ -232.24 (td, *J* = 47.2, 23.0 Hz, 6F); **HRMS** *(m/z)*: $[M+Na]^+$ calcd for $C_{20}H_{30}FNaO_{13}PS_2$, 615.07472; found, 615.07346.

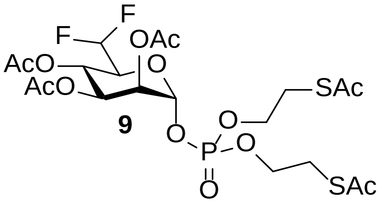

9

Di-(S-acetyl-2-thioethyl)-(2,3,4-tri-O-acetyl-6,6-difluoro-α-D-rhamnopyranoside) phosphate (P-D-Rha6F$_2$-1P, 9). Compound **13** was reacted as described in general procedure B and purified by silicagel flash column chromatography (0% → 50% EtOAc in Hept) affording **9** (50.0 mg; 81.9 µmol; 32%). **TLC:** before oxidation (60:40, EtOAc:Hept v/v) R_f = 0.65 & after oxidation (60:40, EtOAc:Hept v/v) R_f = 0.42; ^{1}**H NMR** (500 MHz, $CDCl_3$) δ 5.86 (td, *J* = 54.0, 3.1 Hz, 1H, H6), 5.68 (dd, *J* = 6.5, 2.0 Hz, 1H, H1), 5.50 (t, *J* = 10.0 Hz, 1H, H4), 5.38 (dd, *J* = 9.9, 3.4 Hz, 1H, H3), 5.34 (dd, *J* = 3.5, 2.0 Hz, 1H, H2), 4.20 (dddd, *J* = 14.4, 9.6, 5.5, 2.5 Hz, 5H, H5 & 2x CH_2-O), 3.20 (q, *J* = 6.3 Hz, 4H, 2x CH_2-S), 2.37 (s, 3H, CH_3 SAc), 2.37 (s, 3H, CH_3 SAc), 2.17 (s, 3H, CH_3 OAc), 2.07 (s, 3H, CH_3 OAc), 2.01 (s, 3H, CH_3 OAc); ^{13}**C NMR** (126 MHz, $CDCl_3$) δ 194.70 (CO SAc), 194.69 (CO SAc), 169.78 (CO OAc), 169.60 (CO OAc), 169.44 (CO OAc), 113.40 (t, *J* = 245.7 Hz, C6), 94.95 (d, *J* = 5.3 Hz, C1), 70.42 (t, *J* = 23.9 Hz, C5), 68.39 (d, *J* = 11.0 Hz, C2), 67.74 (C3), 66.90 – 66.69 (m, 2x CH_2-O), 63.87 (t, *J* = 2.8 Hz, C4), 30.60 (2x CH_3 SAc), 29.18 – 29.08 (m, 2x CH_2-S), 20.76 (CH_3 OAc), 20.65 (2x CH_3 OAc); ^{19}**F NMR** (471 MHz, $CDCl_3$) δ -127.31 (ddd, *J* = 295.3, 53.9,

10.3 Hz, 6F$_a$), -130.57 (ddd, *J* = 295.2, 54.3, 10.3 Hz, 6F$_b$); **^{31}P NMR** (202 MHz, $CDCl_3$) δ -4.05 (1OP); **HRMS** *(m/z)*: [M+Na]$^+$ calcd for $C_{20}H_{29}F_2NaO_{13}PS_2$, 633.06529; found, 633.06322.

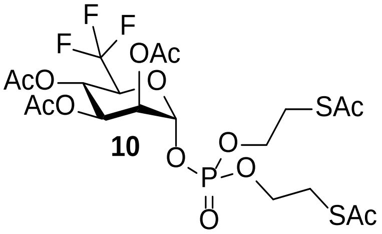

Di-(S-acetyl-2-thioethyl)-(2,3,4-tri-O-acetyl-6,6,6-trifluoro-α-D-mannopyranoside) phosphate (P-D-Rha6F$_3$-1P, 10). Compound **21** was reacted as described in general procedure B and purified by silicagel flash column chromatography (0% → 40% EtOAc in Hept) affording **10** (95.0 mg; 151 µmol; 35%). **TLC:** before oxidation (60:40, EtOAc:Hept v/v) R_f = 0.65 & after oxidation (60:40, EtOAc:Hept v/v) R_f = 0.39; **^{1}H-NMR** (500 MHz, $CDCl_3$) δ 5.71 (dd, *J* = 6.6, 1.9 Hz, 1H, H1), 5.58 (t, *J* = 9.9 Hz, 1H, H4), 5.38 (dd, *J* = 9.9, 3.4 Hz, 1H, H3), 5.35 (dd, *J* = 3.4, 2.0 Hz, 1H, H2), 4.41 (dq, *J* = 11.1, 5.6 Hz, 1H, H5), 4.27 – 4.14 (m, 4H, 2x CH_2-O), 3.22 – 3.17 (m, 4H, 2x CH_2-S), 2.37 (s, 3H, CH_3 SAc), 2.37 (s, 3H, CH_3 SAc), 2.19 (s, 3H, CH_3 OAc), 2.07 (s, 3H, CH_3 OAc), 2.02 (s, 3H, CH_3 OAc); **^{13}C-NMR** (126 MHz, $CDCl_3$) δ 194.68 (CO SAc), 194.65 (CO SAc), 169.71 (CO Ac), 169.60 (CO Ac), 169.03 (CO Ac), 122.84 (q, J = 280.8 Hz, C6), 94.79 (d, J = 5.3 Hz, C1), 69.81 (q, J = 31.6 Hz, C5), 68.16 (d, J = 11.0 Hz, C2), 67.54 (C3), 67.03 – 66.80 (m, 2xCH_2-O), 63.48 (C4), 30.61 (CH_3 SAc), 30.60 (CH_3 SAc), 29.19 – 29.05 (m, 2x CH_2-S), 20.75 (CH_3 OAc), 20.63 (CH_3 OAc), 20.55 (CH_3 OAc); **^{19}F NMR** (471 MHz, $CDCl_3$) δ -75.48 (d, *J* = 5.7 Hz, CF_3); **^{31}P NMR** (202 MHz, $CDCl_3$) δ -4.05 (1-OP); **HRMS** *(m/z)*: [M+Na]$^+$ calcd for $C_{20}H_{28}F_3NaO_{13}PS_2$, 651.05587; found, 651.05355.

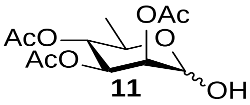

2,3,4-tri-O-acetyl-D-rhamnopyranoside (11). Compound **2** was reacted as described in general procedure A and purified by silicagel flash column chromatography (0% → 50% EtOAc in Hept) affording **11** (323 mg; 1.11 mmol; 83%). **TLC:** (50:50, EtOAc:Hept v/v) R_f = 0.33. **^{1}H NMR** (500 MHz, $CDCl_3$, major anomer) δ 5.37 (dd, *J* = 10.1, 3.4 Hz, 1H, H3), 5.27 (dd, *J* = 3.5, 1.8 Hz, 1H, H2), 5.17 – 5.15 (m, 1H, H1), 5.08 (t, *J* = 10.0 Hz, 1H, H4), 4.17 – 4.10 (m, 1H, H5), 3.44 (d, *J* = 3.7 Hz, 1H, 1OH), 2.16 (s, 3H, CH_3 Ac), 2.06 (s, 3H, CH_3 Ac), 2.00 (s, 3H, CH_3 Ac), 1.22 (d, *J* = 6.3 Hz, 3H, 3x H6). **^{13}C NMR** (126 MHz, $CDCl_3$, major anomer) δ 170.45 (CO Ac), 170.30 (CO Ac), 170.26 (CO Ac), 92.23 (C1), 71.27 (C4), 70.43 (C2), 68.97 (C3), 66.50 (C5), 21.05 (CH_3 Ac), 20.94 (CH_3 Ac), 20.86 (CH_3 Ac), 17.59 (C6). **HRMS** *(m/z)*: [M+Na]$^+$ calcd for $C_{12}H_{18}NaO_8$, 313.08994; found, 313.09287.

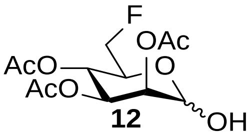

2,3,4-tri-O-acetyl-6-fluoro-D-rhamnopyranoside (12). Compound **3** was reacted as described in general procedure A and purified by silicagel flash column chromatography (0% → 35% EtOAc in Hept) affording **12** (29.0 mg; 94.1 µmol; 66%). **TLC:** (60:40, EtOAc:Hept v/v) R_f = 0.41. **^{1}H NMR** (500 MHz, $CDCl_3$, major anomer) δ 5.44 (dd, *J* = 10.0, 3.3 Hz, 1H, H3), 5.31 – 5.27 (m, 2H, H4 & H2), 5.25 (d, *J* = 1.8 Hz, 1H, H1), 4.54 – 4.42 (m, 2H, H6a & H6b), 4.24 (dddd, *J* = 22.9,

10.3, 4.4, 2.8 Hz, 1H, H5), 3.96 (s, 1H, 1-OH), 2.16 (s, 3H, CH_3 Ac), 2.07 (s, 3H, CH_3 Ac), 2.01 (s, 3H, CH_3 Ac); **^{13}C NMR** (126 MHz, $CDCl_3$, major anomer) δ 170.47 (CO Ac), 170.30 (CO Ac), 170.03 (CO Ac), 92.24 (C1), 81.85 (d, *J* = 174.2 Hz, C6), 70.11 (C2), 69.29 (d, *J* = 19.0 Hz, C5), 68.90 (C3), 65.85 (d, *J* = 7.0 Hz, C4), 21.00 (CH_3 Ac), 20.81 (2x CH_3 Ac); **^{19}F NMR** (470 MHz, $CDCl_3$, major anomer) δ -231.87 (td, *J* = 47.2, 22.9 Hz, 6F); **HRMS** *(m/z)*: $[M+Na]^+$ calcd for $C_{12}H_{17}FNaO_8$, 331.08051; found, 331.08349.

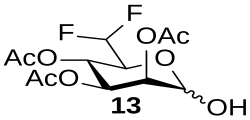

2,3,4-tri-O-acetyl-6,6-difluoro-D-rhamnopyranoside (13). Compound **4** was reacted as described in general procedure A and purified by silicagel flash column chromatography (0% → 40% EtOAc in Hept) affording **13** (84.0 mg; 257 µmol; 83%). **TLC:** (60:40, EtOAc:Hept v/v) R_f = 0.51. **^{1}H-NMR** (500 MHz, $CDCl_3$, major anomer) δ 5.83 (td, *J* = 54.3, 3.2 Hz, 1H, H6), 5.47 – 5.40 (m, 2H, H4 & H3), 5.28 – 5.24 (m, 2H, H1 & H2), 4.65 (s, 1H, 1-OH), 4.24 (ddq, *J* = 13.3, 6.3, 3.3 Hz, 1H, H5), 2.16 (s, 3H, CH_3 Ac), 2.06 (s, 3H, CH_3 Ac), 2.02 (s, 3H, CH_3 Ac); **^{13}C-NMR** (126 MHz, $CDCl_3$, major anomer) δ 170.55 (CO Ac), 170.43 (CO Ac), 170.04 (CO Ac), 114.05 (t, *J* = 244.8 Hz, C6), 92.13 (C1), 69.85 (C2), 68.92 – 68.47 (m, C5 & C3), 64.84 (t, *J* = 3.1 Hz, C4), 20.91 (CH_3 Ac), 20.74 (CH_3 Ac), 20.69 (CH_3 Ac); **^{19}F NMR** (471 MHz, $CDCl_3$, major anomer) δ -127.38 (ddd, *J* = 293.8, 54.4, 11.4 Hz, $6F_a$), -130.25 (ddd, *J* = 293.7, 54.3, 10.1 Hz, $6F_b$). **HRMS** *(m/z)*: $[M+Na]^+$ calcd for $C_{12}H_{16}F_2NaO_8$, 349.07109; found, 349.07331.

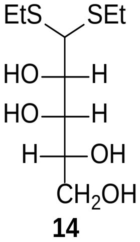

D-lyxose diethyl dithioacetal (14). D-Lyxose (6.00 g; 40.0 mmol) was dissolved in conc. HCl (27 mL, 1.5 M) and ethanethiol (6.3 mL; 81.9 mmol; 2.05 eq.; 97% wt.) was added dropwise at 0 ℃. After stirring for 27 hrs at 0 ℃ the mixture was extracted with EtOAc (4x) and the combined organic phases were neutralized by portion wise addition of solid $NaHCO_3$, dried over Na_2SO_4, filtered and concentrated *in vacuo* to afford **14** as white crystals (9.90 g; 38.6 mmol; 97%). **TLC:** (20:80, MeOH:DCM v/v) R_f = 0.60. **^{1}H NMR** (500 MHz, $(CD_3)_2SO$)) δ 4.98 (d, *J* = 6.1 Hz, 1H, 2-OH), 4.46 (t, *J* = 5.6 Hz, 1H, 5-OH), 4.23 (d, *J* = 8.2 Hz, 1H, 3-OH), 4.21 (d, *J* = 1.5 Hz, 1H, H1), 4.17 (d, *J* = 6.7 Hz, 1H, 4-OH), 3.84 (ddd, *J* = 9.3, 6.1, 1.6 Hz, 1H, H2), 3.71 (d, *J* = 6.8 Hz, 1H, H4), 3.54 (t, *J* = 8.5 Hz, 1H, H3), 3.44 – 3.35 (m, 2H, 2x H5), 2.68 – 2.59 (m, 4H, 2x CH_2 SEt), 1.19 (dt, *J* = 8.7, 7.4 Hz, 6H, 2x CH_3 SEt); **^{13}C NMR** (126 MHz, $(CD_3)_2SO$)) δ 73.85 (C2), 70.34 (C3), 69.56 (C4), 62.94 (C5), 54.98 (C1), 24.84 (CH_2 SEt), 24.67 (CH_2 SEt), 14.74 (CH_3 SEt), 14.71 (CH_3 SEt); **HRMS** *(m/z)*: $[M+Na]^+$ calcd for $C_9H_{20}NaO_4S_2$, 279.07007; found, 279.07299.

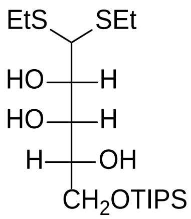

15

5-O-triisopropylsilyl-D-lyxose diethyl dithioacetal (15). Compound **14** (9.90 g; 38.6 mmol) and 4-dimethylaminopyridine (472 mg; 3.86 mmol; 0.1 eq.) were dissolved in pyridine (110 mL; 0.35 M) and subsequently triisopropylsilylchloride (9.3 mL; 42.5 mmol; 1.1 eq.; 97% wt.) was added dropwise at 0℃. After 48 hrs most of the starting material had converted and the mixture was concentrated in vacuo. The residue was dissolved in EtOAc, washed with water, dried over Na_2SO_4, filtered and concentrated *in vacuo*. The residue was used for the next reaction without further purification. **TLC:** (50:50, EtOAc:Hept v/v) R_f = 0.69; ^{1}**H NMR** (500 MHz, $CDCl_3$) δ 4.23 (d, *J* = 2.9 Hz, 1H, H1), 4.04 (q, *J* = 5.3 Hz, 1H, H4), 3.97 – 3.88 (m, 4H, H2 & H3 & H5a & H5b), 3.12 (d, *J* = 5.3 Hz, 1H, 3-OH), 2.90 (d, *J* = 5.8 Hz, 1H, 4-OH), 2.87 (d, *J* = 4.0 Hz, 1H, 2-OH), 2.74 (q, *J* = 7.4 Hz, 2H, CH_2 SEt), 2.67 (q, *J* = 7.4 Hz, 2H, CH_2 SEt), 1.29 (td, *J* = 7.4, 3.8 Hz, 6H, 2x CH_3 SEt), 1.18 – 1.10 (m, 3H, 3x CH TIPS), 1.08 (d, *J* = 6.5 Hz, 18H, 3x CH_3 TIPS); ^{13}**C NMR** (126 MHz, $CDCl_3$) δ 73.30 (C2), 72.20 (C3), 69.67 (C4), 66.75 (C5), 54.84 (C1), 25.94 (CH_2 SEt), 25.82 (CH_2 SEt), 18.06 (3x CH_3 TIPS), 18.05 (3x CH_3 TIPS), 14.85 (CH_3 SEt), 14.69 (CH_3 SEt), 11.93 (3x CH TIPS). **HRMS** *(m/z)*: $[M+Na]^+$ calcd for $C_{18}H_{40}NaO_4S_2Si$, 435.20350; found, 435.20401.

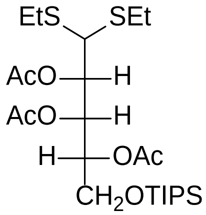

16

2,3,4-tri-O-acetyl-5-O-triisopropylsilyl-D-lyxose diethyl dithioacetal (16). Crude **15** was dissolved in pyridine (190 mL; 60 eq.) and acetic anhydride (109 mL; 30 eq.) was slowly added at 0℃. The mixture was stirred for 28 hrs while slowly warming to r.t.. The mixture was concentrated *in vacuo* and the residue was dissolved in DCM, washed with aq. HCl (1 M), sat. aq. NaHCO3 and brine. The organic layer was dried over anhydrous $MgSO_4$, filtered and concentrated *in vacuo*. The residue was purified by silicagel flash column chromatography (0% → 10% EtOAc in Hept) to afford **16** (12.09 g; 22.444 mmol; 58% over 2 steps). **TLC:** (10:90, EtOAc:Hept v/v) R_f = 0.24; ^{1}**H NMR** (500 MHz, $CDCl_3$) δ 5.76 (dd, *J* = 6.9, 1.8 Hz, 1H, H3), 5.39 – 5.33 (m, 2H, H2 & H4), 3.96 (d, *J* = 5.4 Hz, 1H, H1), 3.74 – 3.64 (m, 2H, 2x H5), 2.76 – 2.59 (m, 4H, 2x CH_2 Et), 2.10 (s, 3H, CH_3 Ac), 2.08 (s, 3H, CH_3 Ac), 2.07 (s, 3H, CH_3 Ac), 1.25 (dt, *J* = 8.4, 7.4 Hz, 6H, 2x CH_3 Et), 1.10 – 1.02 (m, 21H, 6x CH_3 TIPS & 3x CH TIPS); ^{13}**C NMR** (126 MHz, $CDCl_3$) δ 170.36 (CO Ac), 169.87 (CO Ac), 169.74 (CO Ac), 71.65 (C2), 70.74 (C4), 70.26 (C3), 61.85 (C5), 51.76 (C1), 25.58 (CH_2 Et), 25.16 (CH_2 Et), 21.17 (CH_3 Ac), 20.92 (2x CH_3 Ac), 17.99 (6x CH_3 TIPS), 14.40 (CH_3 Et), 14.16 (CH_3 Et), 11.99 (3x CH TIPS); **HRMS** *(m/z)*: $[M+Na]^+$ calcd for $C_{24}H_{46}NaO_7S_2Si$, 561.23519; found, 561.23388.

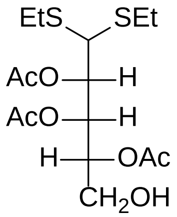

17

2,3,4-tri-O-acetyl-D-lyxose diethyl dithioacetal (17). Compound **16** (12.00 g; 22.27 mmol) was dissolved in THF (150 mL; 0.15 M) in a plastic bottle and hydrogen fluoride pyridine complex (15 mL; ~70%/30%) was added in portions over 3.5 hrs at 0 ℃. After stirring for an additional 14 hrs at r.t. all starting material was consumed and the mixture was diluted with EtOAc (100 mL) and carefully quenched with sat. aq. $NaHCO_3$. The organic layer was washed with 10% aq. $CuSO_4$ (2x) and the combined organic layers were dried over anhydrous $MgSO_4$, filtered and concentrated *in vacuo*. The residue was purified by silicagel flash column chromatography (0% → 40% EtOAc in Hept) to afford **17** (4.85 g; 12.7 mmol; 57%). **TLC:** (40:60, EtOAc:Hept v/v) R_f = 0.18. **^{1}H NMR** (400 MHz, $CDCl_3$) δ 5.59 (dd, J = 8.8, 1.7 Hz, 1H, H3), 5.39 (dd, J = 8.8, 3.5 Hz, 1H, H2), 5.06 (ddd, J = 7.8, 6.2, 1.7 Hz, 1H, H4), 3.86 (d, J = 3.5 Hz, 1H, H1), 3.53 (dt, J = 12.0, 5.9 Hz, 1H, H5a), 3.38 (dd, J = 11.7, 7.8 Hz, 1H, H5b), 2.80 (d, J = 7.1 Hz, 1H, 5-OH), 2.71 – 2.52 (m, 4H, 2x CH_2 SEt), 2.11 (s, 3H, CH_3 Ac), 2.02 (s, 3H, CH_3 Ac), 2.01 (s, 3H, CH_3 Ac), 1.18 (td, J = 7.4, 5.9 Hz, 6H, 2x CH_3 SEt); **^{13}C-NMR** (101 MHz, $CDCl_3$) δ 171.15 (CO Ac), 170.54 (CO Ac), 169.64 (CO Ac), 70.86 (C2), 70.62 (C4), 70.45 (C3), 60.05 (C5), 51.56 (C1), 25.81 (CH_2 SEt), 25.36 (CH_2 SEt), 20.93 (CH_3 Ac), 20.90 (CH_3 Ac), 20.70 (CH_3 Ac), 14.36 (CH_3 SEt), 14.15 (CH_3 SEt); **HRMS** *(m/z)*: $[M+Na]^+$ calcd for $C_{15}H_{26}NaO_7S_2$, 405.10176; found, 405.10253.

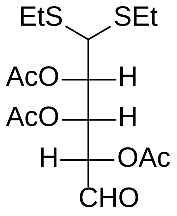

18

2,3,4-tri-O-acetyl-5-al-D-lyxose diethyl dithioacetal (18). Compound **17** (4.85 g; 12.7 mmol) was dissolved in dry DCM (106 mL; 0.12 M) and subsequently $NaHCO_3$ (10.7 g; 127 mmol; 10 eq.) and Dess-Martin periodinane (8.07 g; 19.0 mmol; 1.5 eq.) were added at 0 ℃. After 10 minutes the ice bath was removed and the mixture was stirred for an additional 3 hrs at r.t.. The mixture was diluted with DCM (100 mL) and an aqueous solution (150 mL) of Na_2SO_3 (15 g) and $NaHCO_3$ (5 g) was added at 0 ℃ and stirred for 30 min. The organic phase was separated, dried over anhydrous $MgSO_4$, filtered and concentrated *in vacuo*. The residue was purified by silicagel flash column chromatography (0% → 60% EtOAc in Hept) to afford **18** (3.65 g; 9.59 mmol; 76%) which was immediately used for the next step. **TLC:** (50:50, EtOAc:Hept v/v) R_f = 0.32. **HRMS** *(m/z)*: $[M+MeOH+Na]^+$ calcd for $C_{16}H_{28}NaO_8S_2$, 435.11233; found, 435.11239

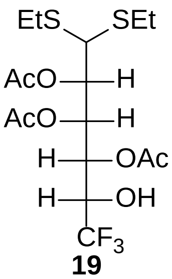

19

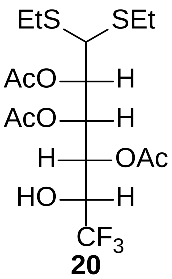

20

2,3,4-tri-O-acetyl-6,6,6-trifluoro-D-rhamnose diethyl dithioacetal (19) & 2,3,4-tri-O-acetyl-6,6,6-trifluoro-L-gulose diethyl dithioacetal (20). Synthetic procedure was adapted from a previously described procedure.[7] Compound **18** and trifluoromethyl(trimethyl)silane (1.45 mL; 9.56 mmol; 1.6 eq; 97% wt.) were dissolved in dry THF (20 mL; 0.3 M) and a tetrabutylammonium fluoride solution (598 µL; 598 µmol; 0.1 eq.; 1.0 M in THF) was added dropwise at 0℃. After stirring at 0℃ for 1.5 hrs the mixture was concentrated *in vacuo*. The residue was dissolved in $CHCl_3$, washed with water, dried over anhydrous Na_2SO_4, filtered and concentrated *in vacuo*. To remove the 5-OTMS groups, the residue was dissolved in 80% AcOH (0.25 M; 24 mL) and kept for 3 hrs at 50℃. The mixture was concentrated *in vacuo* and the residue was purified by silicagel flash column chromatography (0% → 20% → 30% EtOAc in Hept) to afford first **19** (813 mg; 1.80 mmol; 30%) and then **20** (816; 1.81 mmol; 30%). Compound **19**: **TLC:** (30:70, EtOAc:Hept v/v) R_f = 0.23. **^{1}H-NMR (**500 MHz, $CDCl_3$) δ 5.82 (dd, *J* = 8.4, 1.3 Hz, 1H, H3), 5.48 (dd, *J* = 9.5, 1.3 Hz, 1H, H4), 5.33 (dd, *J* = 8.4, 4.2 Hz, 1H, H2), 3.97 (d, *J* = 4.2 Hz, 1H, H1), 3.81 (dq, *J* = 9.4, 6.3 Hz, 1H, H5), 2.77 – 2.60 (m, 5H, OH & 2x CH_2 SEt), 2.22 (s, 3H, CH_3 Ac), 2.10 (s, 3H, CH_3 Ac), 2.07 (s, 3H, CH_3 Ac), 1.26 (q, *J* = 7.3 Hz, 7H, 2x CH_3 SEt); **^{13}C-NMR** (126 MHz, $CDCl_3$) δ 172.28 (CO), 169.85 (CO), 169.47 (CO), 124.34 (q, *J* = 282.2 Hz, C6), 71.14 (C3), 71.06 (C2), 68.05 – 67.27 (m, C5&C4), 51.67 (C1), 26.06 (CH_2 SEt), 25.68 (CH_2 SEt), 21.22 (CH_3 Ac), 20.95 (CH_3 Ac), 20.68 (CH_3 Ac), 14.40 (CH_3 SEt), 14.26 (CH_3 SEt). **^{19}F NMR** (471 MHz, $CDCl_3$) δ -75.75 (d, J = 6.4 Hz, CF_3); **HRMS** *(m/z)*: $[M+Na]^+$ calcd for $C_{16}H_{25}F_3NaO_7S_2$, 473.08915; found, 473.08916. Compound **20**: **TLC:** (30:70, EtOAc:Hept v/v) R_f = 0.18. **^{1}H-NMR** (500 MHz, $CDCl_3$) δ 5.79 (dd, *J* = 8.0, 2.5 Hz, 1H, H3), 5.60 (dd, *J* = 2.5, 1.4 Hz, 1H, H4), 5.33 (dd, *J* = 8.0, 4.2 Hz, 1H, H2), 4.29 – 4.21 (m, 1H, H5), 3.93 (d, *J* = 4.2 Hz, 1H, H1), 2.79 (d, *J* = 9.8 Hz, 1H, OH), 2.74 – 2.61 (m, 4H, 2x CH_2 SEt), 2.13 (s, 3H, CH_3 Ac), 2.13 (s, 3H, CH_3 Ac), 2.10 (s, 3H, CH_3 Ac), 1.25 (q, *J* = 7.5 Hz, 7H, 2x CH_3 SEt); **^{13}C-NMR** (126 MHz, $CDCl_3$) δ 170.13 (CO), 169.94 (CO), 169.14 (CO), 123.68 (q, *J* = 283.1 Hz, C6), 72.06 (C3), 71.20 (C2), 70.38 (q, *J* = 31.4 Hz, C5), 65.43 (d, *J* = 1.9 Hz, C4), 51.40 (C1), 25.86 (CH_2 SEt), 25.68 (CH_2 SEt), 21.07 (CH_3 Ac), 20.93 (CH_3 Ac), 20.68 (CH_3 Ac), 14.41 (CH_3 SEt), 14.21 (CH_3 SEt); **^{19}F NMR** (471 MHz, $CDCl_3$) δ -78.18 (d, *J* = 6.8 Hz, CF_3); **HRMS** *(m/z)*: $[M+Na]^+$ calcd for $C_{16}H_{25}F_3NaO_7S_2$, 473.08915; found, 473.08860.

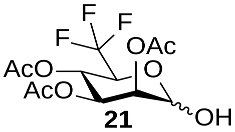

21

2,3,4-tri-O-acetyl-6,6,6-trifluoro-D-rhamnopyranoside (21). Compound **19** (763 mg; 1.69 mmol) was dissolved in a 5:1 acetone/water mixture (34 mL; 0.05 M) and NBS (1.51 g; 8.47 mmol; 5 eq.) was added at 0℃. After stirring for 45 min at 0℃, subsequently sat. aq. $NaHCO_3$ (15 mL) and sat. aq. $Na_2S_2O_3$ (15 mL) were added and the mixture was stirred for 15 min. The organic solvent was evaporated in vacuo and the remaining mixture was extracted with Et_2O (2x), dried over anhydrous

$MgSO_4$, filtered and concentrated *in vacuo*. The residue was purified by silicagel flash column chromatography (0% → 30% EtOAc in Hept) to afford **21** (225 mg; 654 µmol; 39%). **TLC:** (50:50, EtOAc:Hept v/v) R_f = 0.40; **^{1}H-NMR** (500 MHz, $CDCl_3$) δ 5.54 (t, *J* = 9.9 Hz, 1H, H4), 5.44 (dd, *J* = 10.0, 3.3 Hz, 1H, H3), 5.32 (s, 1H, H1), 5.26 (dd, *J* = 3.3, 1.9 Hz, 1H, H2), 4.42 (dq, *J* = 9.9, 5.9 Hz, 1H, H5), 4.20 (s, 1H, OH), 2.17 (s, 3H, CH_3 Ac), 2.06 (s, 3H, CH_3 Ac), 2.02 (s, 3H, CH_3 Ac); **^{13}C-NMR** (126 MHz, $CDCl_3$) δ 170.50 (CO Ac), 170.31 (CO Ac), 169.54 (CO Ac), 123.47 (q, *J* = 280.4 Hz, C6), 92.26 (C1), 69.56 (C2), 68.71 – 67.90 (m, C5 & C3), 64.32 (C4), 20.93 (CH_3 Ac), 20.74 (CH_3 Ac), 20.62 (CH_3 Ac); **^{19}F NMR** (471 MHz, $CDCl_3$) δ -75.44 (d, J = 6.0 Hz, 3x 6F); **HRMS** *(m/z)*: $[M+Na]^+$ calcd for $C_{12}H_{15}F_3NaO_8$, 367.06167; found, 367.06424.

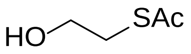

22

S-acetyl-2-thioethyl alcohol (22). Synthesis was performed as described previously and the data was identical.[8] **TLC:** (5:95, EtOAc:DCM v/v) R_f = 0.15. **^{1}H NMR** (500 MHz, $CDCl_3$) δ 3.67 (t, *J* = 6.3 Hz, 2H, CH_2-O), 3.01 (t, *J* = 6.3 Hz, 2H, CH_2-S), 2.30 (s, 3H, CH_3 Ac); **^{13}C NMR** (126 MHz, $CDCl_3$) δ 196.42 (CO), 61.48 (CH_2OH), 31.89 (CH_2-S), 30.60 (CH_3 Ac)

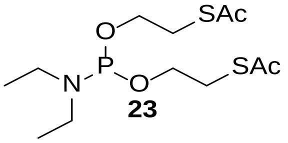

23

Di-(S-acetyl-2-thioethyl)-*N*,*N*-diethylphosphoroamidite (23). Compound **22** (5.88 g; 48.9 mmol; 2 eq.) was dissolved in anhydrous THF (31 mL; 1.6 M) and subsequently molecular sieves (4Å) and anhydrous TEA (6.1 mL; 44.0 mmol; 1.8 eq.) were added. The mixture was cooled to 0° C, diethylphosphoramidous dichloride (3.56 mL; 24.5 mmol; 1 eq.). After stirring for 2 hrs the mixture was diluted with heptane, filtered and the filtrate was concentrated and directly purified by silicagel flash column chromatography (3% TEA in Hept) to afford **23** (3.90 g; 11.4 mmol; 47%). The dry phosphoroamidite reagent was stored at -80℃ and after 11 months no changes in ^{1}H NMR were observed. **TLC:** (30:67:3, EtOAc:Hept:TEA v/v) R_f = 0.53. **^{1}H NMR** (500 MHz, $CDCl_3$) δ 3.73 (dddt, *J* = 32.3, 10.7, 8.4, 6.5 Hz, 4H), 3.11 (t, *J* = 6.6 Hz, 4H), 3.06 (dq, *J* = 9.5, 7.1 Hz, 4H), 2.34 (s, 6H), 1.06 (t, *J* = 7.1 Hz, 6H); **^{13}C NMR** (126 MHz, $CDCl_3$) δ 195.08, 61.76 (d, *J* = 16.0 Hz), 37.31 (d, *J* = 20.7 Hz), 30.49, 30.37 (d, *J* = 6.5 Hz), 14.98 (d, *J* = 3.2 Hz); **^{31}P NMR** (202 MHz, $CDCl_3$) δ 147.22 ($PNEt_2(OCH_2)_2$); **HRMS** *(m/z)*: $[M+Na]^+$ calcd for $C_{12}H_{24}NNaO_5PS_2$, 380.07312; found, 380.07340.

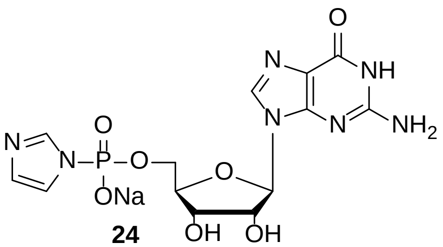

24

GMP-imidazole (24). Adapted from Vincent *et al*[9], the guanosine mono phosphate (GMP) triethylammonium salt was obtained by suspending GMP (free acid) in a water/ethanol mixture and neutralizing to pH 7 by a stepwise addition of triethylamine under vigorous stirring. The residue was lyophilized to obtain the GMP triethylammonium salt. To the GMP triethylammonium salt (837 mg; 1.80 mmol; 1 eq.), 2,2'-dithiopyridine (1.19 g; 5.41 mmol; 3 eq.), and imidazole (1.23 g; 18.0 mmol; 10 eq.) in anhydrous DMF (5.15 mL; 0.35 M), was added triethylamine (1.00 mL; 7.21 mmol; 4 eq.) and triphenylphosphine (1.42 g; 5.41 mmol; 3 eq.) at room temperature under argon. The reaction was stirred for 72 hours. The product was precipitated from the reaction mixture by the addition of an anhydrous solution of NaClO4 (1.77 g; 14.4 mmol; 8 eq.) in dry acetone (100 mL). The

heterogeneous mixture was cooled at 0 °C under argon. The precipitate was filtered, washed repeatedly with cold, dry acetone, and dried in vacuo to afford **24** with minor unknown impurities (944.5 mg) as an off-white solid. **TLC:** (2:1:1, n-butanol:AcOH:H_2O v/v) R_f = 0.31. **^{1}H NMR** (400 MHz, DMSO-d6) δ 10.69 (s, 1H, NH), 7.63 (s, 1H, CH Im & N-CH-N), 7.09 (s, 1H, CH Im), 6.85 (s, 1H, CH Im), 6.57 (s, 2H, NH_2), 5.67 (t, J = 8.6 Hz, 1H, H1), 4.55 – 4.49 (m, 1H, H2), 3.96 (d, J = 4.7 Hz, 1H, H3), 3.88 (s, 1H, H4), 3.81 – 3.62 (m, 1H, 2x H5); **^{13}C NMR** (101 MHz, DMSO-d6) δ 156.83 (CO), 153.66 (C-NH_2), 151.48 (N-C-N Gua), 139.19 (N-CH-N Gua), 132.05 (d, J = 2.8 Hz, CH Im), 128.71 (CH Im), 119.91 (d, J = 5.1 Hz, CH Im), 116.68 (C-C=O), 86.35 (C1), 83.67 (d, J = 8.4 Hz, C4), 73.21 (C2), 70.94 (C3), 64.88 (d, J = 5.6 Hz, C5); **^{31}P NMR** (162 MHz, DMSO-d6) δ -10.30 (5-OP).

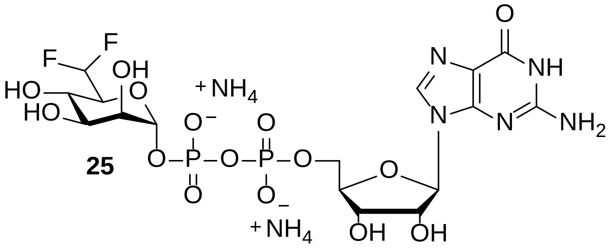

25

GDP-Rha6F_2 ammonium salt (25). Compound **9** (12.7 mg; 45.2 µmol; 1 eq.) was dissolved in anhydrous MeOH (904 µL; 50 mM) and 5.4 M NaOMe in MeOH (19.3 µL; 104 µmol; 2.3 eq.) was added at 0℃ after which the reaction was slowly warmed to room temperature. The reaction was stirred for 2 days and purified by size exclusion chromatography (50 mM ammonium bicarbonate in milli-Q). The fractions containing Rha6F_2-1P were lyophilized, transformed into the corresponding triethylammonium salt by DOWEX resins and lyophilized again. The dry phosphate triethylammonium salt of Rha6F_2-1P was dissolved in anhydrous DMF (451 µL; 0.1 M) and $MgCl_2$ (8.6 mg; 90 µmol; 2 eq.) was added. When a clear solution was obtained, GMP-imidazole (**24**) (28 mg; 68 µmol; 1.5 eq.) was added and stirred for 48 hrs. The reaction was quenched by the addition of water and lyophilized. The crude products were purified by preparative HPLC (stationary phase: VP150/21 NUCLEODUR HILIC, 5 um, Macherey-Nagel. Mobile phase: isocratic acetonitrile: 40 mM ammonium acetate in MilliQ water 75:25 v/v. Runtime: 55 min) and the product containing fractions were lyophilized. The dry product was then purified by size exclusion chromatography (P2, 50 mM ammonium bicarbonate in milli-Q) and the product containing fractions were lyophilized to afford **25** (3.9 mg; 45 µmol; 13%). **TLC:** (2:1:1, n-butanol:AcOH:H_2O v/v) R_f = 0.18. **^{1}H NMR** (500 MHz, D_2O) δ 8.12 (s, 1H, N=CH-N), 6.15 (td, J = 53.5, 1.4 Hz, 1H, H6'), 5.96 (d, J = 6.2 Hz, 1H, H1), 5.57 (dd, J = 7.8, 1.9 Hz, 1H, H1'), 4.79 (s, 206H, H2 & solvent peak), 4.52 (dd, J = 5.2, 3.4 Hz, 1H, H3), 4.36 (td, J = 3.5, 2.0 Hz, 1H, H4), 4.22 (dd, J = 5.5, 3.9 Hz, 2H, 2x H5), 4.11 – 4.05 (m, 2H, H5' & H2'), 3.97 (dd, J = 9.8, 3.3 Hz, 1H, H3'), 3.88 (t, J = 9.9 Hz, 1H, H4'); **^{13}C NMR** (126 MHz, D_2O) δ 160.06 (C=O), 154.75 (C-NH_2), 151.81 (N-C-N), 137.23 (N-CH-N), 116.40 (C-C=O), 114.18 (t, *J* = 242.2 Hz, C6'), 96.39 (d, *J* = 5.9 Hz, C1'), 86.76 (C1), 83.74 (C4), 73.67 (C2), 71.39 (d, *J* = 20.4 Hz, C5'), 70.41 (C3), 69.79 (d, *J* = 9.0 Hz, C2'), 69.34 (C3'), 65.37 (C4'), 65.29 (d, *J* = 5.6 Hz, C5); **^{31}P NMR** (202 MHz, D_2O) δ -11.54 (d, J = 18.9 Hz), -14.23 (d, J = 19.9 Hz); **^{19}F NMR** (471 MHz, D_2O) δ -132.22 (d, J = 12.6 Hz, $6F_a$), -132.33 (d, J = 13.3 Hz, $6F_b$).

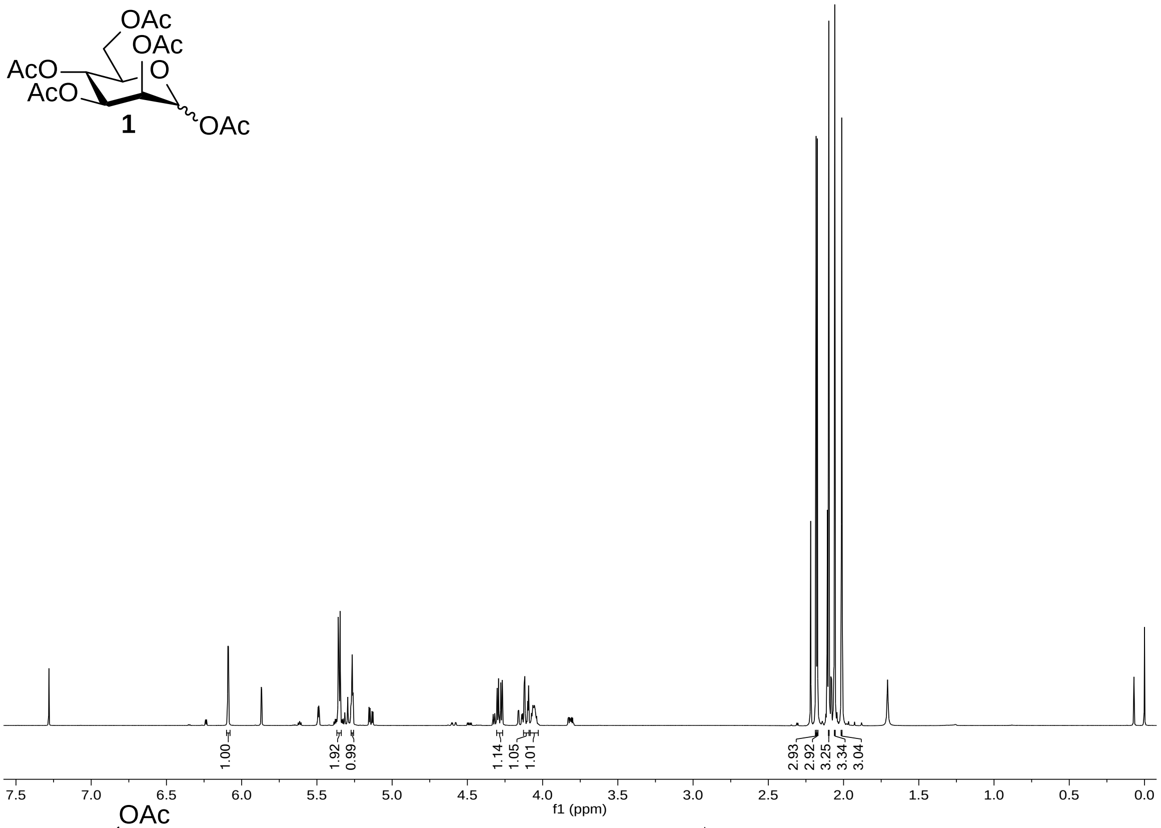
OAc
OAc
AcO
AcO
O
1
OAc
1.00
1.92
0.99
1.14
1.05
1.01
2.93
2.92
3.25
3.34
3.04
7.5 7.0 6.5 6.0 5.5 5.0 4.5 4.0 3.5 3.0 2.5 2.0 1.5 1.0 0.5 0.0
f1 (ppm)

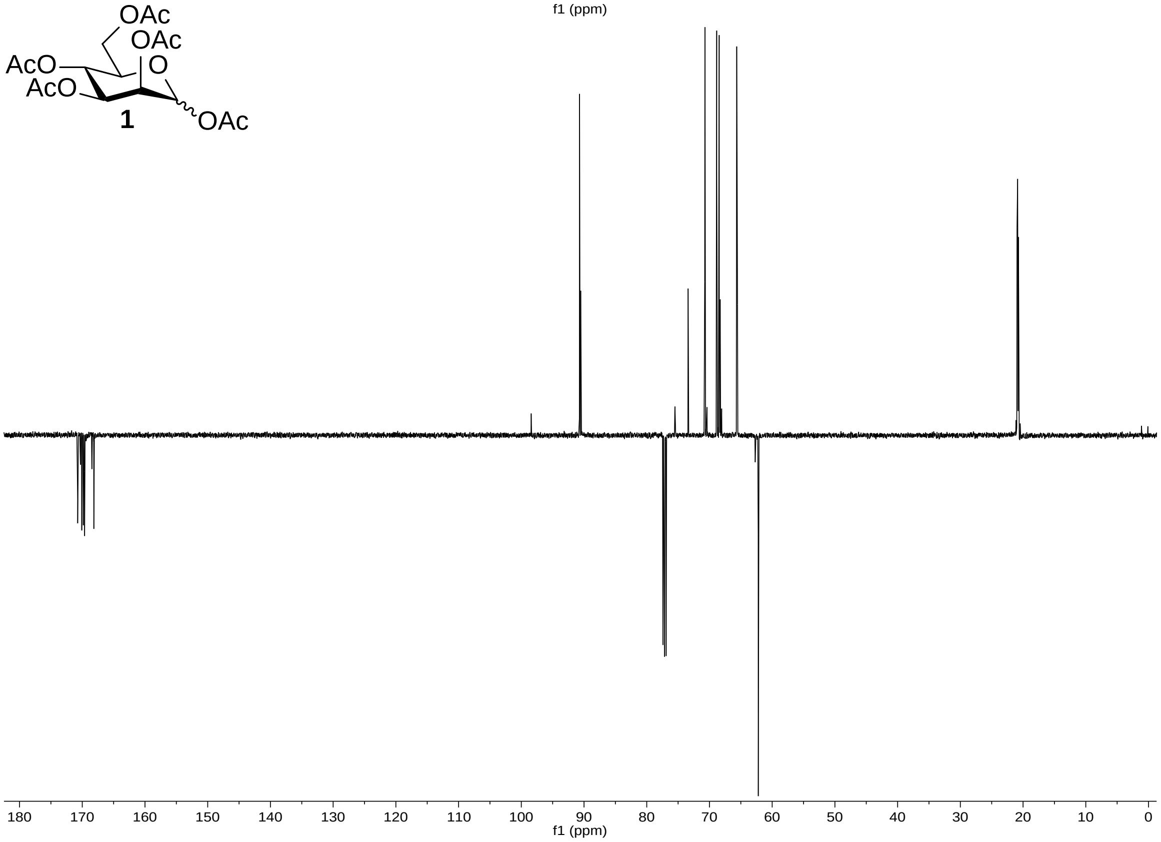
OAc
OAc
AcO
AcO
O
1
OAc
180 170 160 150 140 130 120 110 100 90 80 70 60 50 40 30 20 10 0
f1 (ppm)

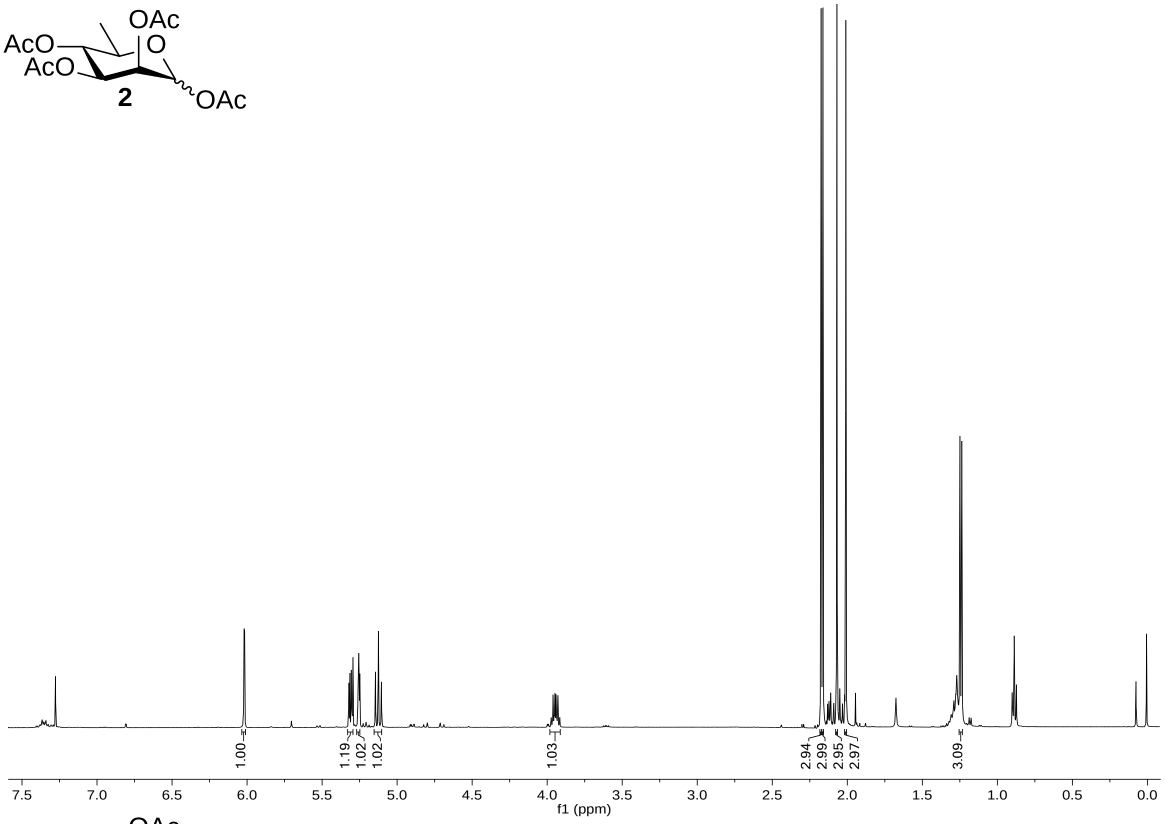
AcO
AcO
OAc
O
2
OAc
1.00
1.19
1.02
1.02
1.03
2.94
2.99
2.95
2.97
3.09
7.5
7.0
6.5
6.0
5.5
5.0
4.5
4.0
3.5
3.0
2.5
2.0
1.5
1.0
0.5
0.0
f1 (ppm)

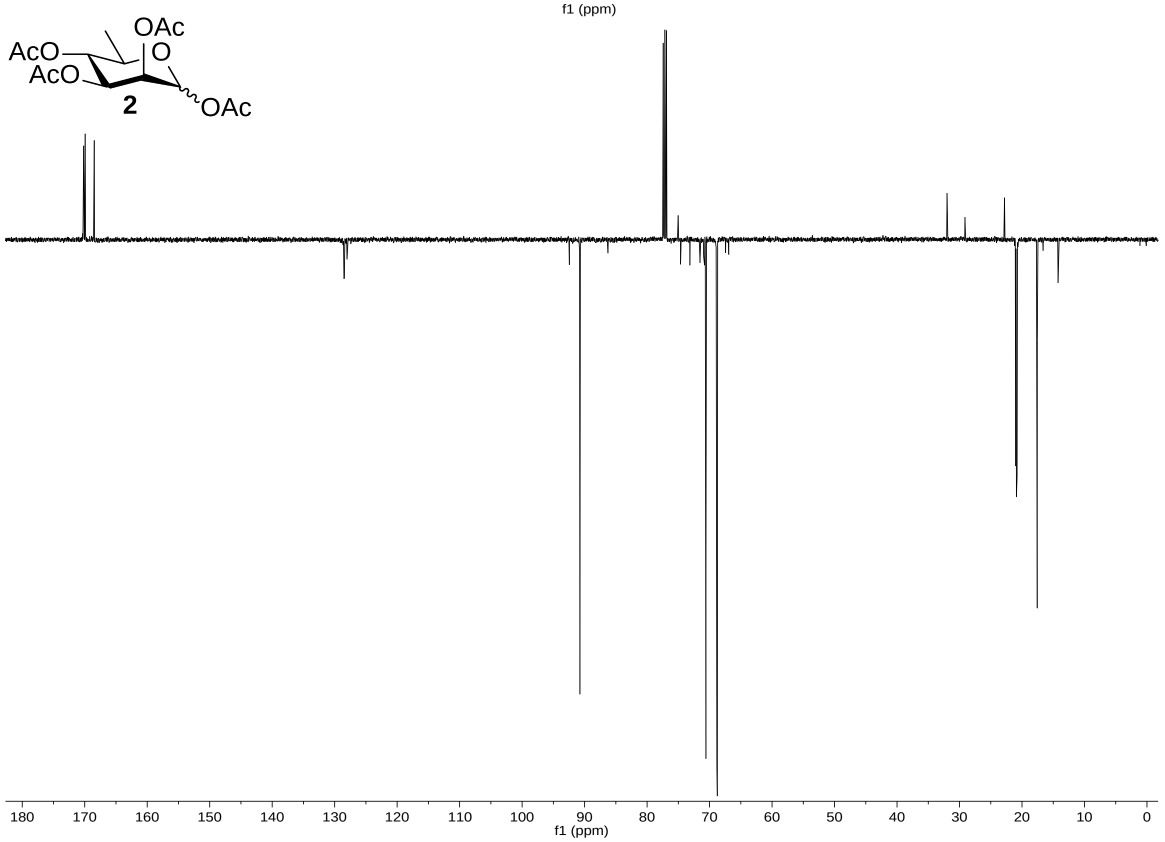
AcO
AcO
OAc
O
2
OAc
180
170
160
150
140
130
120
110
100
90
80
70
60
50
40
30
20
10
0
f1 (ppm)

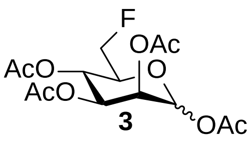
F
OAc
AcO
AcO
O
3
OAc

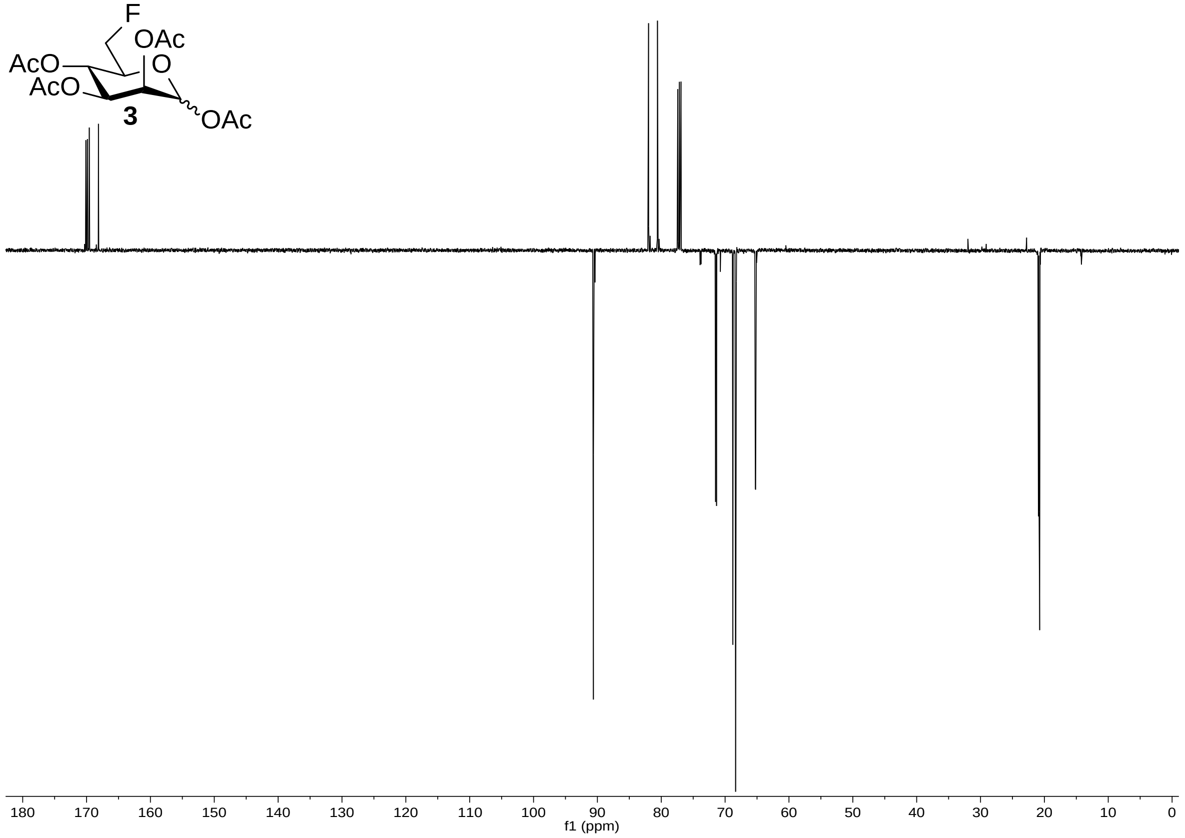
F
OAc
AcO
AcO
O
3
OAc
180
170
160
150
140
130
120
110
100
90
80
70
60
50
40
30
20
10
0
f1 (ppm)

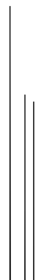

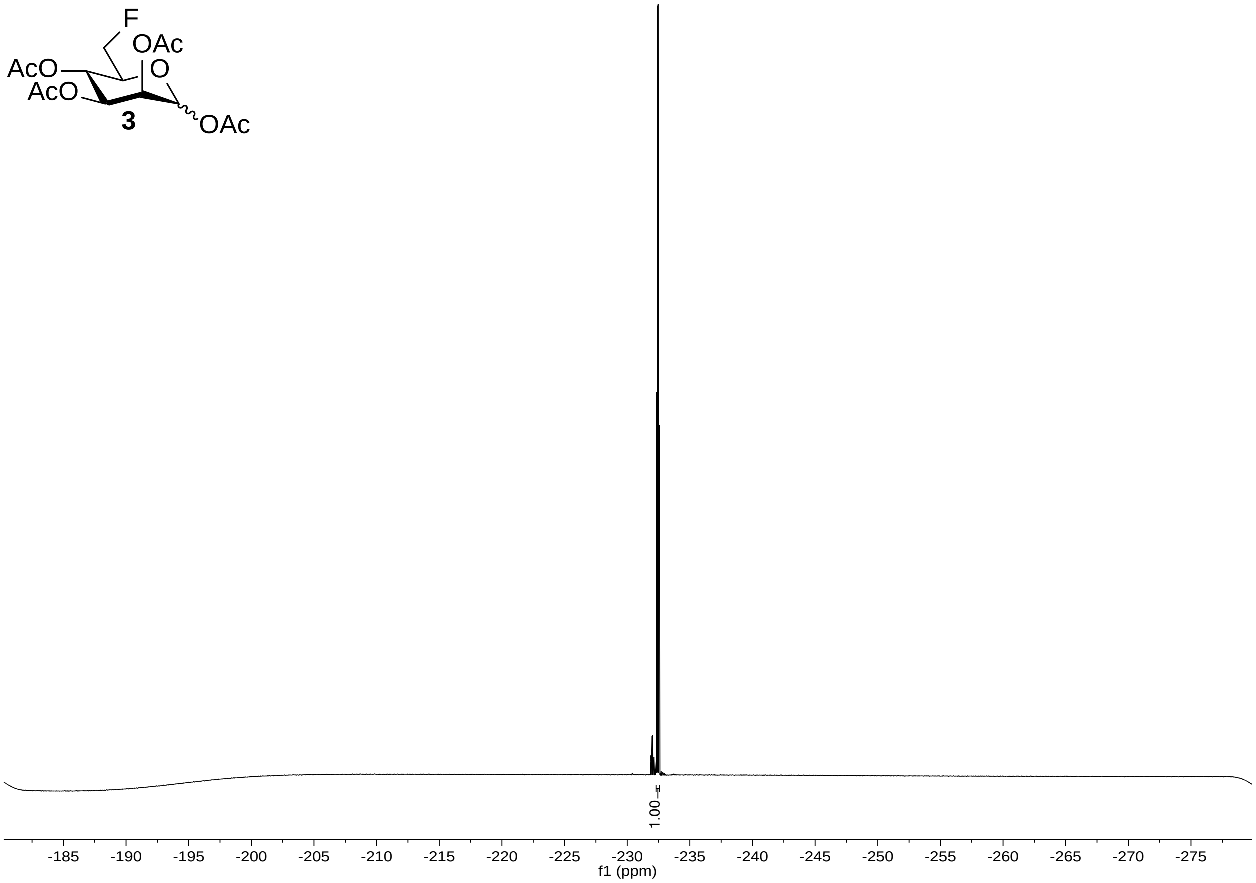
F
OAc
O
AcO
AcO
3
OAc
1.00
-185
-190
-195
-200
-205
-210
-215
-220
-225
-230
-235
-240
-245
-250
-255
-260
-265
-270
-275
f1 (ppm)

[EMPTY]

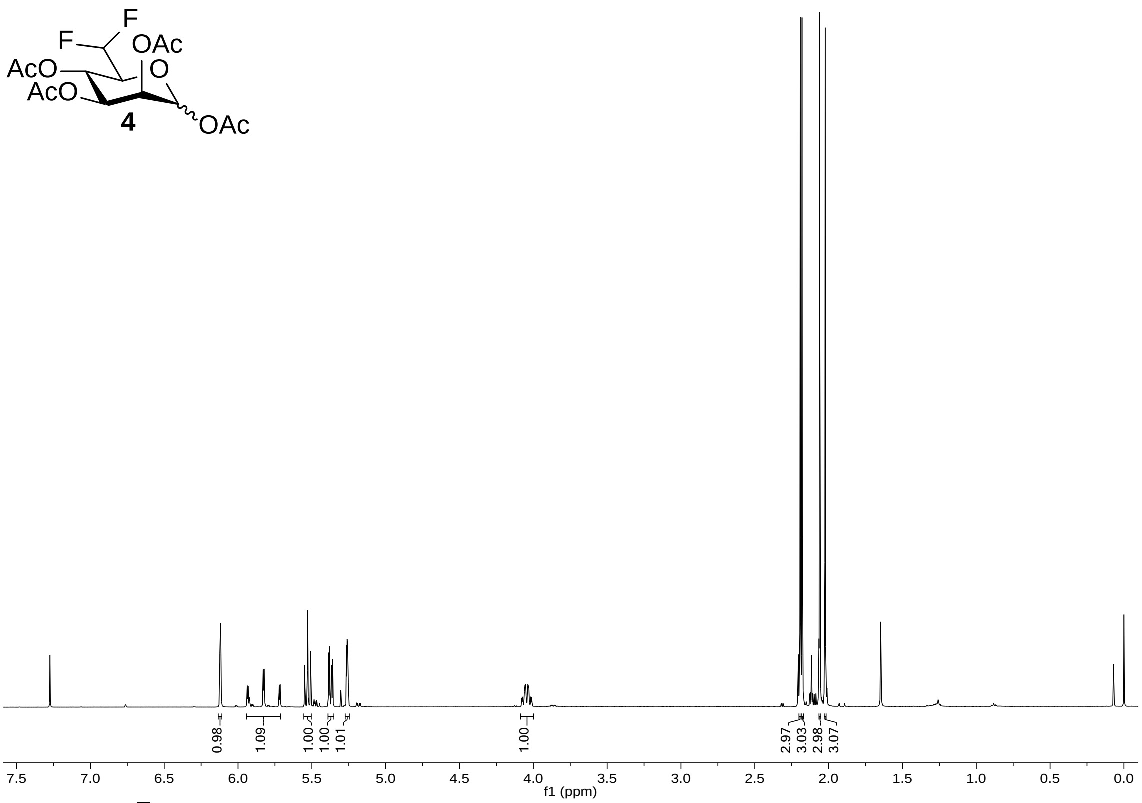
F
F
OAc
AcO
O
AcO
4
OAc
0.98
1.09
1.00
1.00
1.01
1.00
2.97
3.03
2.98
3.07
7.5 7.0 6.5 6.0 5.5 5.0 4.5 4.0 3.5 3.0 2.5 2.0 1.5 1.0 0.5 0.0
f1 (ppm)

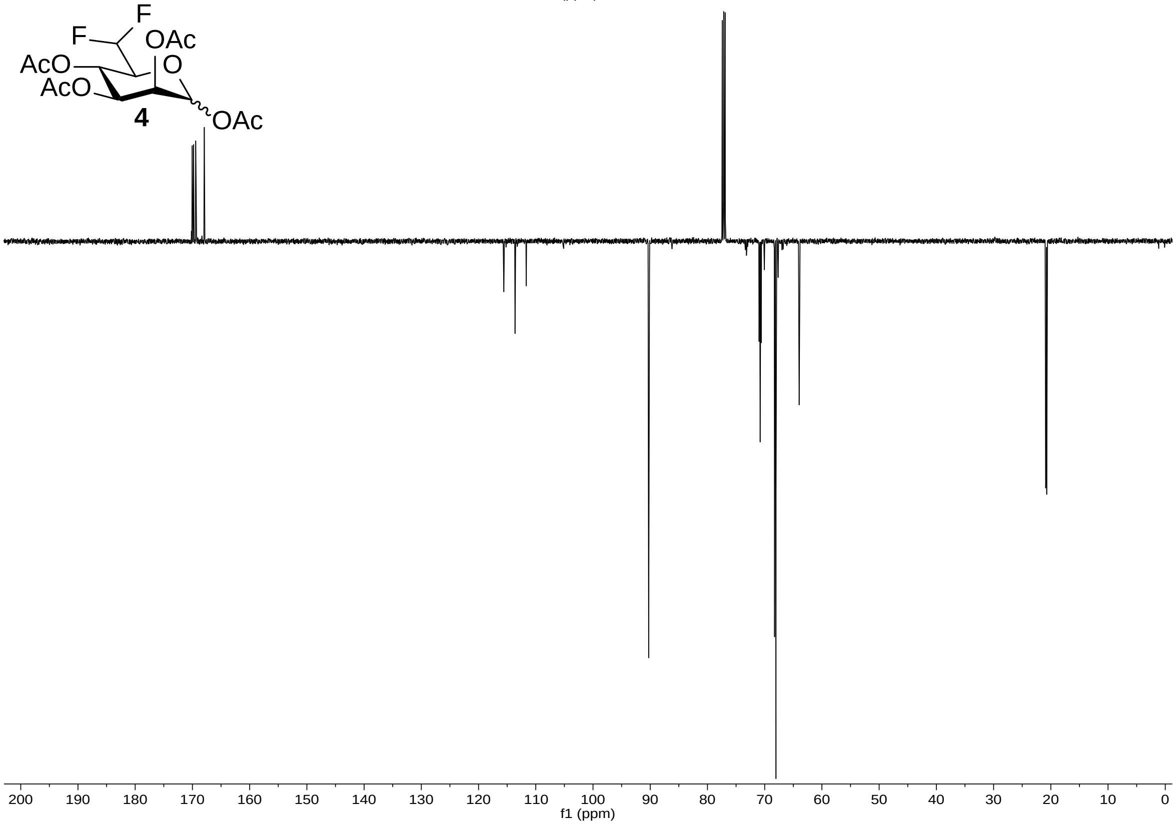
F
F
OAc
AcO
O
AcO
4
OAc
200 190 180 170 160 150 140 130 120 110 100 90 80 70 60 50 40 30 20 10 0
f1 (ppm)

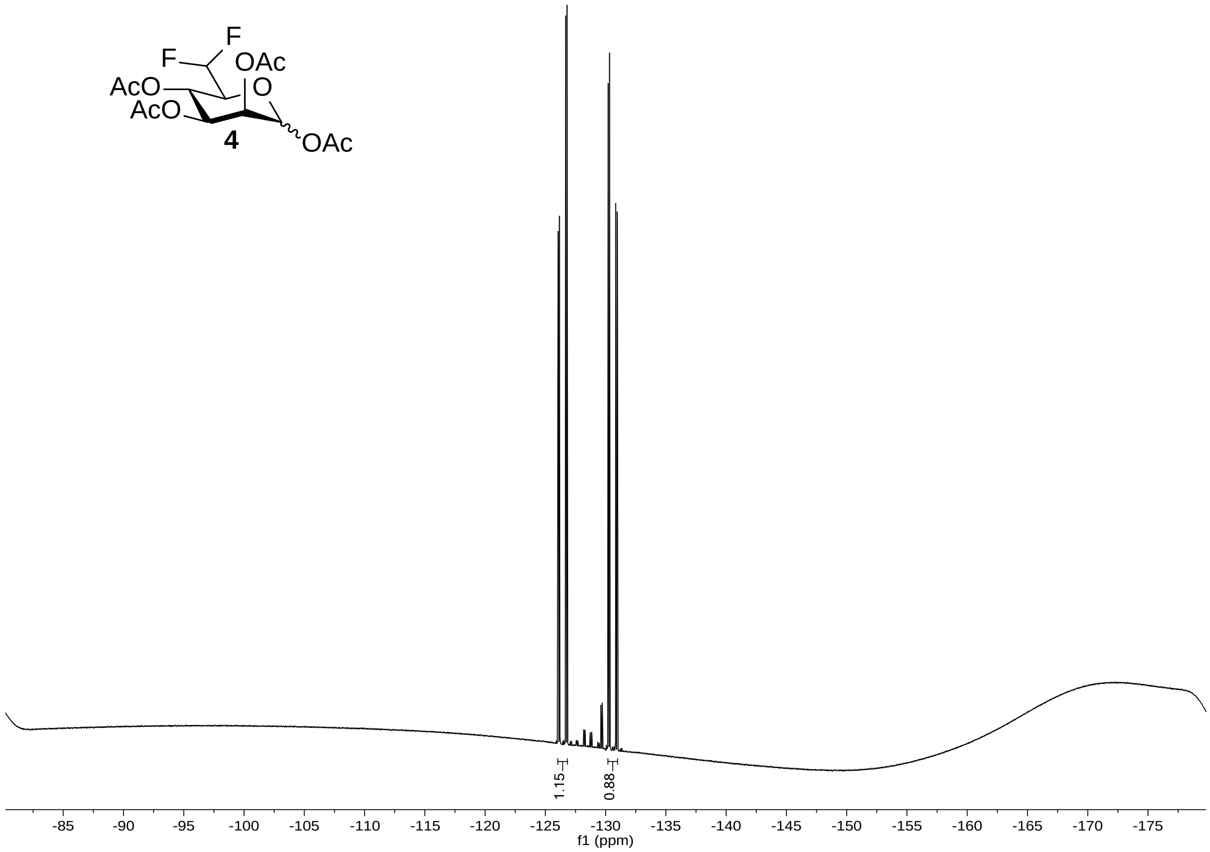
F
F
OAc
AcO
AcO
O
OAc
1.15
0.88
-85
-90
-95
-100
-105
-110
-115
-120
-125
-130
-135
-140
-145
-150
-155
-160
-165
-170
-175
f1 (ppm)

4

[EMPTY]

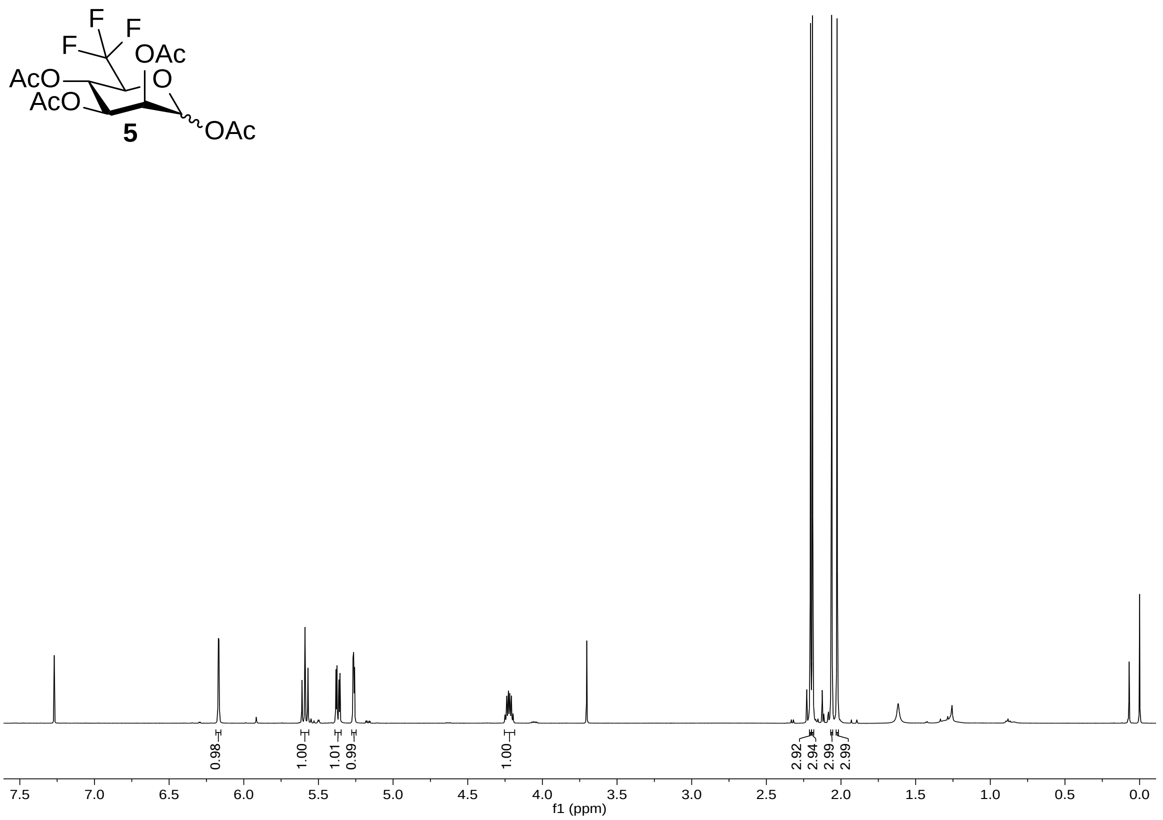
F
F
F
OAc
AcO
AcO
O
OAc
5
0.98
1.00
1.01
0.99
1.00
2.92
2.94
2.99
2.99
7.5 7.0 6.5 6.0 5.5 5.0 4.5 4.0 3.5 3.0 2.5 2.0 1.5 1.0 0.5 0.0
f1 (ppm)

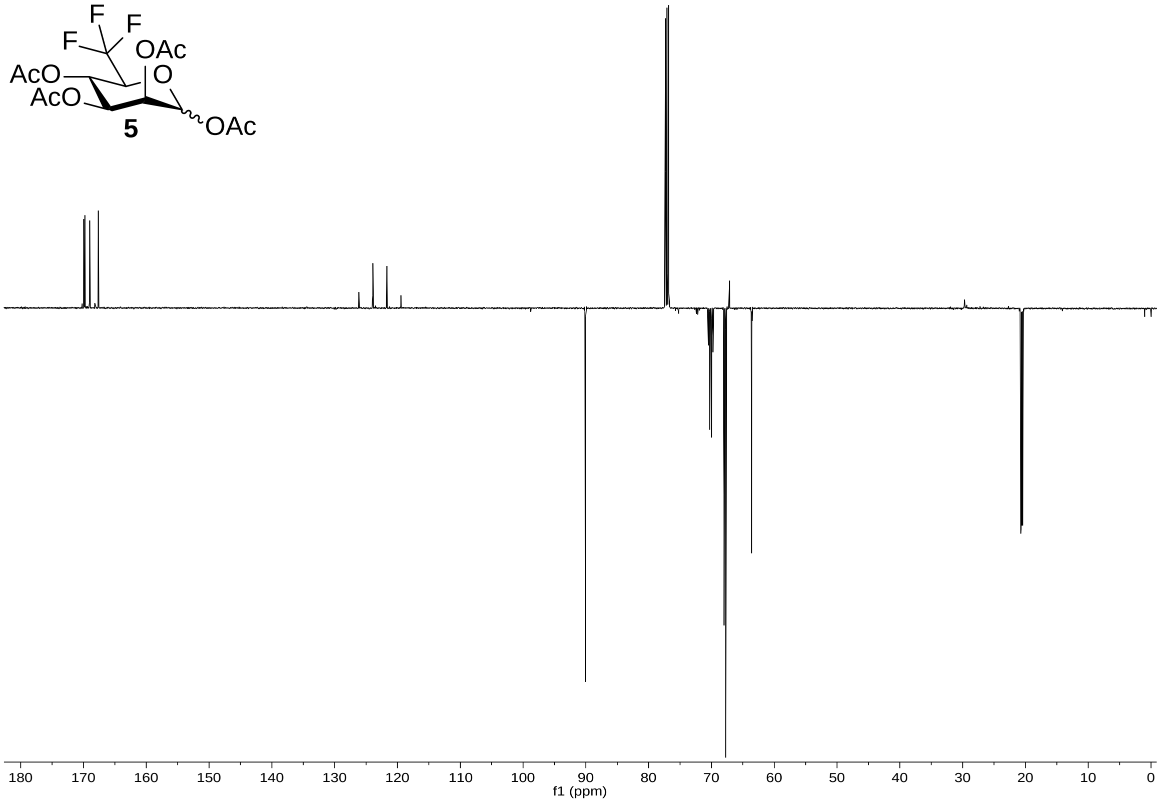
F
F
F
OAc
AcO
AcO
O
OAc
5
180 170 160 150 140 130 120 110 100 90 80 70 60 50 40 30 20 10 0
f1 (ppm)

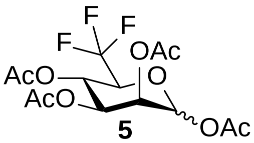

5

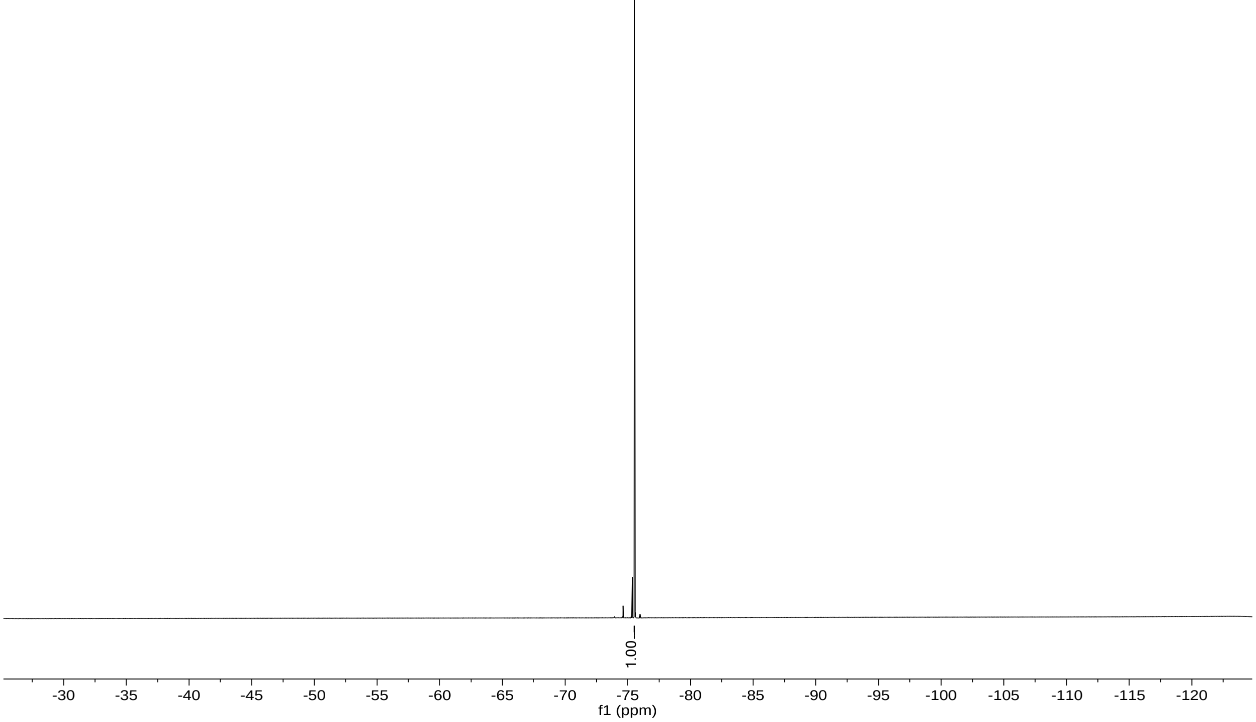
1.00
-30
-35
-40
-45
-50
-55
-60
-65
-70
-75
-80
-85
-90
-95
-100
-105
-110
-115
-120
f1 (ppm)

[EMPTY]

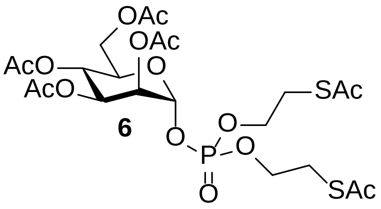
OAc
OAc
AcO
AcO
O
SAc
6
O
O
P
O
O
SAc

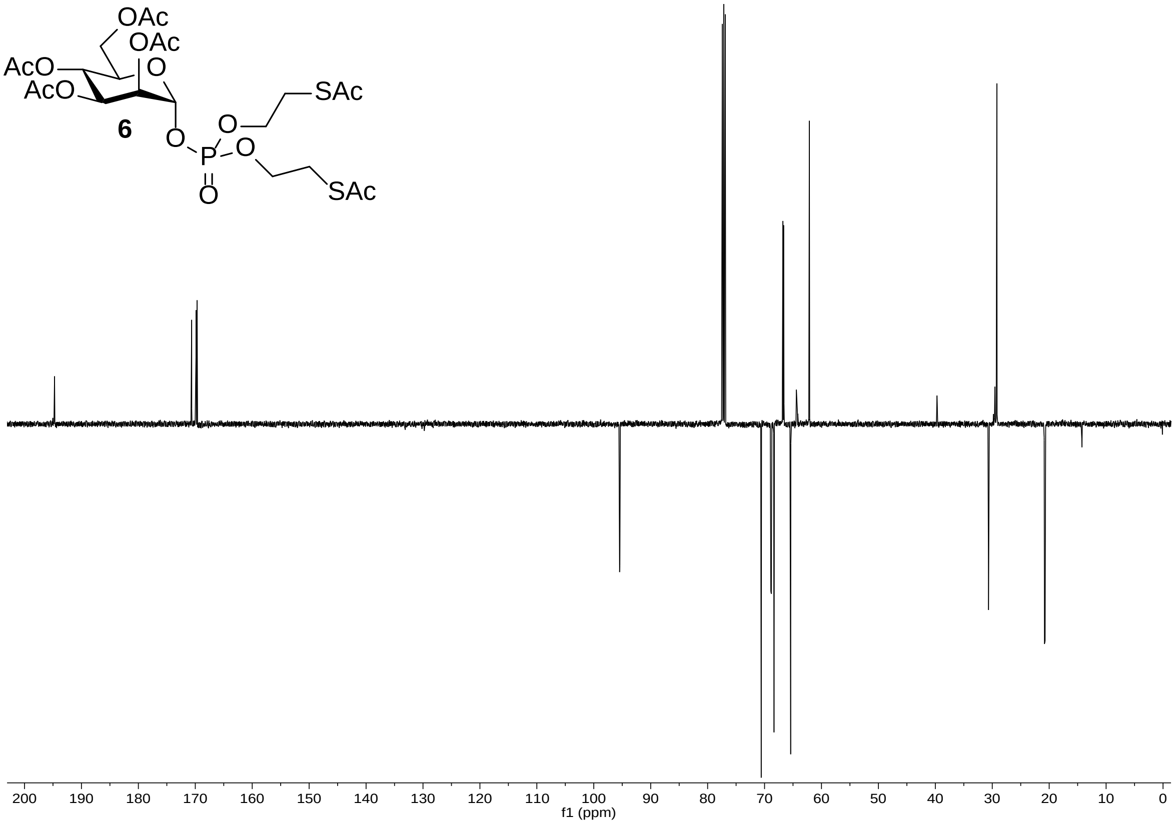
OAc
OAc
AcO
AcO
O
SAc
6
O
O
P
O
O
SAc
200 190 180 170 160 150 140 130 120 110 100 90 80 70 60 50 40 30 20 10 0
f1 (ppm)

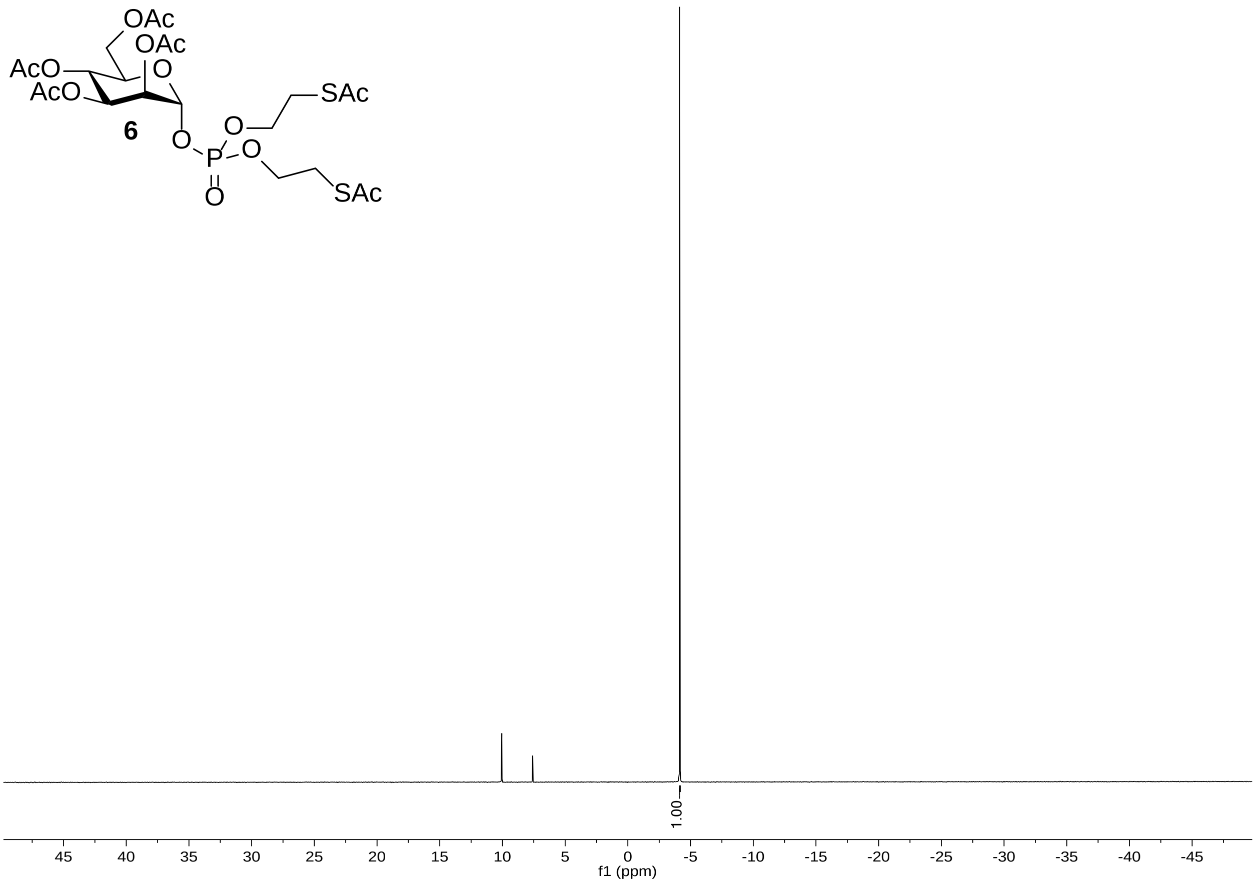
OAc
OAc
AcO
AcO
O
6
O
O
P
O
O
SAc
SAc
1.00
45
40
35
30
25
20
15
10
5
0
-5
-10
-15
-20
-25
-30
-35
-40
-45
f1 (ppm)

[EMPTY]

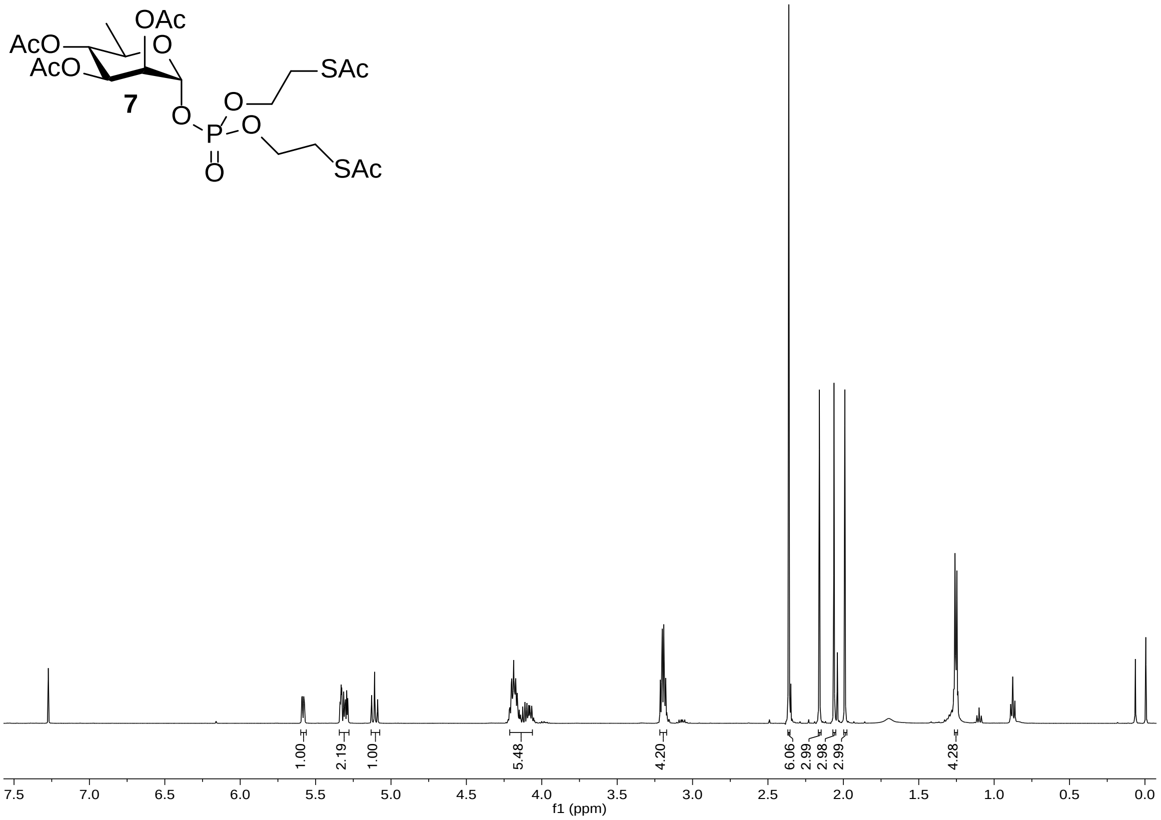
OAc
AcO
AcO
O
7
O
P
O
O
O
SAc
SAc
1.00
2.19
1.00
5.48
4.20
6.06
2.99
2.98
2.99
4.28
7.5 7.0 6.5 6.0 5.5 5.0 4.5 4.0 3.5 3.0 2.5 2.0 1.5 1.0 0.5 0.0
f1 (ppm)

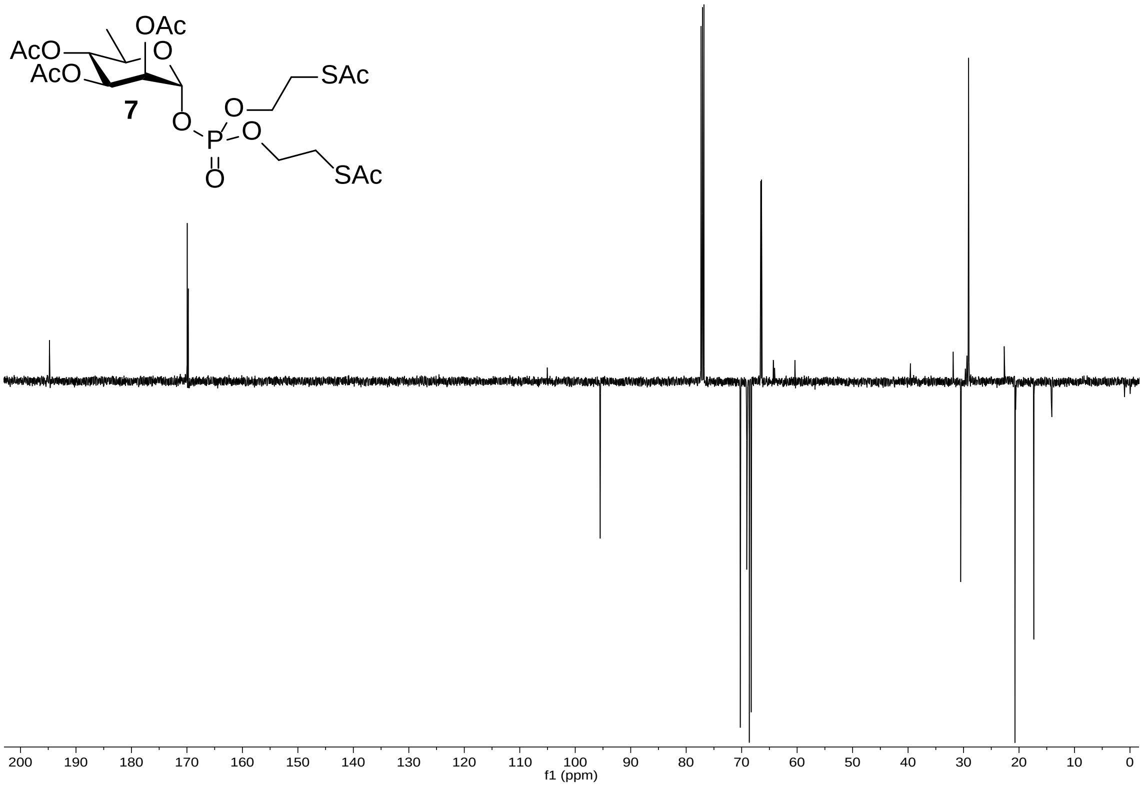
OAc
AcO
AcO
O
7
O
P
O
O
O
SAc
SAc
200 190 180 170 160 150 140 130 120 110 100 90 80 70 60 50 40 30 20 10 0
f1 (ppm)

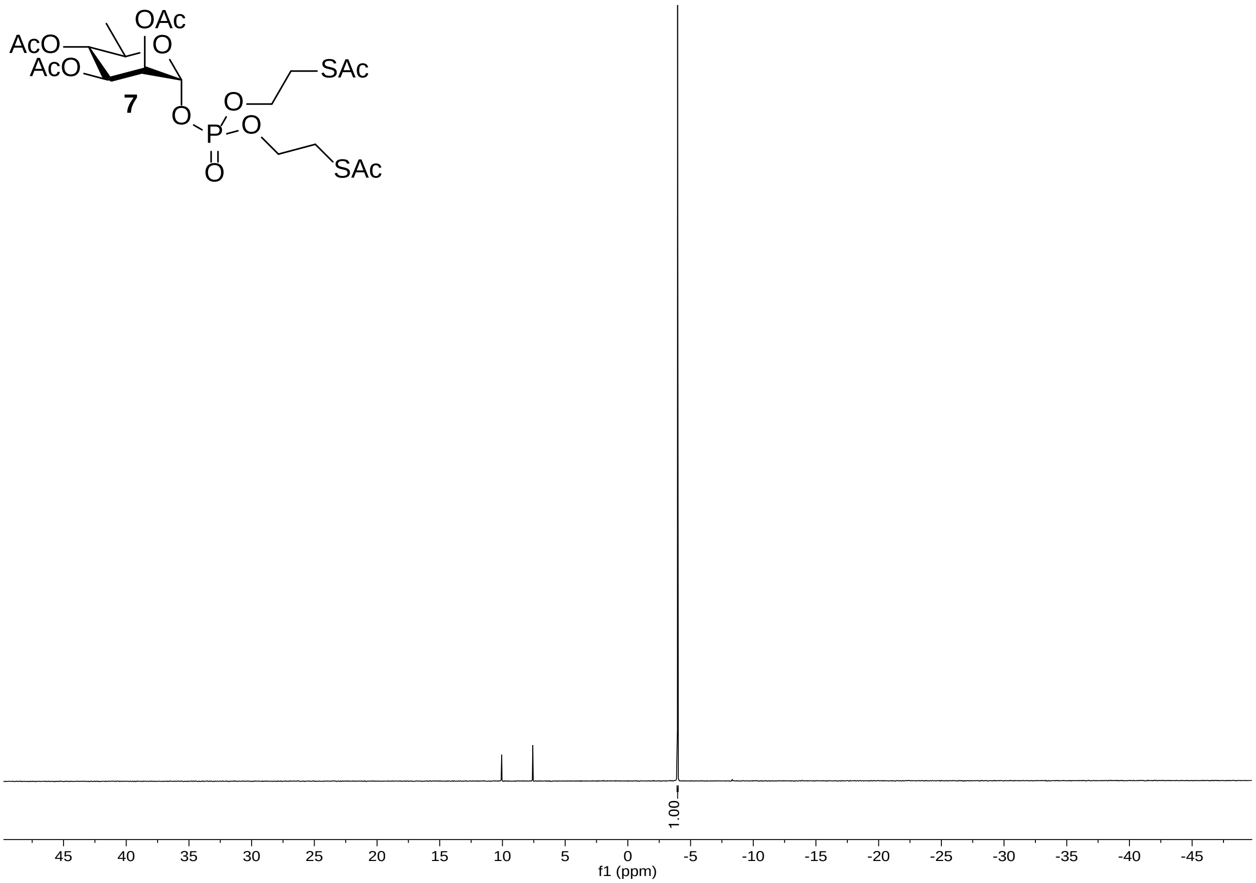
OAc
AcO
AcO
O
7
O
P
O
O
O
SAc
SAc
1.00
45 40 35 30 25 20 15 10 5 0 -5 -10 -15 -20 -25 -30 -35 -40 -45
f1 (ppm)

[EMPTY]

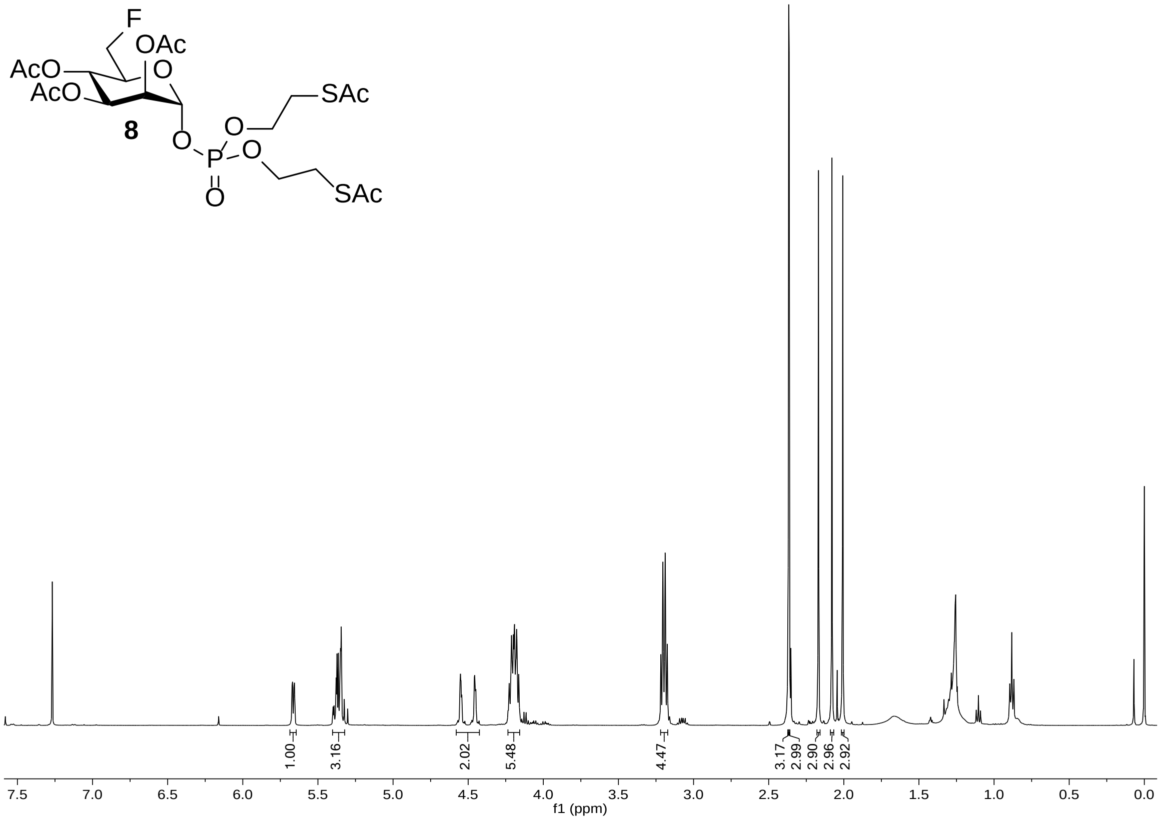
F
OAc
AcO
AcO
O
8
SAc
O
O
P
O
O
SAc
1.00
3.16
2.02
5.48
4.47
3.17
2.99
2.90
2.96
2.92
7.5
7.0
6.5
6.0
5.5
5.0
4.5
4.0
3.5
3.0
2.5
2.0
1.5
1.0
0.5
0.0
f1 (ppm)

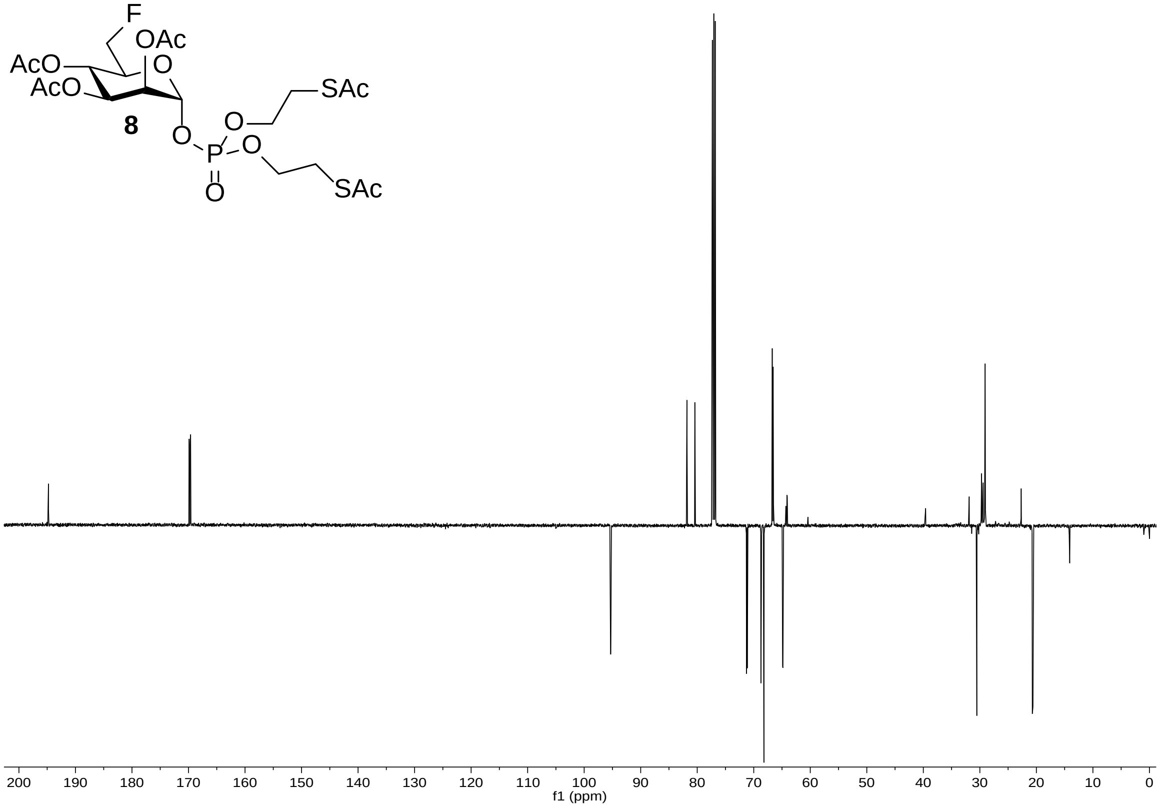
F
OAc
AcO
AcO
O
8
SAc
O
O
P
O
O
SAc
200
190
180
170
160
150
140
130
120
110
100
90
80
70
60
50
40
30
20
10
0
f1 (ppm)

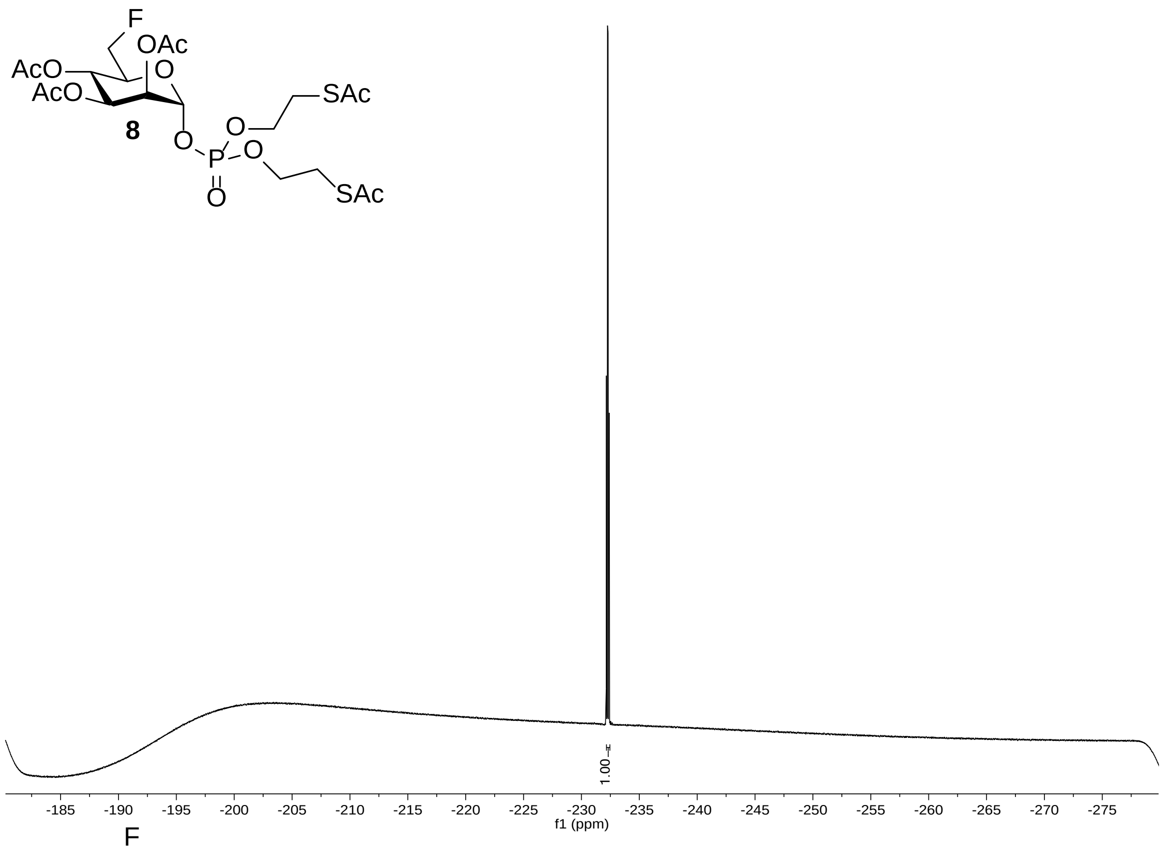
F
OAc
AcO
AcO
O
8
O
P
O
O
O
SAc
SAc
1.00
-185
-190
-195
-200
-205
-210
-215
-220
-225
-230
-235
-240
-245
-250
-255
-260
-265
-270
-275
f1 (ppm)

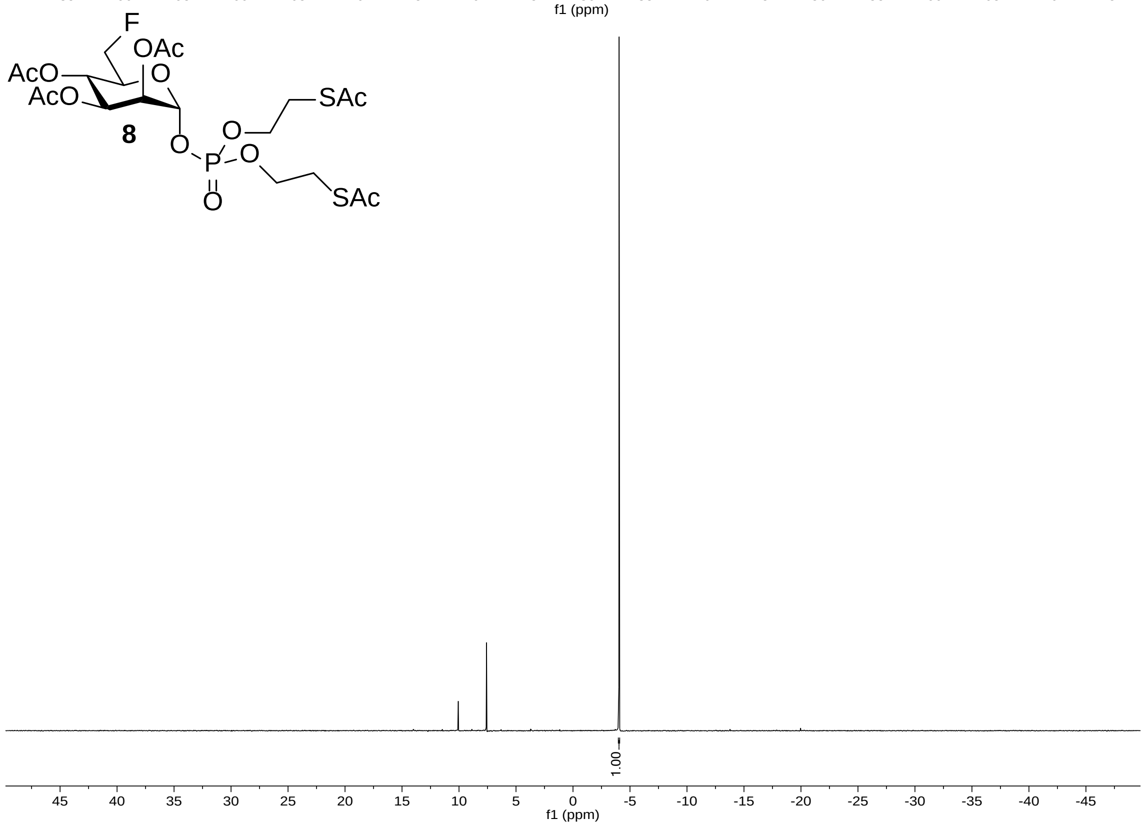
F
OAc
AcO
AcO
O
8
O
P
O
O
O
SAc
SAc
1.00
45
40
35
30
25
20
15
10
5
0
-5
-10
-15
-20
-25
-30
-35
-40
-45
f1 (ppm)

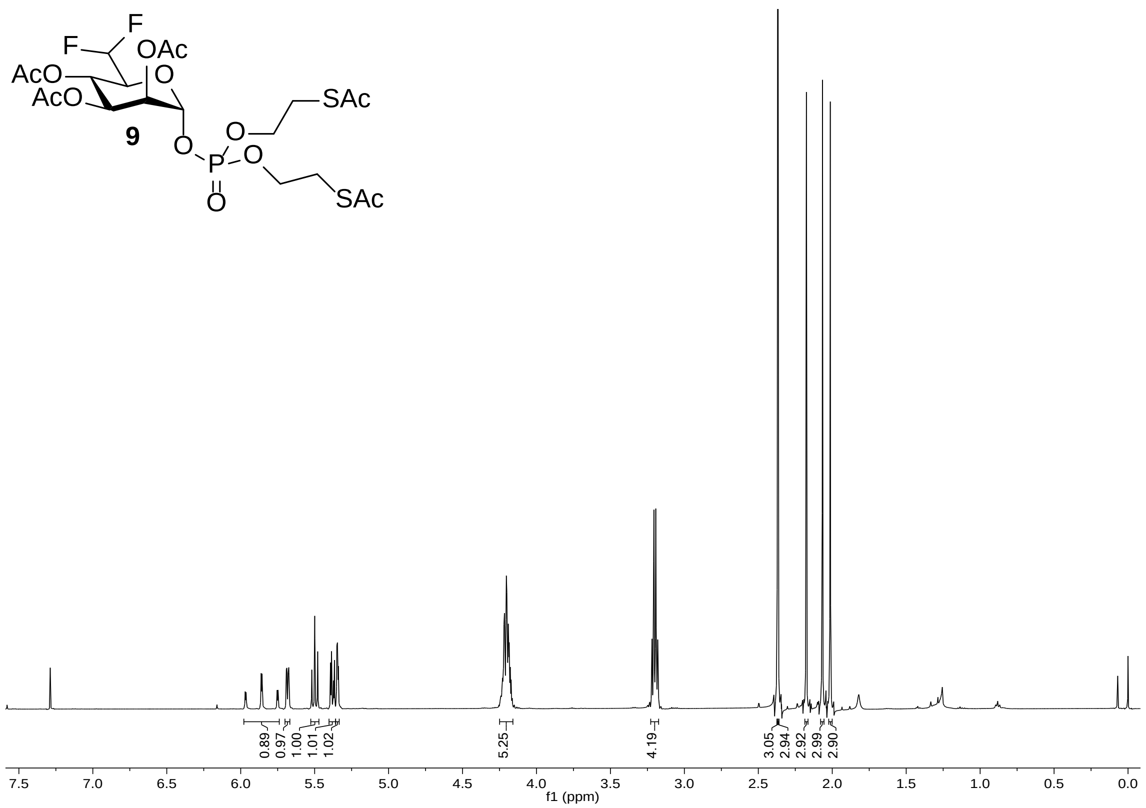
F
F
OAc
AcO
AcO
O
9
O
P
O
O
SAc
SAc
0.89
0.97
1.00
1.01
1.02
5.25
4.19
3.05
2.94
2.92
2.99
2.90
7.5 7.0 6.5 6.0 5.5 5.0 4.5 4.0 3.5 3.0 2.5 2.0 1.5 1.0 0.5 0.0
f1 (ppm)

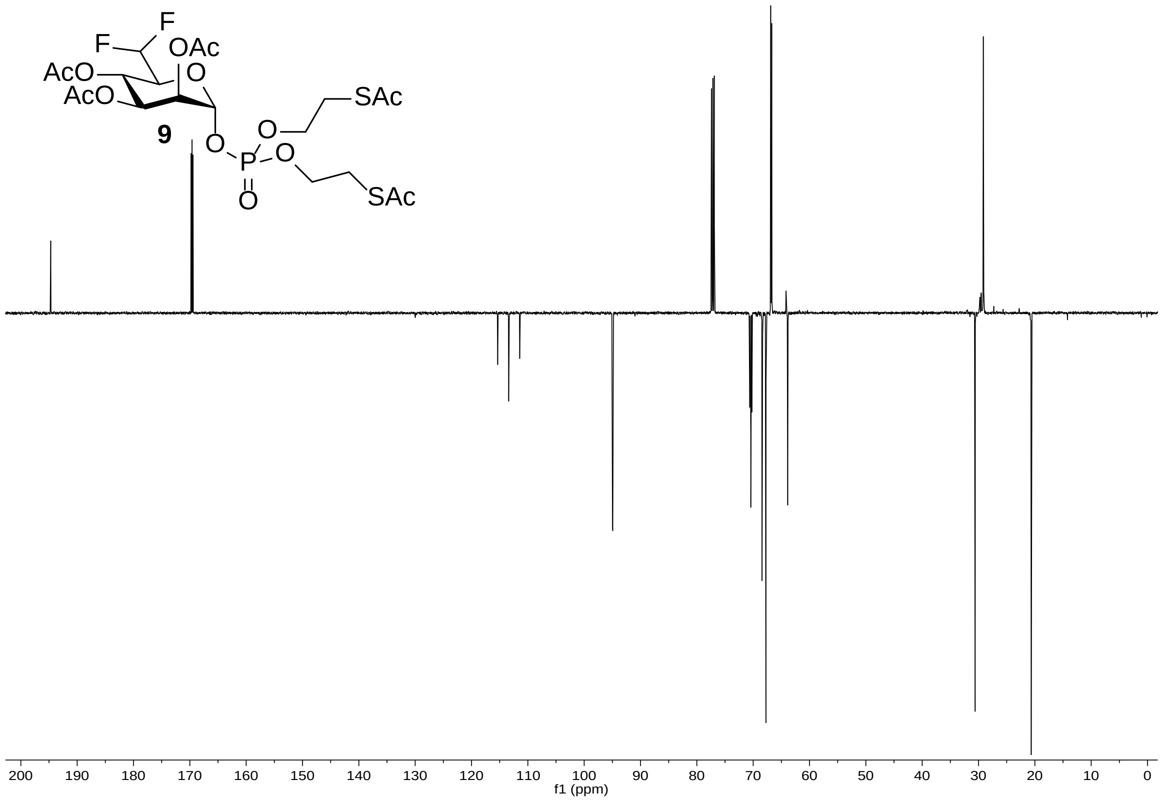
F
F
OAc
AcO
AcO
O
9
O
P
O
O
SAc
SAc
200 190 180 170 160 150 140 130 120 110 100 90 80 70 60 50 40 30 20 10 0
f1 (ppm)

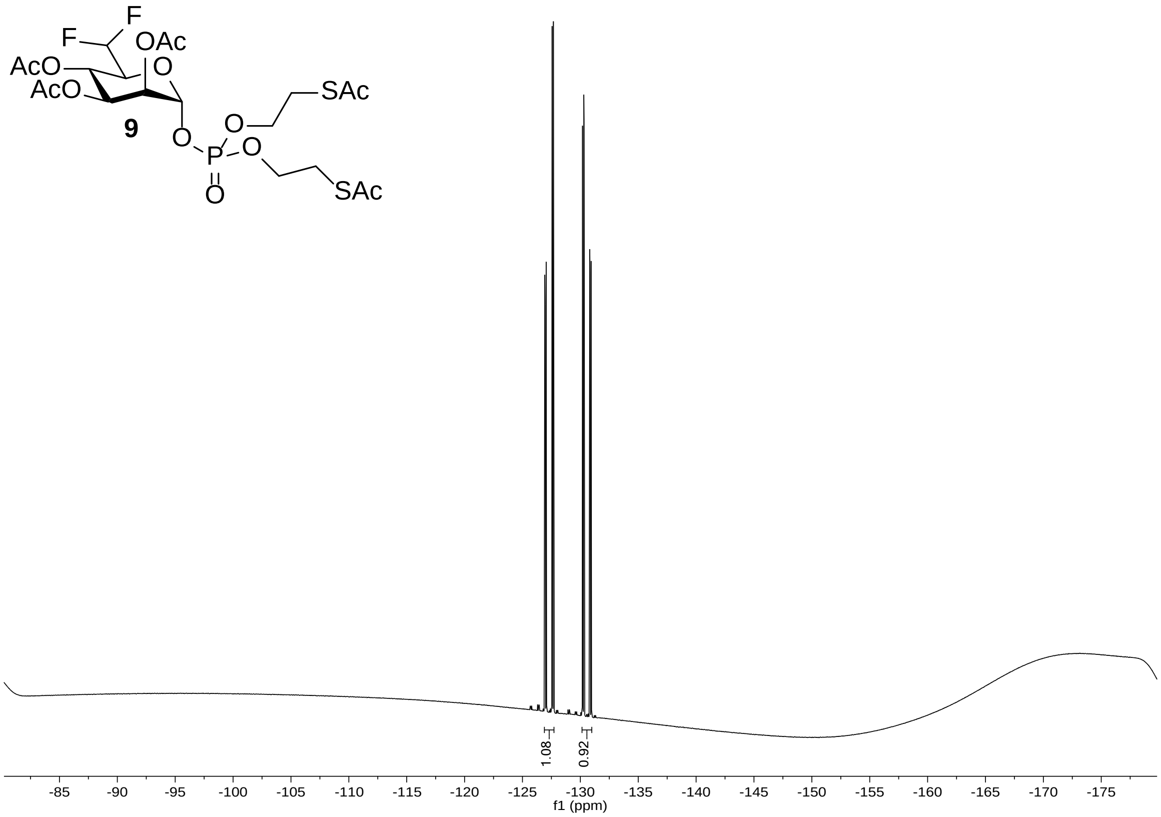
F
F
OAc
AcO
O
AcO
9
O
O
P
O
O
SAc
SAc
1.08
0.92
-85
-90
-95
-100
-105
-110
-115
-120
-125
-130
-135
-140
-145
-150
-155
-160
-165
-170
-175
f1 (ppm)

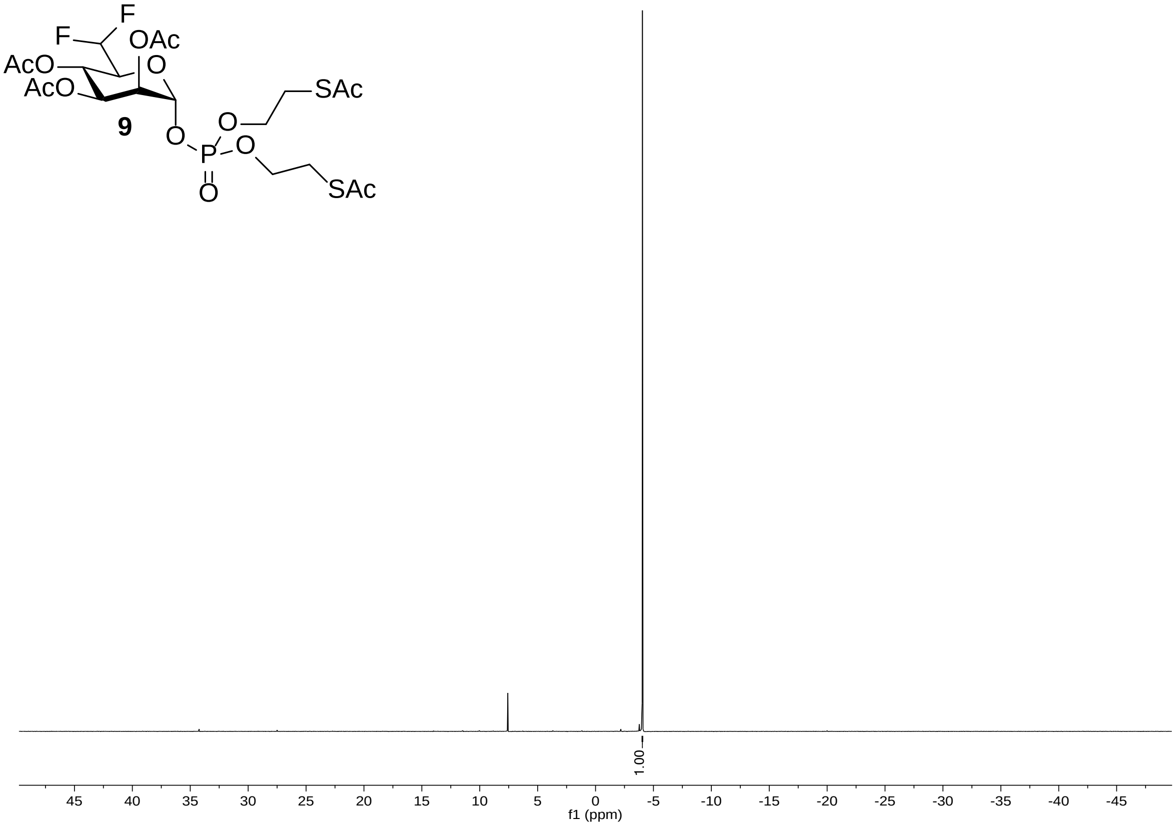
F
F
OAc
AcO
O
AcO
9
O
O
P
O
O
SAc
SAc
1.00
45
40
35
30
25
20
15
10
5
0
-5
-10
-15
-20
-25
-30
-35
-40
-45
f1 (ppm)

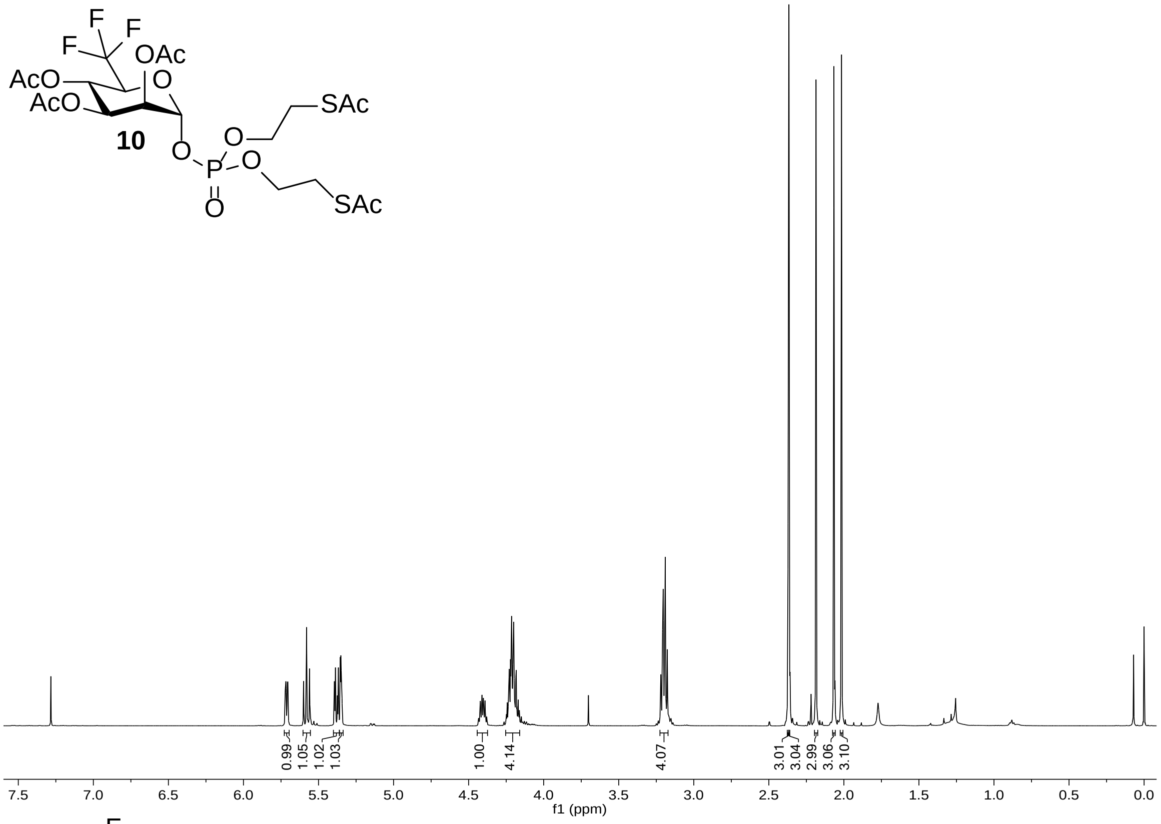
F
F
F
OAc
AcO
AcO
O
10
O
O
P
O
O
SAc
SAc
0.99
1.05
1.02
1.03
1.00
4.14
4.07
3.01
3.04
2.99
3.06
3.10
7.5
7.0
6.5
6.0
5.5
5.0
4.5
4.0
3.5
3.0
2.5
2.0
1.5
1.0
0.5
0.0
f1 (ppm)

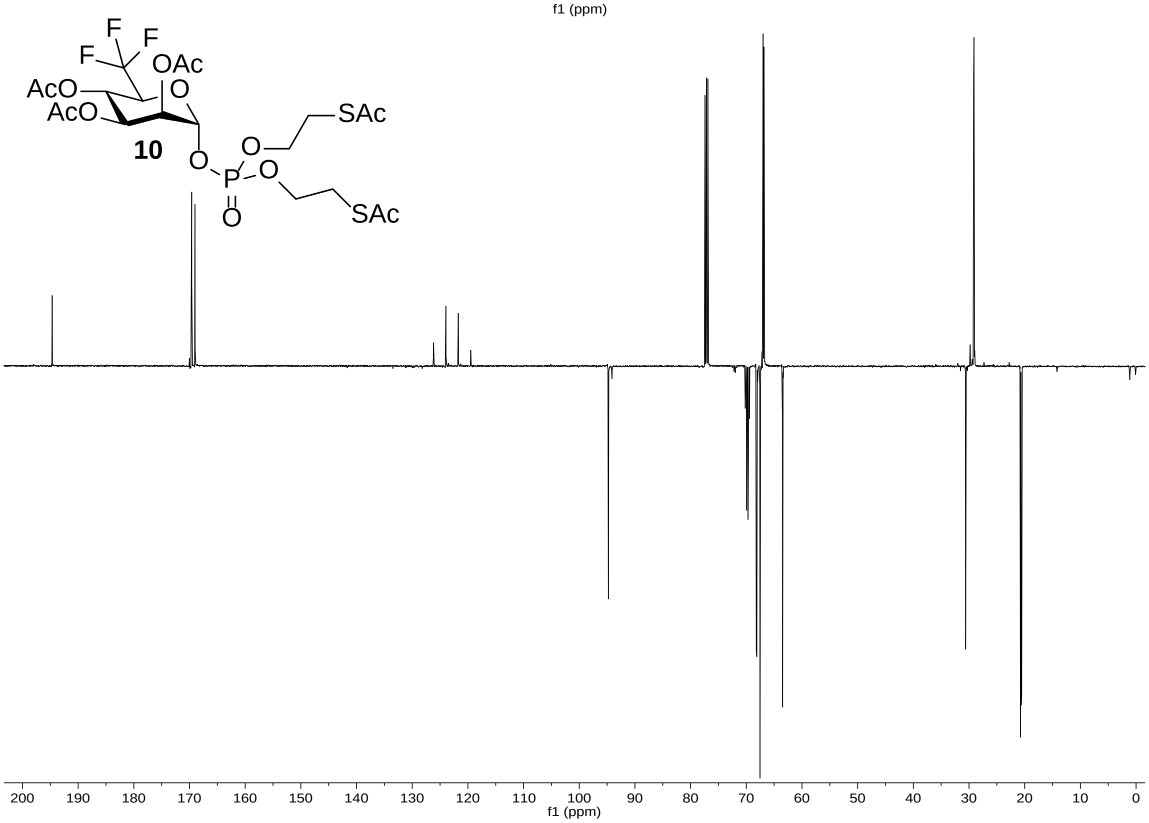
F
F
F
OAc
AcO
AcO
O
10
O
O
P
O
O
SAc
SAc
200
190
180
170
160
150
140
130
120
110
100
90
80
70
60
50
40
30
20
10
0
f1 (ppm)

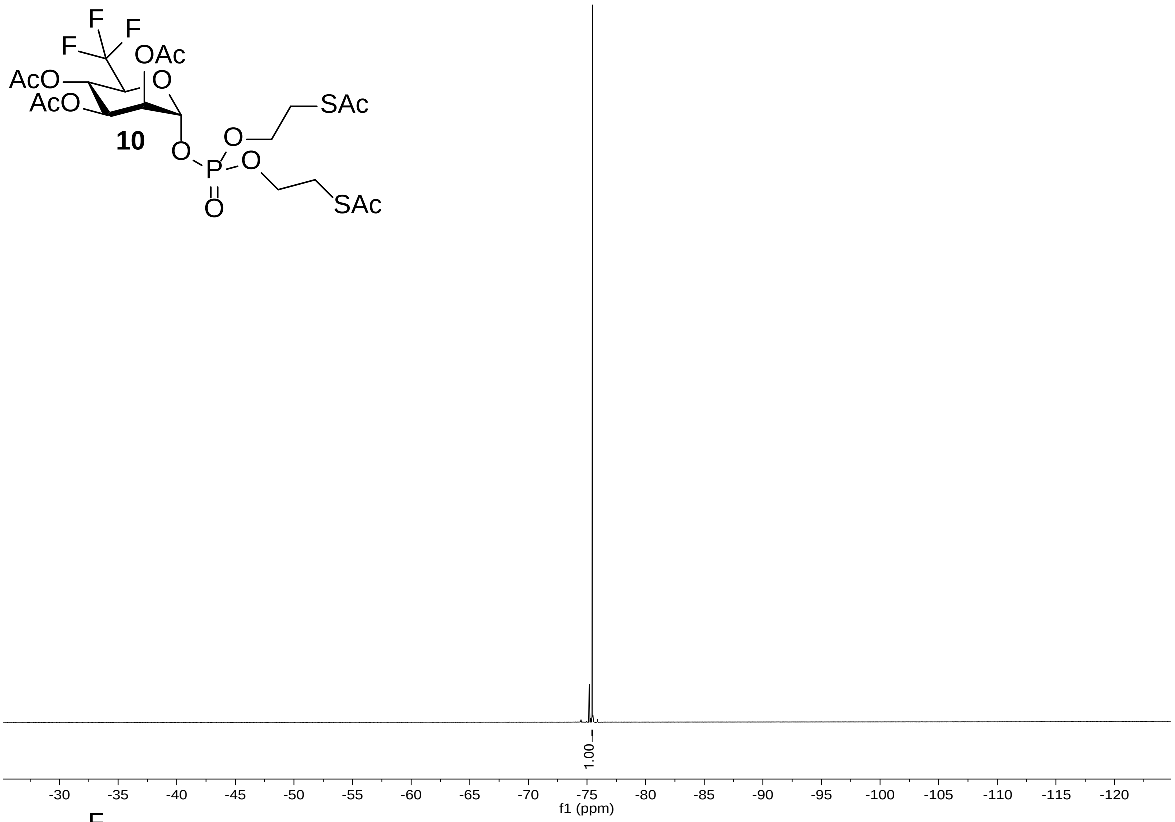
F
F
F
OAc
AcO
AcO
O
SAc
10
O
O
P
O
O
SAc
1.00
-30 -35 -40 -45 -50 -55 -60 -65 -70 -75 -80 -85 -90 -95 -100 -105 -110 -115 -120
f1 (ppm)

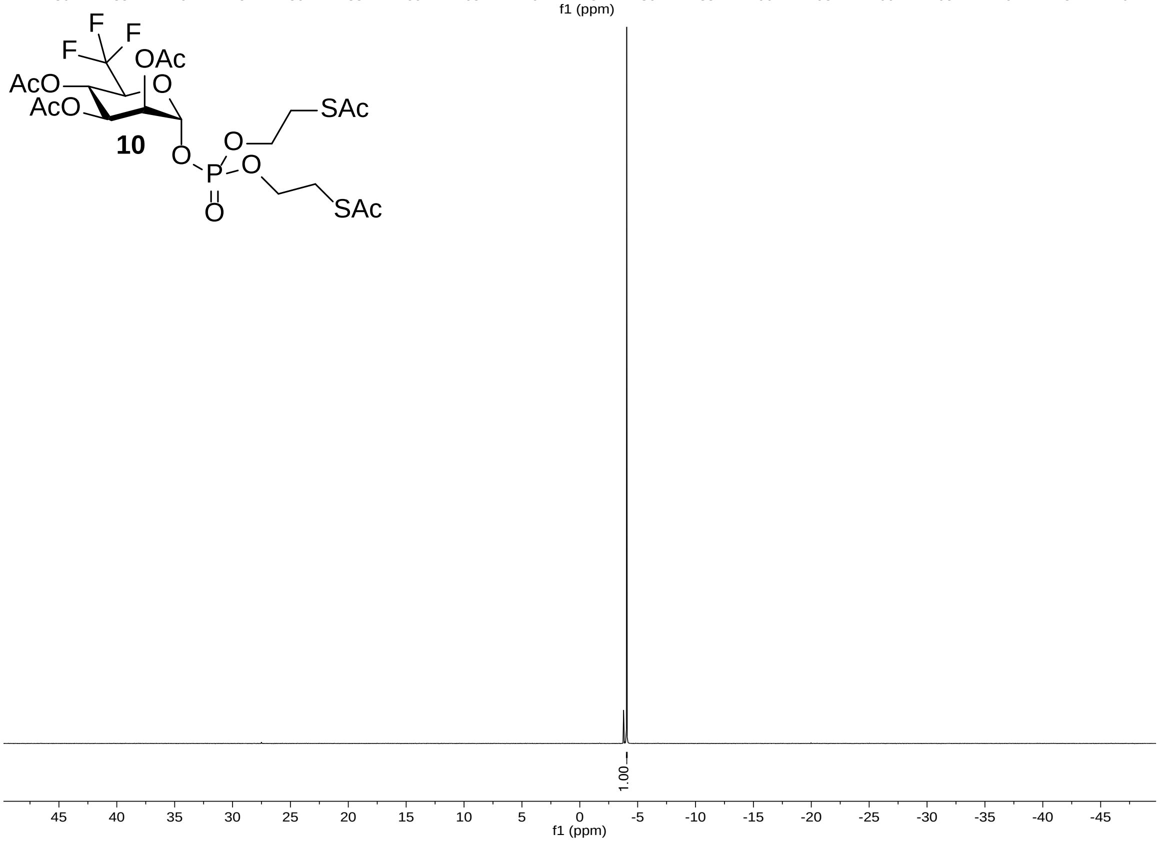
F
F
F
OAc
AcO
AcO
O
SAc
10
O
O
P
O
O
SAc
1.00
45 40 35 30 25 20 15 10 5 0 -5 -10 -15 -20 -25 -30 -35 -40 -45
f1 (ppm)

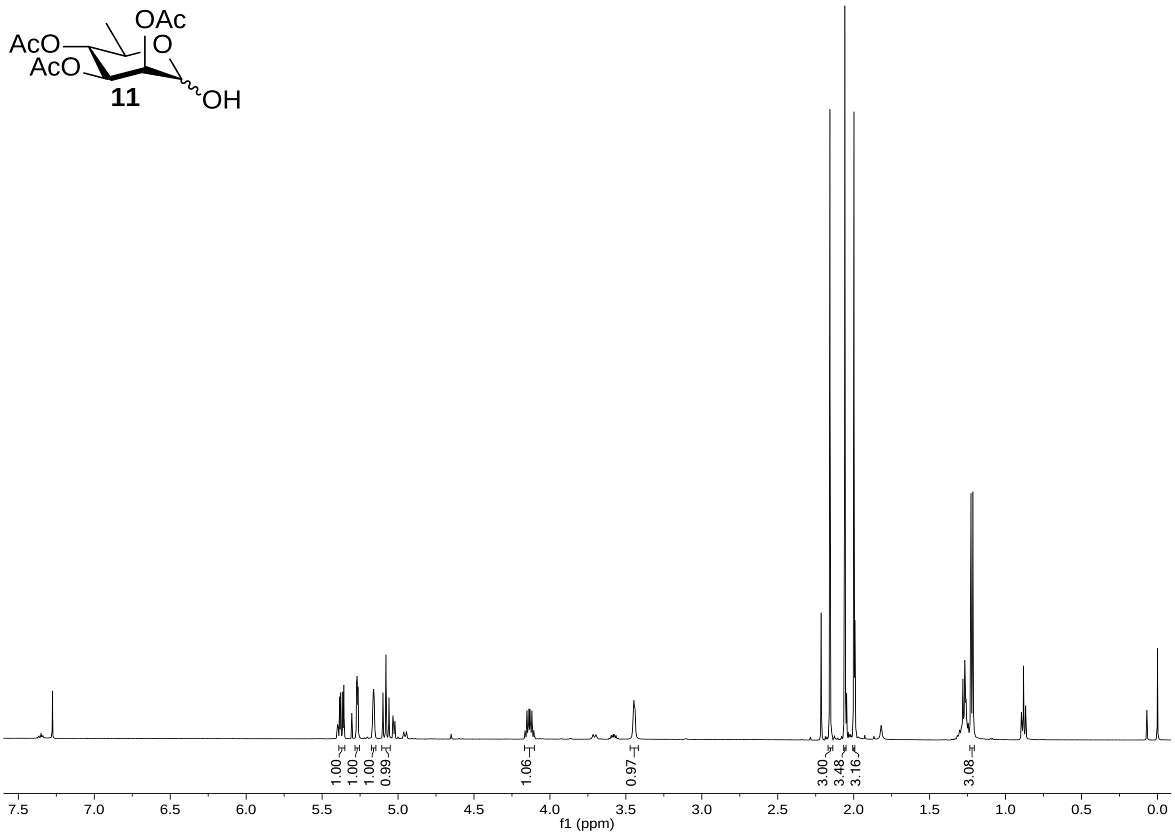
OAc
AcO
AcO
O
11
OH
1.00
1.00
1.00
0.99
1.06
0.97
3.00
3.48
3.16
3.08
7.5 7.0 6.5 6.0 5.5 5.0 4.5 4.0 3.5 3.0 2.5 2.0 1.5 1.0 0.5 0.0
f1 (ppm)

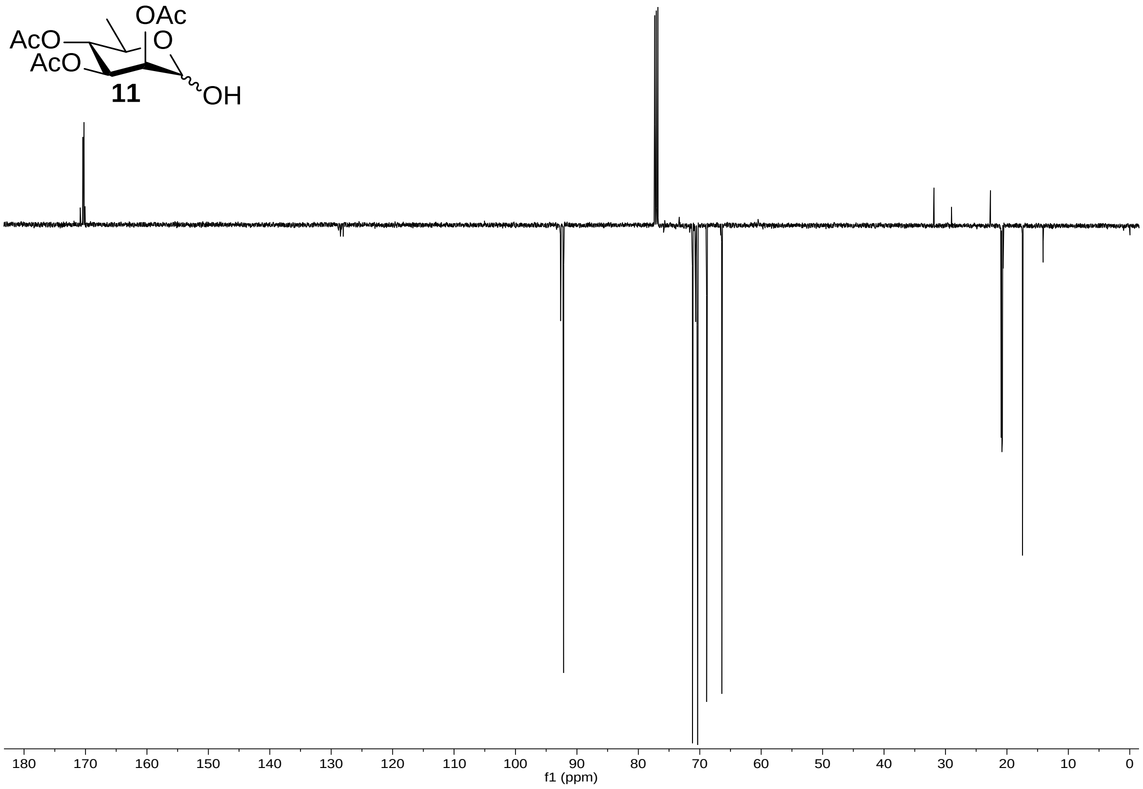
OAc
AcO
AcO
O
11
OH
180 170 160 150 140 130 120 110 100 90 80 70 60 50 40 30 20 10 0
f1 (ppm)

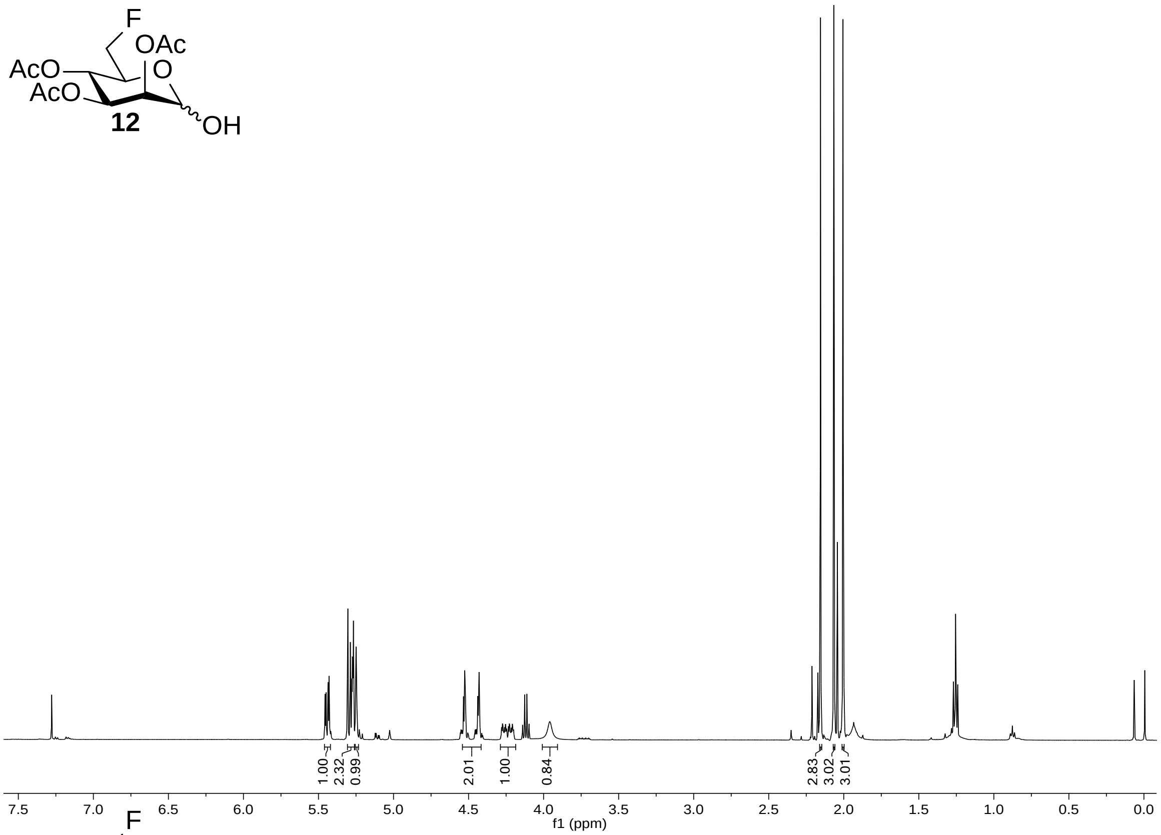
F
OAc
AcO
AcO
O
12
OH
1.00
2.32
0.99
2.01
1.00
0.84
2.83
3.02
3.01
7.5 7.0 6.5 6.0 5.5 5.0 4.5 4.0 3.5 3.0 2.5 2.0 1.5 1.0 0.5 0.0
f1 (ppm)

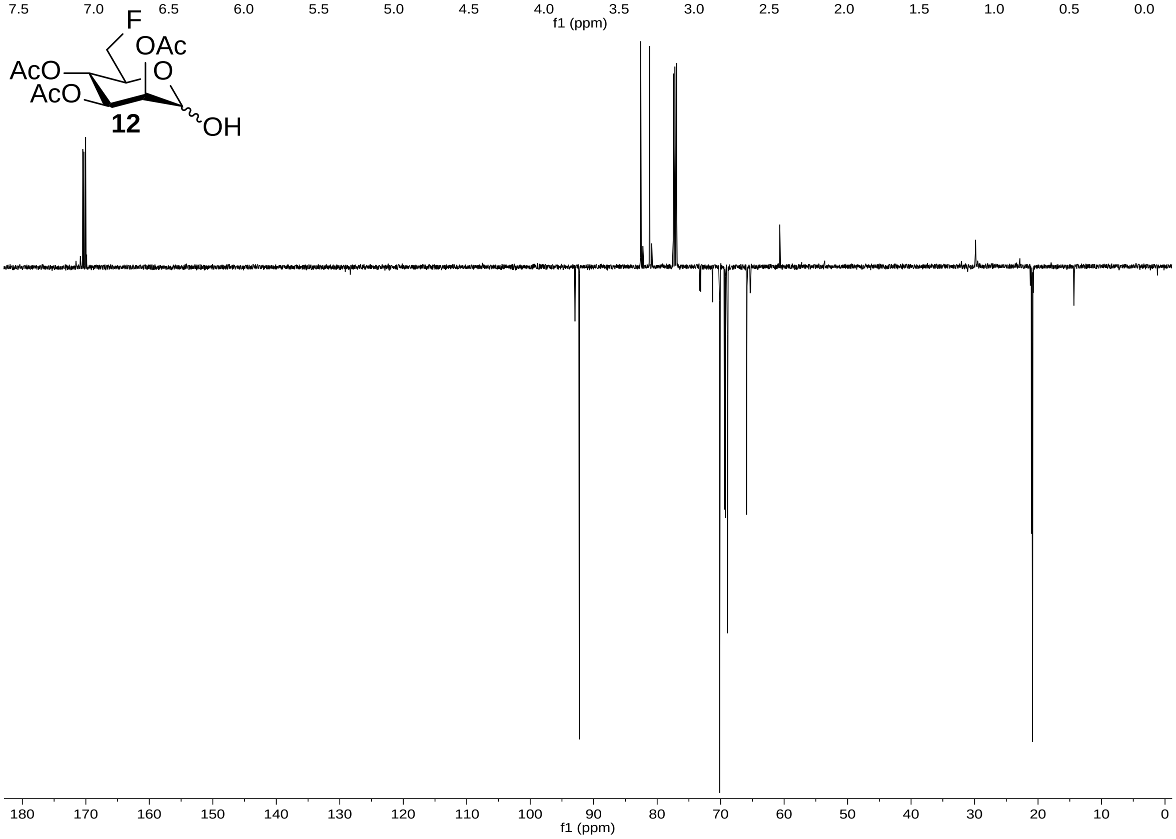
F
OAc
AcO
AcO
O
12
OH
180 170 160 150 140 130 120 110 100 90 80 70 60 50 40 30 20 10 0
f1 (ppm)

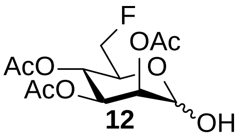
12

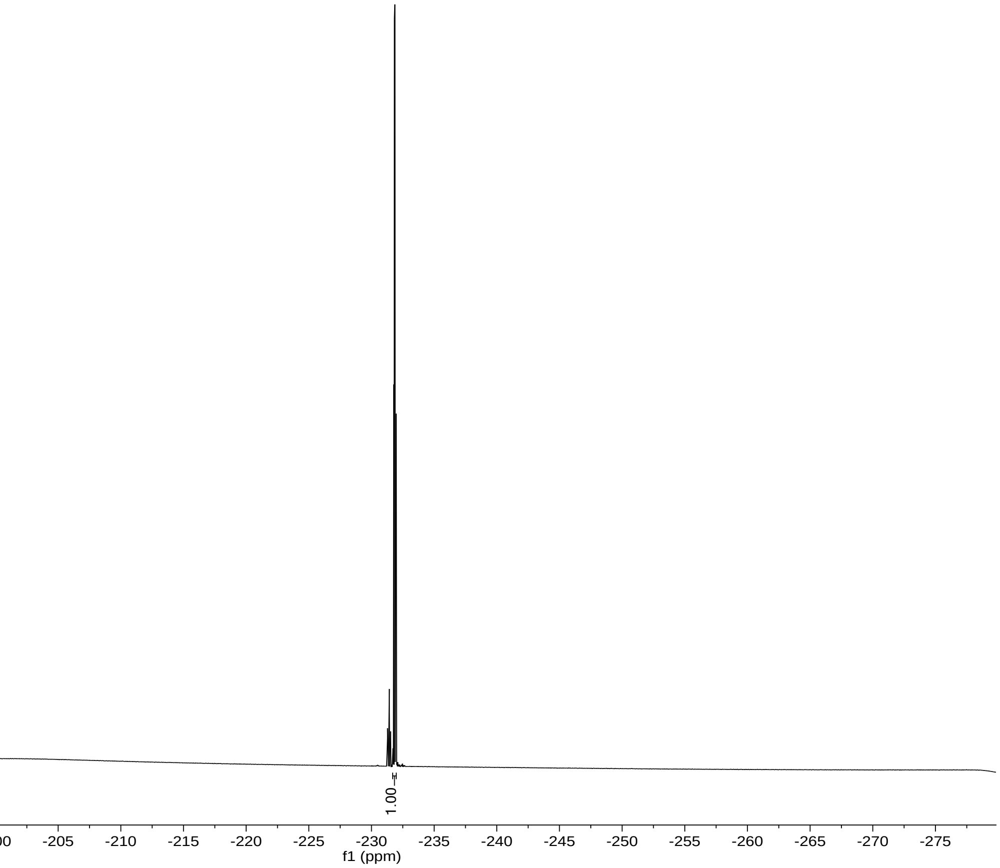
1.00
-185
-190
-195
-200
-205
-210
-215
-220
-225
-230
-235
-240
-245
-250
-255
-260
-265
-270
-275
f1 (ppm)

[EMPTY]

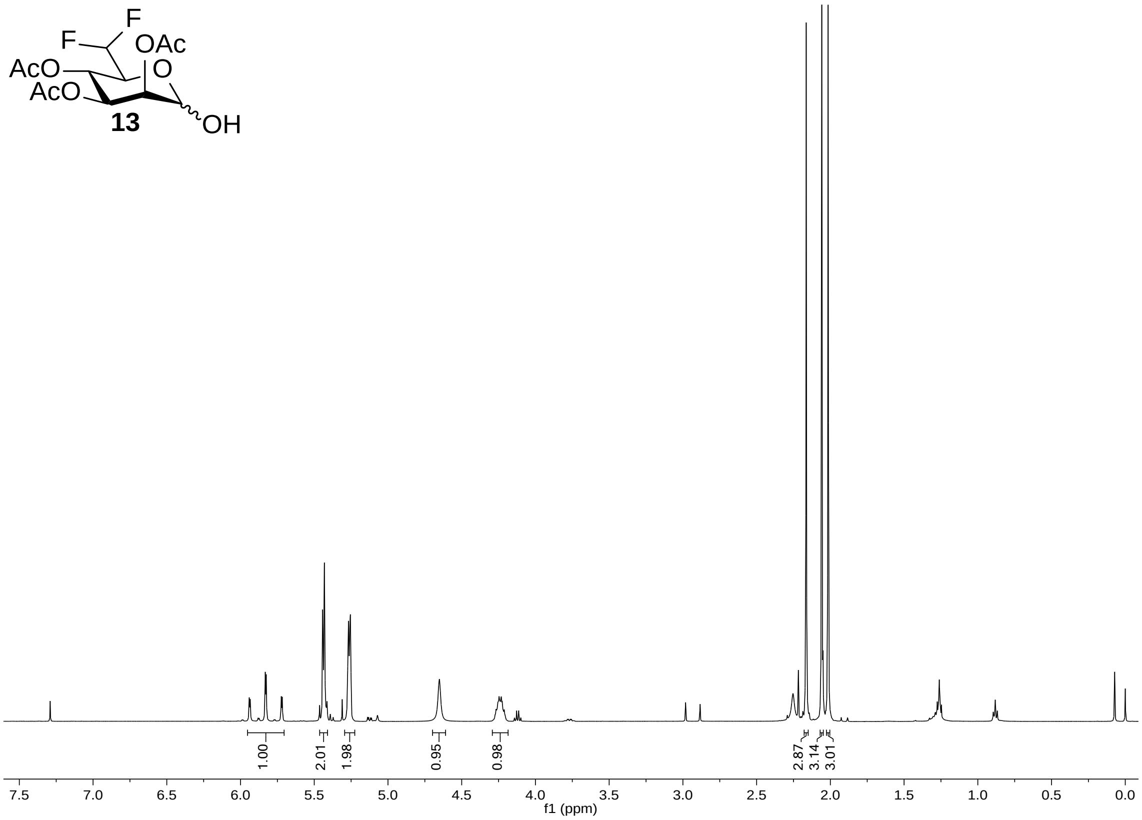
F
F
OAc
AcO
O
AcO
13
OH
1.00
2.01
1.98
0.95
0.98
2.87
3.14
3.01
7.5
7.0
6.5
6.0
5.5
5.0
4.5
4.0
3.5
3.0
2.5
2.0
1.5
1.0
0.5
0.0
f1 (ppm)

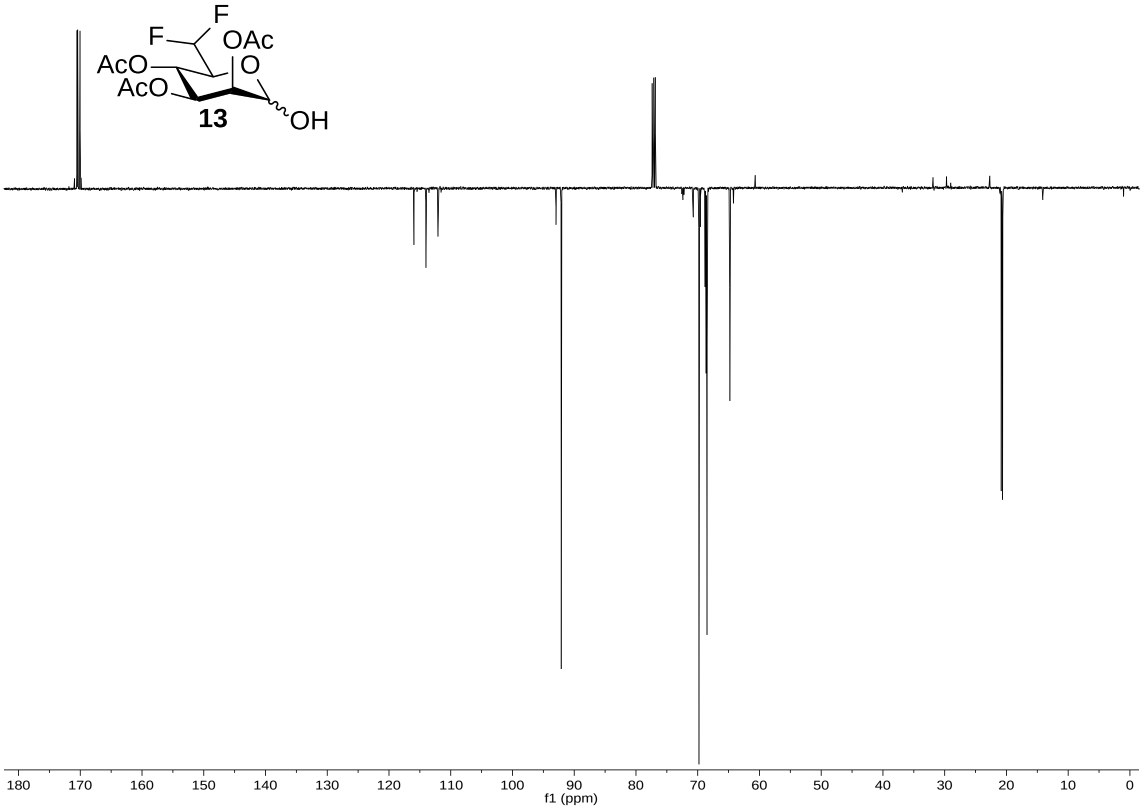
F
F
OAc
AcO
O
AcO
13
OH
180
170
160
150
140
130
120
110
100
90
80
70
60
50
40
30
20
10
0
f1 (ppm)

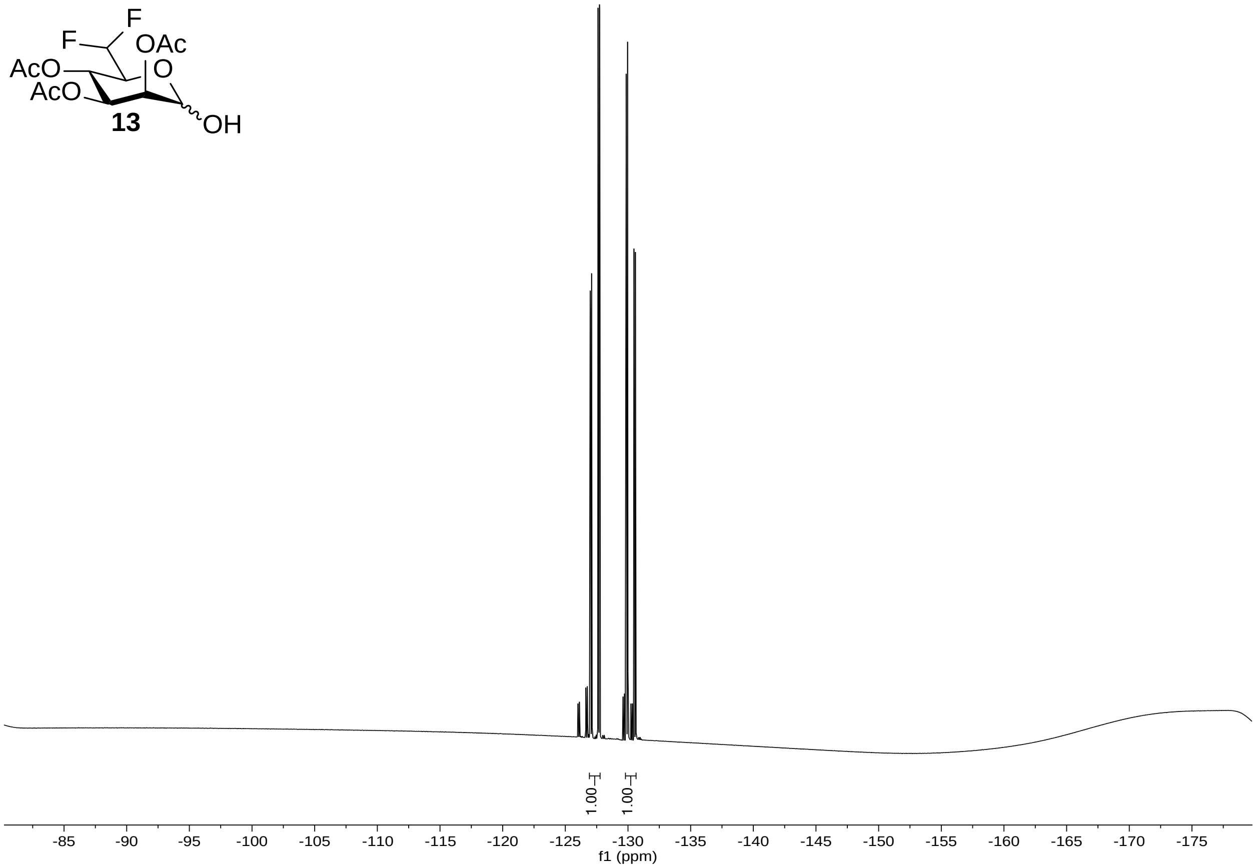
F
F
OAc
AcO
AcO
O
13
OH
1.00
1.00
-85 -90 -95 -100 -105 -110 -115 -120 -125 -130 -135 -140 -145 -150 -155 -160 -165 -170 -175
f1 (ppm)

[EMPTY]

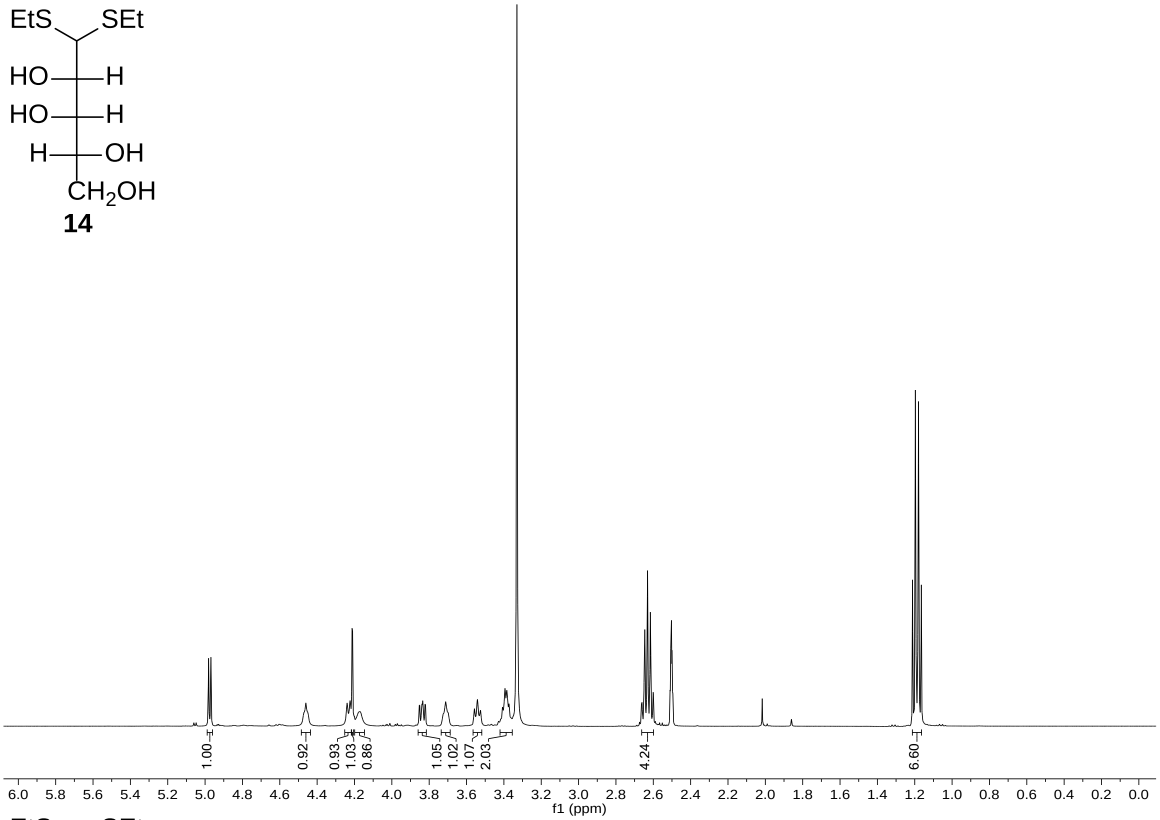
EtS SEt
HO H
HO H
H OH
CH2OH
14
1.00
0.92
0.93
1.03
0.86
1.05
1.02
1.07
2.03
4.24
6.60
6.0 5.8 5.6 5.4 5.2 5.0 4.8 4.6 4.4 4.2 4.0 3.8 3.6 3.4 3.2 3.0 2.8 2.6 2.4 2.2 2.0 1.8 1.6 1.4 1.2 1.0 0.8 0.6 0.4 0.2 0.0
f1 (ppm)

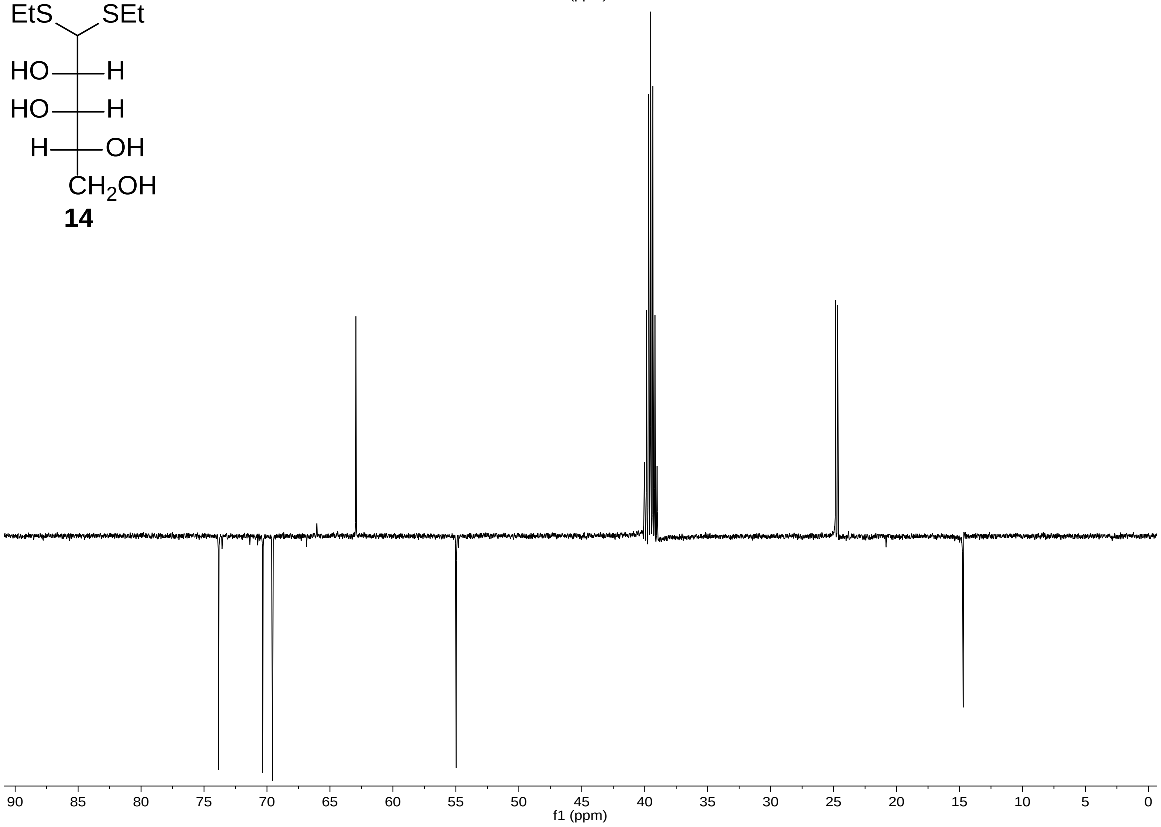
EtS SEt
HO H
HO H
H OH
CH2OH
14
90 85 80 75 70 65 60 55 50 45 40 35 30 25 20 15 10 5 0
f1 (ppm)

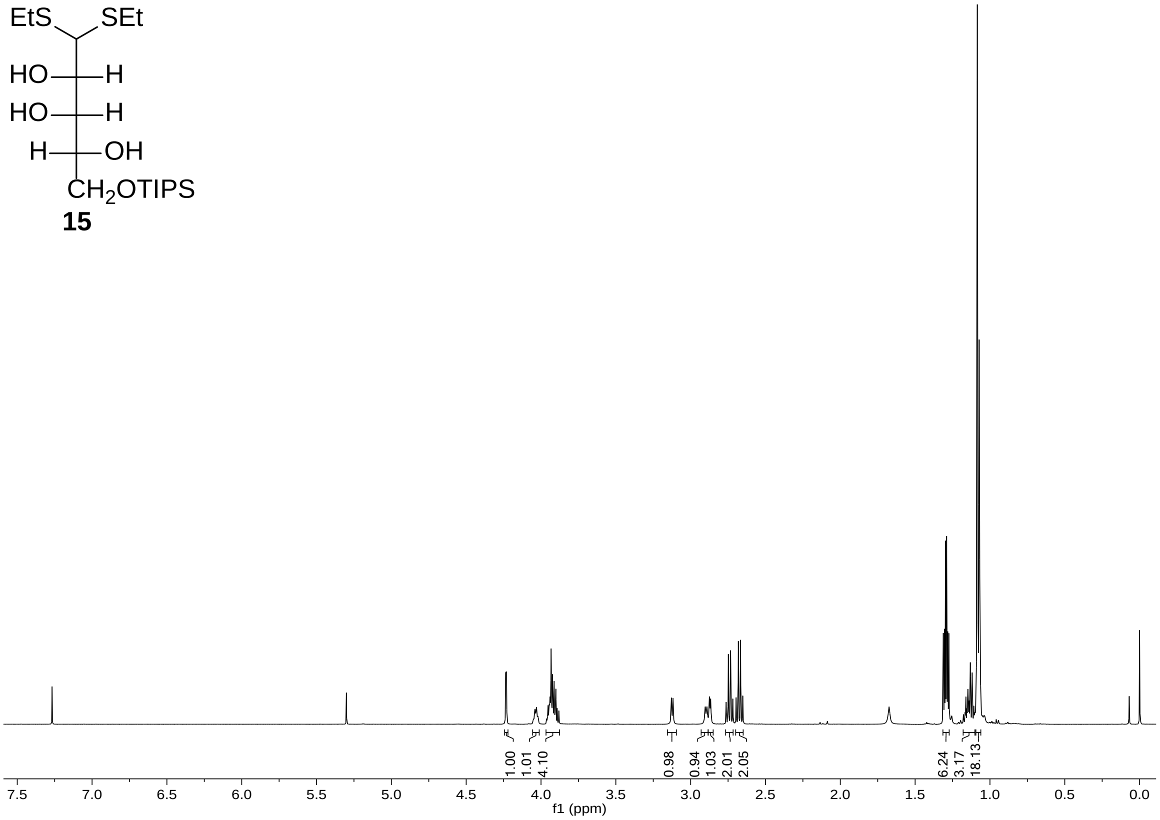
EtS
SEt
HO
H
HO
H
H
OH
CH2OTIPS
15
1.00
1.01
4.10
0.98
0.94
1.03
2.01
2.05
6.24
3.17
18.13
7.5
7.0
6.5
6.0
5.5
5.0
4.5
4.0
3.5
3.0
2.5
2.0
1.5
1.0
0.5
0.0
f1 (ppm)

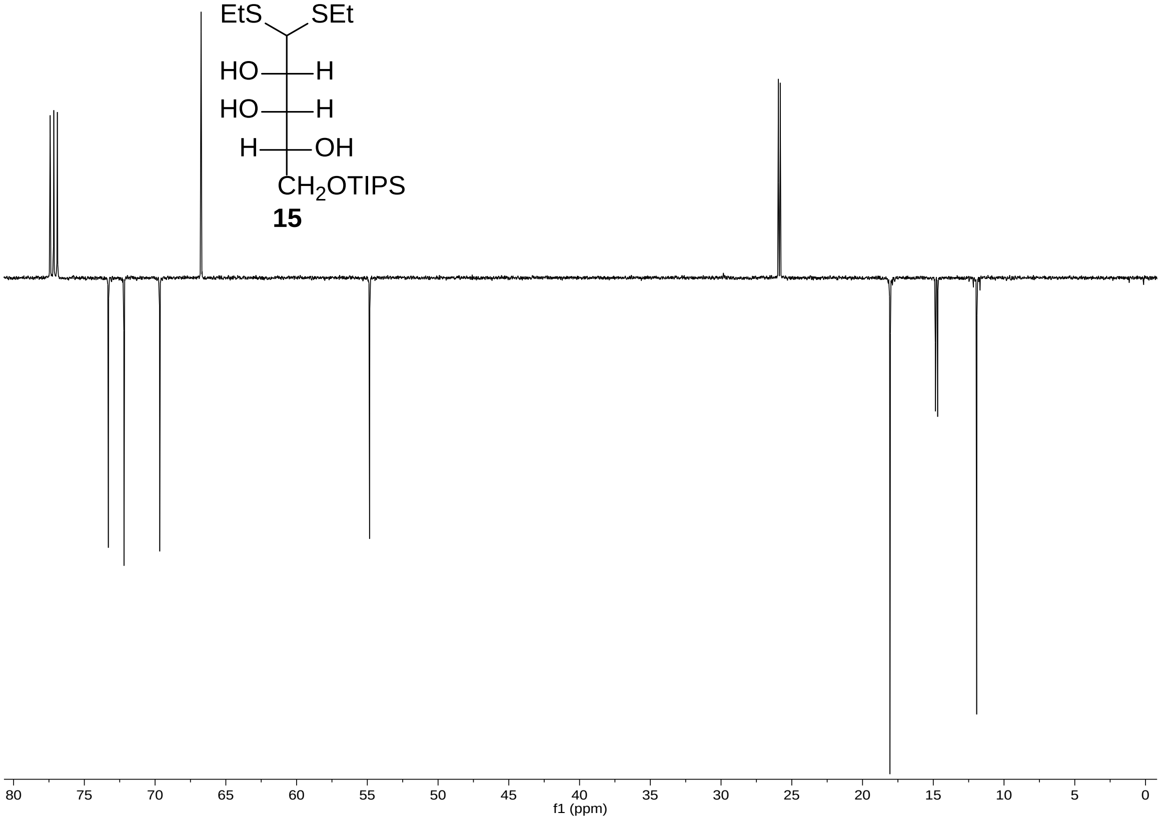
EtS
SEt
HO
H
HO
H
H
OH
CH2OTIPS
15
80
75
70
65
60
55
50
45
40
35
30
25
20
15
10
5
0
f1 (ppm)

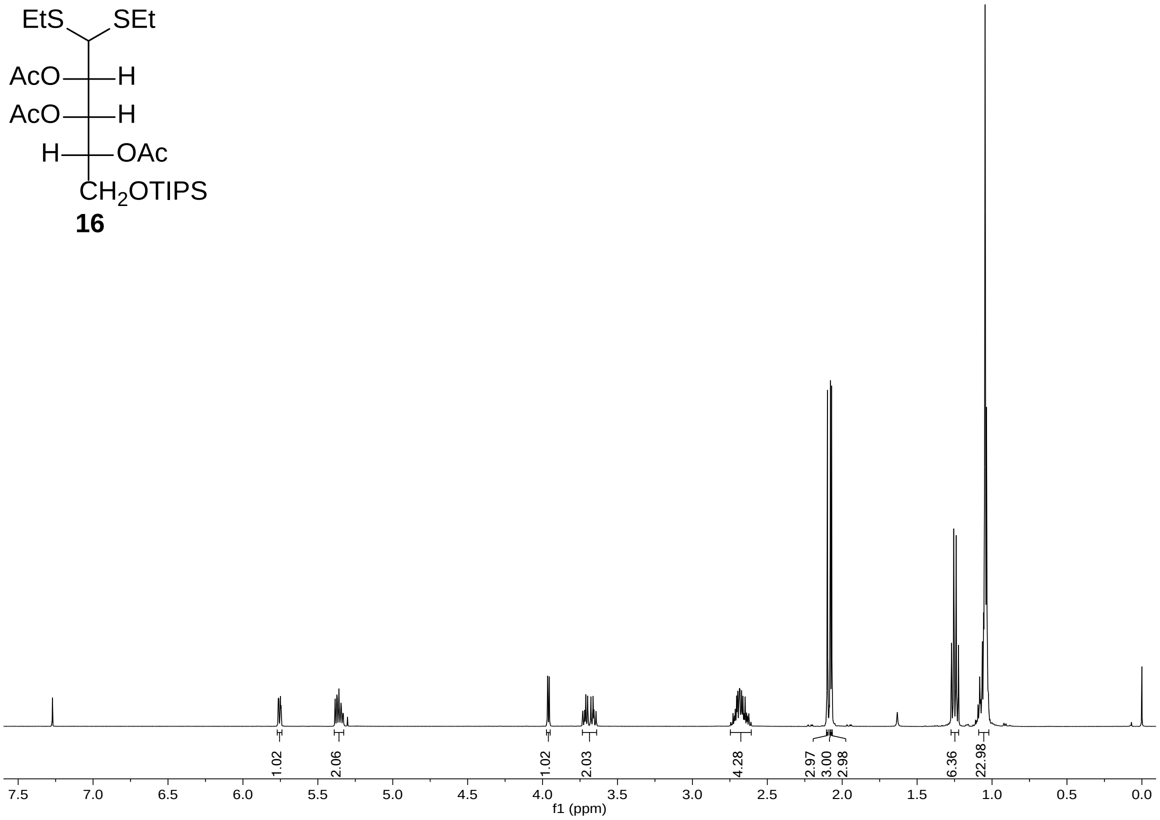
EtS
SEt
AcO
H
AcO
H
H
OAc
CH2OTIPS
16
1.02
2.06
1.02
2.03
4.28
2.97
3.00
2.98
6.36
22.98
7.5 7.0 6.5 6.0 5.5 5.0 4.5 4.0 3.5 3.0 2.5 2.0 1.5 1.0 0.5 0.0
f1 (ppm)

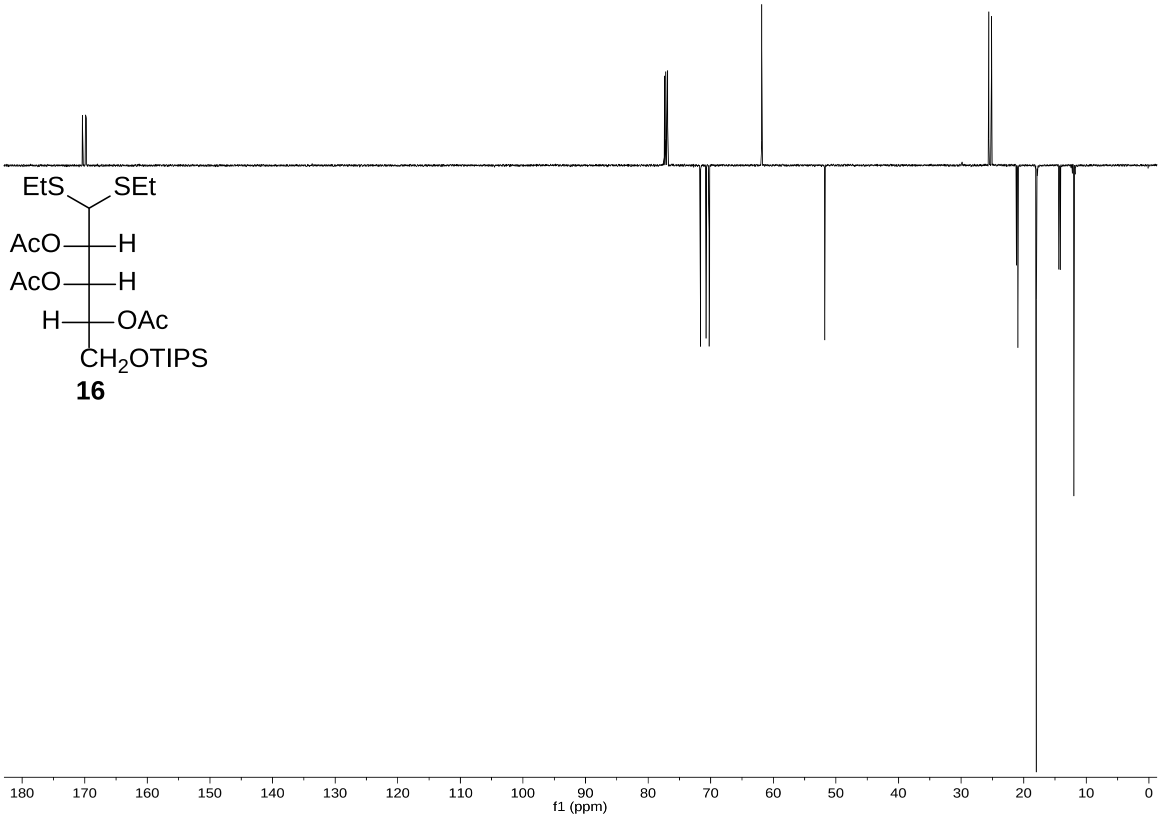
EtS
SEt
AcO
H
AcO
H
H
OAc
CH2OTIPS
16
180 170 160 150 140 130 120 110 100 90 80 70 60 50 40 30 20 10 0
f1 (ppm)

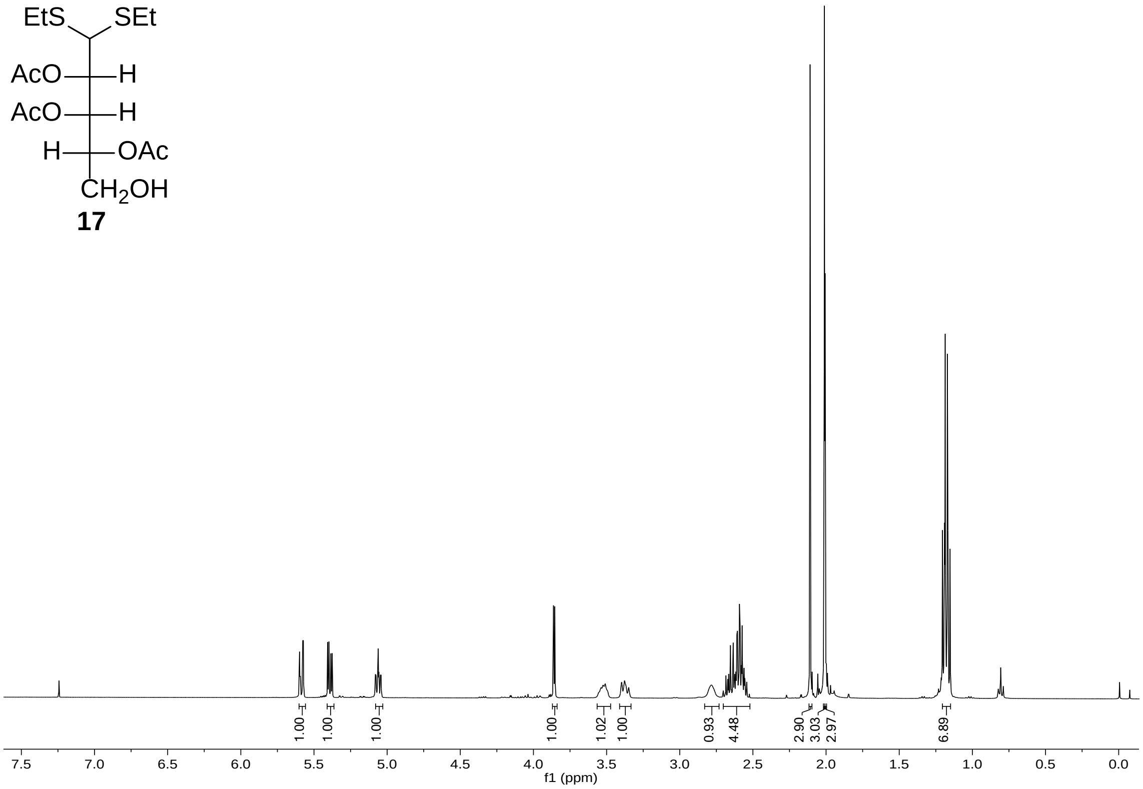
EtS SEt
AcO H
AcO H
H OAc
CH2OH
17
1.00
1.00
1.00
1.00
1.02
1.00
0.93
4.48
2.90
3.03
2.97
6.89
7.5 7.0 6.5 6.0 5.5 5.0 4.5 4.0 3.5 3.0 2.5 2.0 1.5 1.0 0.5 0.0
f1 (ppm)

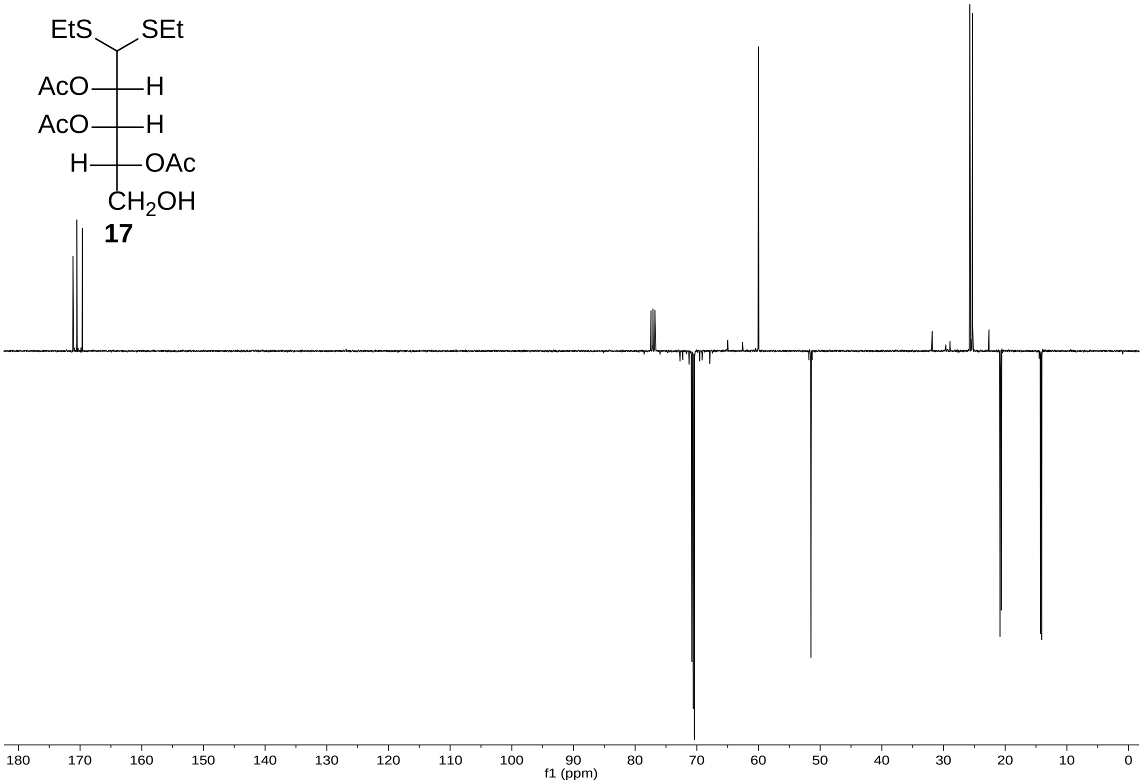
EtS SEt
AcO H
AcO H
H OAc
CH2OH
17
180 170 160 150 140 130 120 110 100 90 80 70 60 50 40 30 20 10 0
f1 (ppm)

Fluorinated rhamnosides inhibit cellular fucosylation.
Supporting information

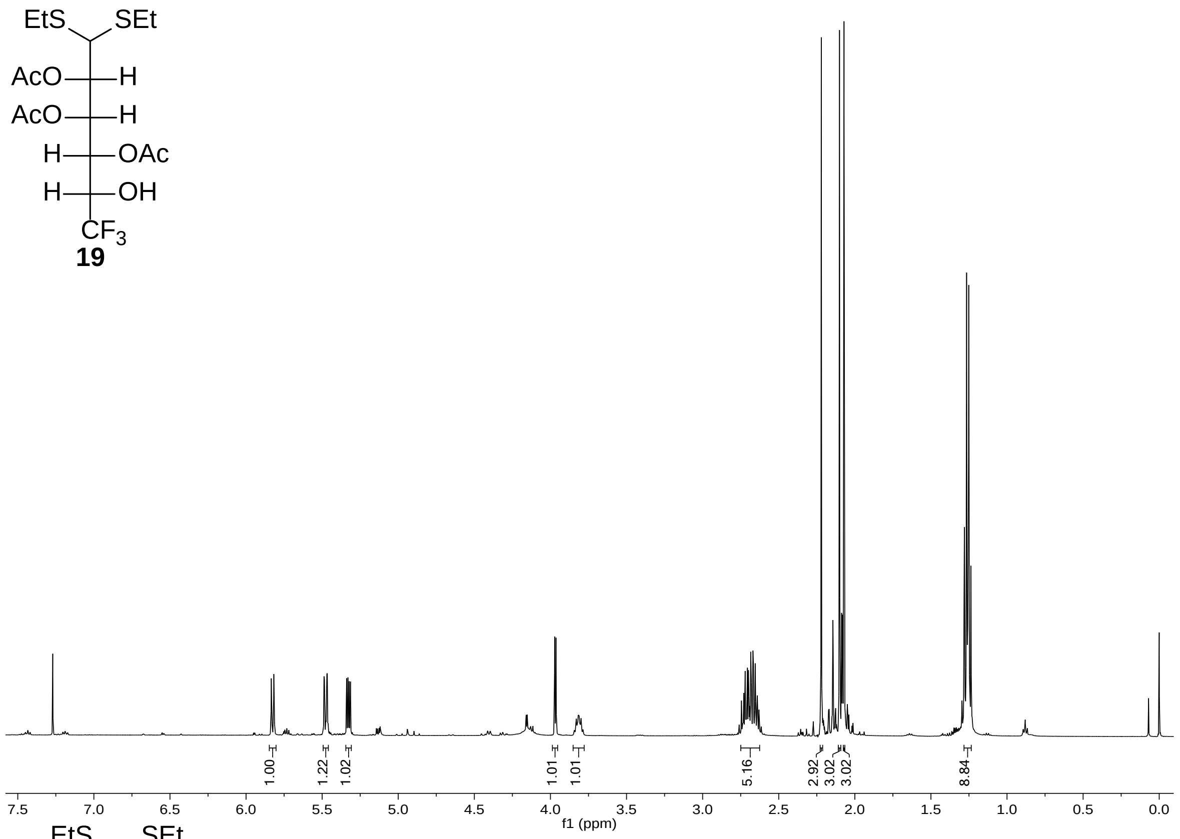

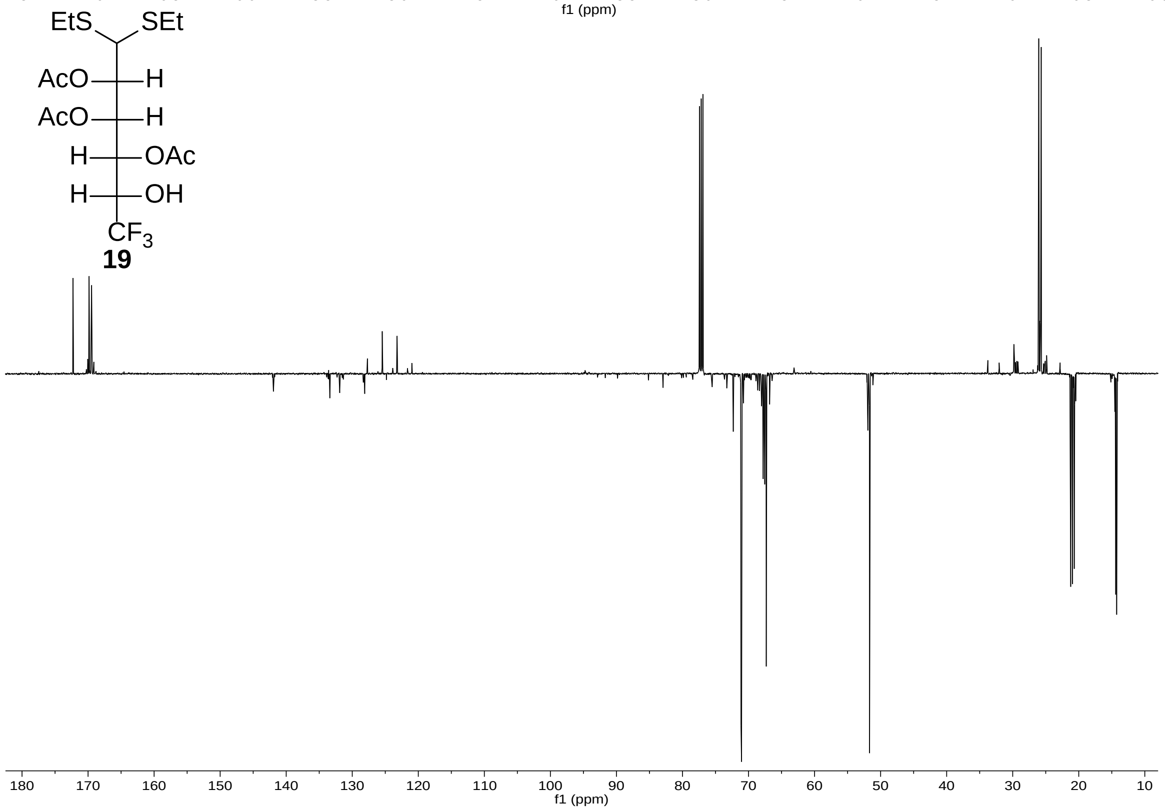

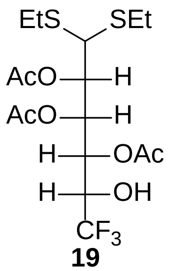
19

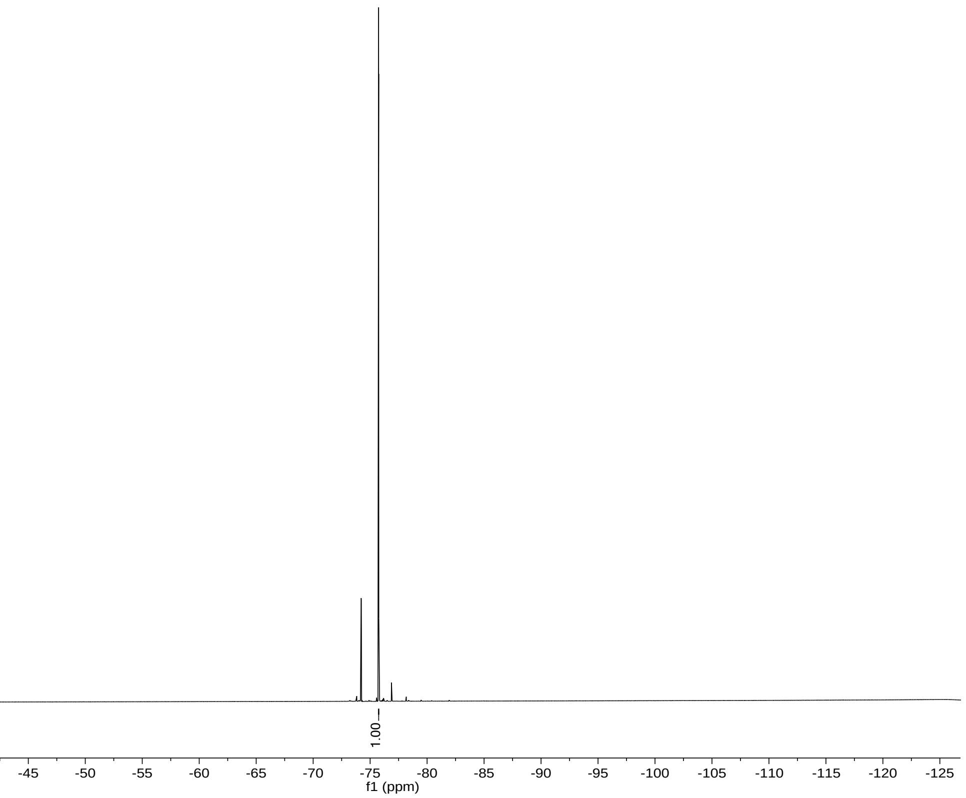
1.00
-30
-35
-40
-45
-50
-55
-60
-65
-70
-75
-80
-85
-90
-95
-100
-105
-110
-115
-120
-125
f1 (ppm)

[EMPTY]

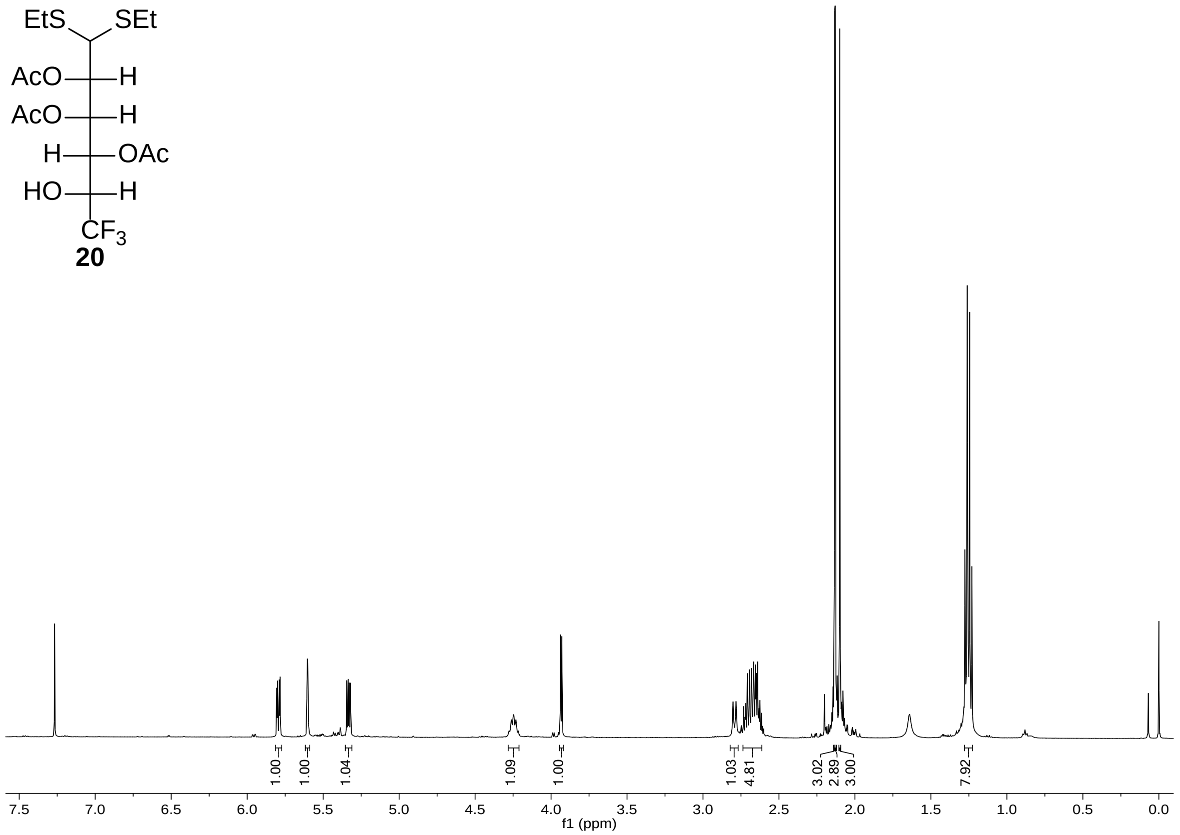
EtS
SEt
AcO
H
AcO
H
H
OAc
HO
H
CF3
20
1.00
1.00
1.04
1.09
1.00
1.03
4.81
3.02
2.89
3.00
7.92
7.5 7.0 6.5 6.0 5.5 5.0 4.5 4.0 3.5 3.0 2.5 2.0 1.5 1.0 0.5 0.0
f1 (ppm)

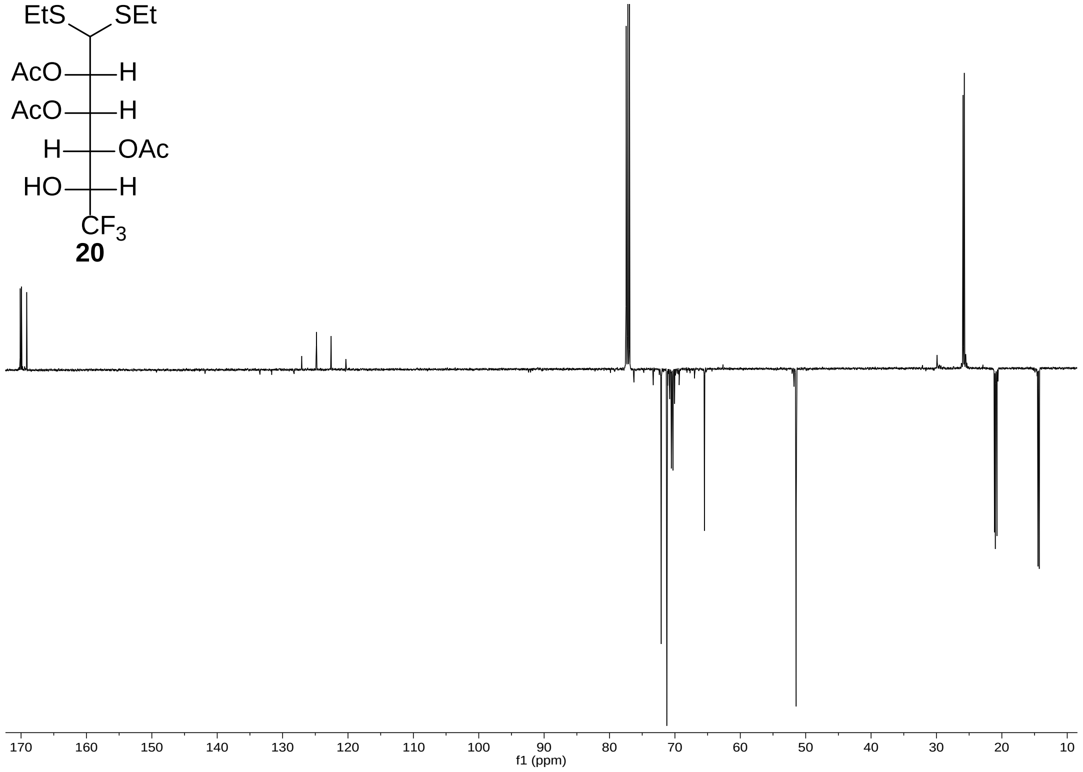
EtS
SEt
AcO
H
AcO
H
H
OAc
HO
H
CF3
20
170 160 150 140 130 120 110 100 90 80 70 60 50 40 30 20 10
f1 (ppm)

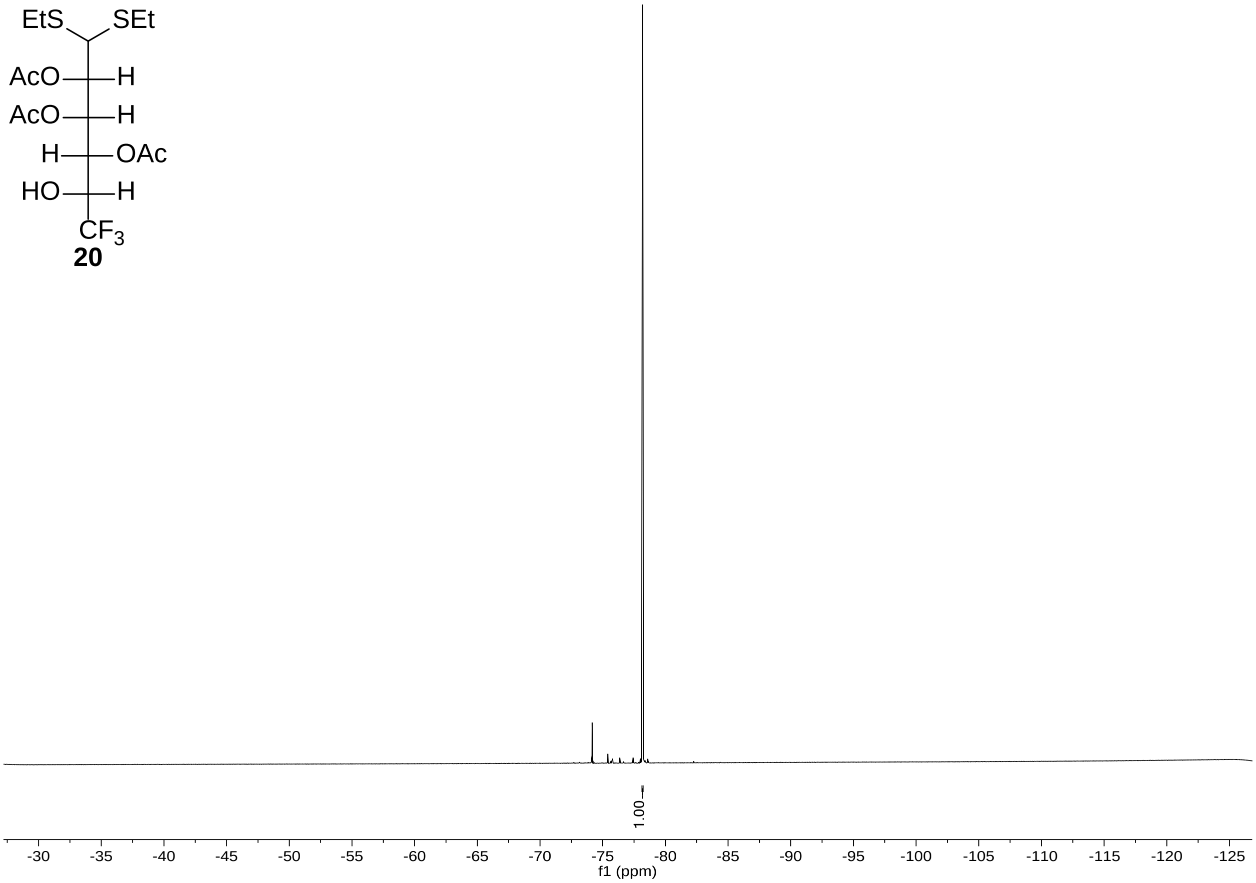
EtS
SEt
AcO
H
AcO
H
H
OAc
HO
H
CF3
1.00
-30
-35
-40
-45
-50
-55
-60
-65
-70
-75
-80
-85
-90
-95
-100
-105
-110
-115
-120
-125
f1 (ppm)

20

[EMPTY]

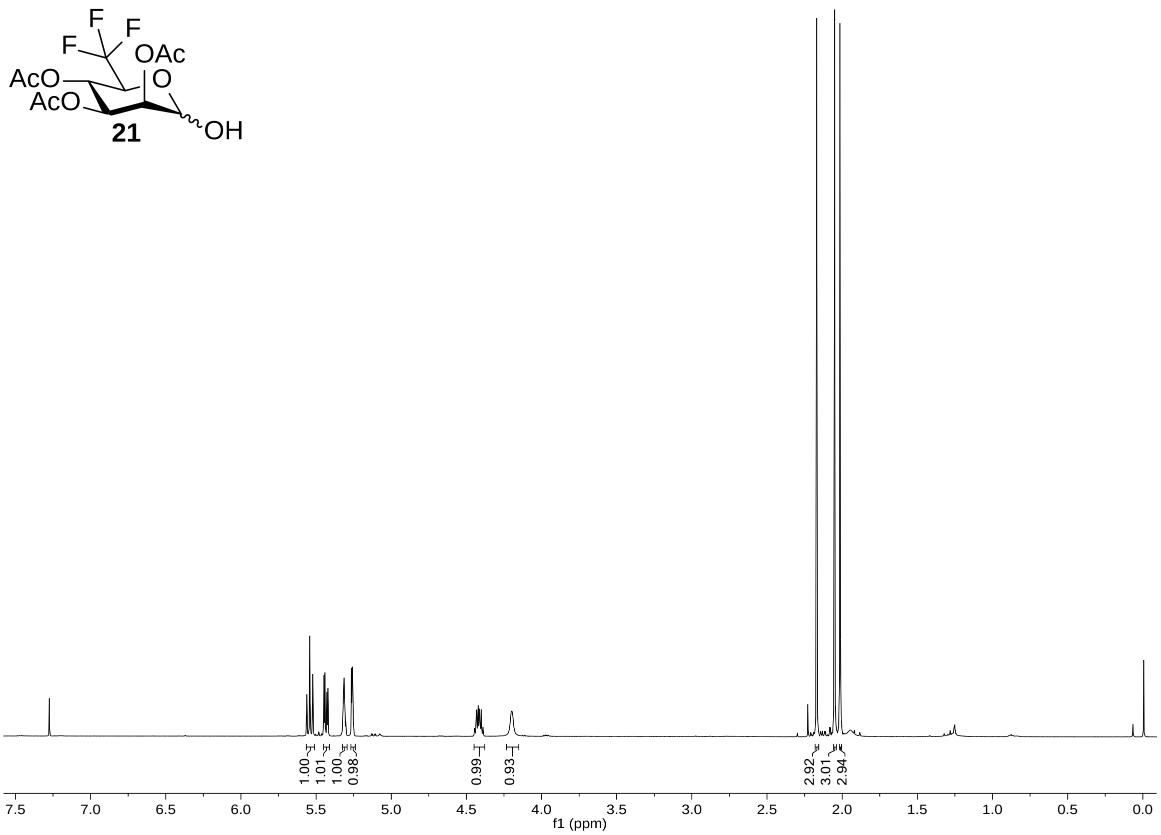
F
F
F
OAc
AcO
AcO
O
21
OH
1.00
1.01
1.00
0.98
0.99
0.93
2.92
3.01
2.94
f1 (ppm)

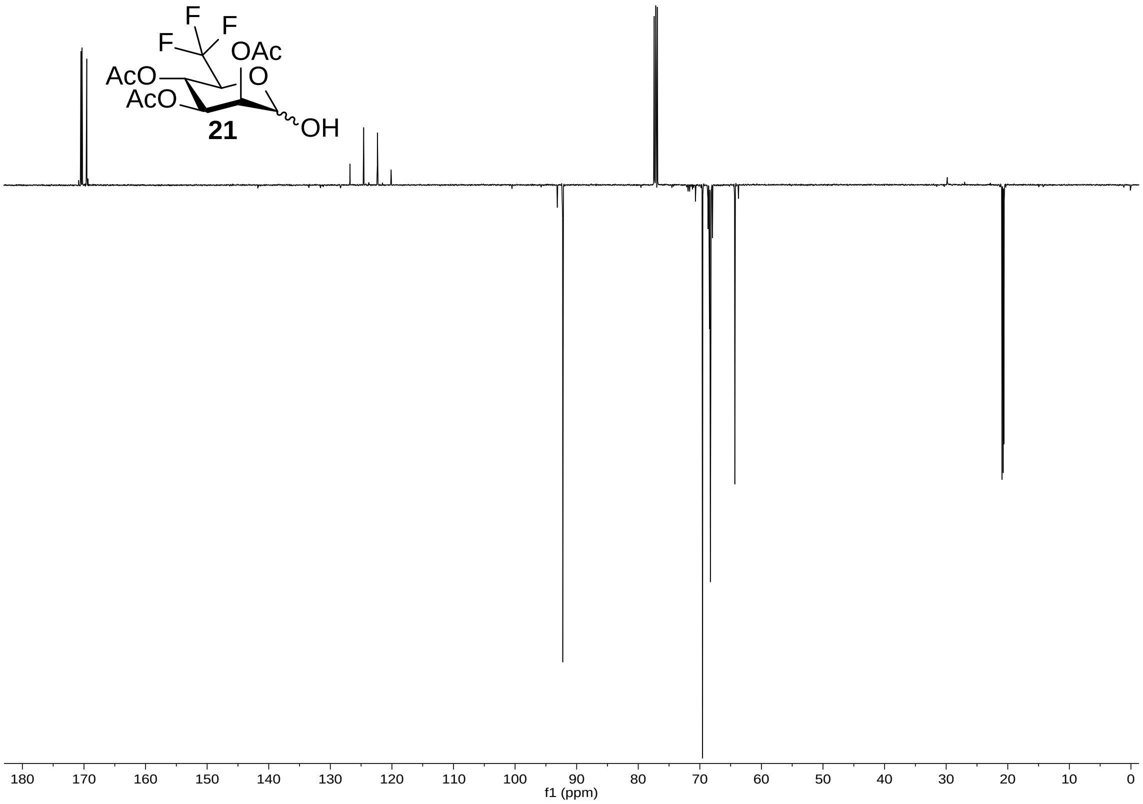
F
F
F
OAc
AcO
AcO
O
21
OH
f1 (ppm)

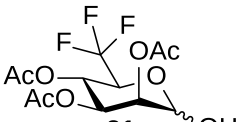
F
F
F
OAc
AcO
O
AcO
OH

21

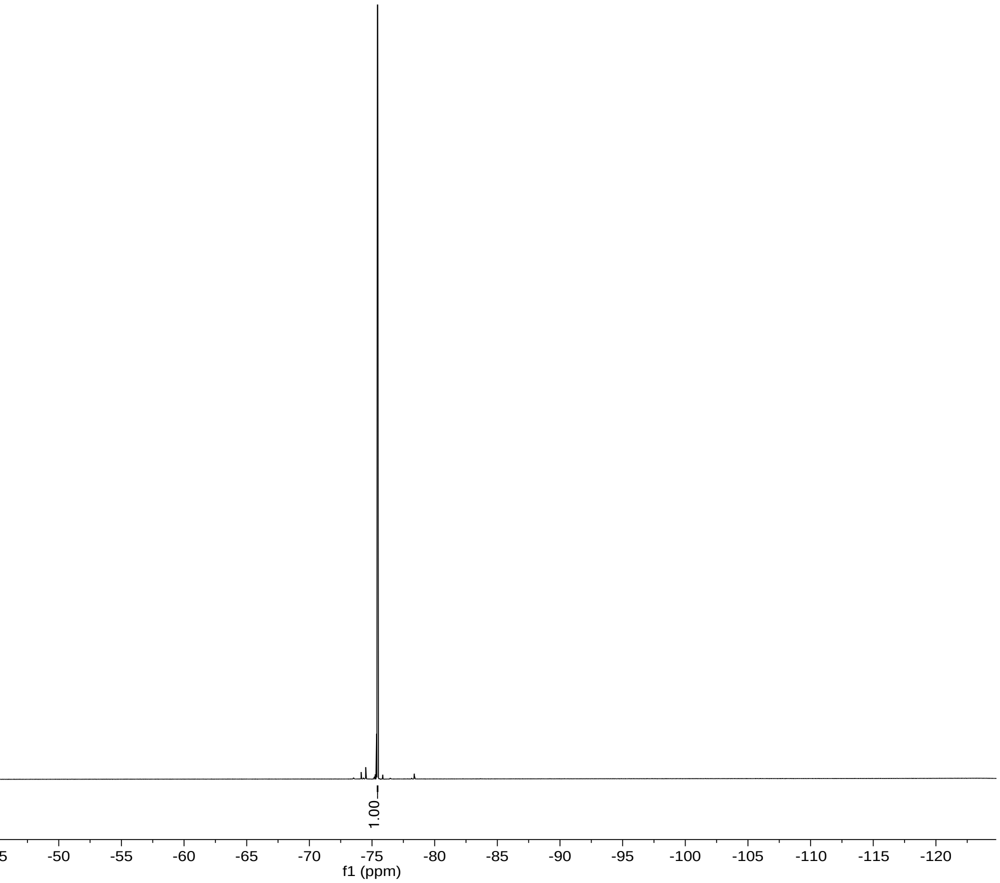
1.00
-30
-35
-40
-45
-50
-55
-60
-65
-70
-75
-80
-85
-90
-95
-100
-105
-110
-115
-120
f1 (ppm)

[EMPTY]

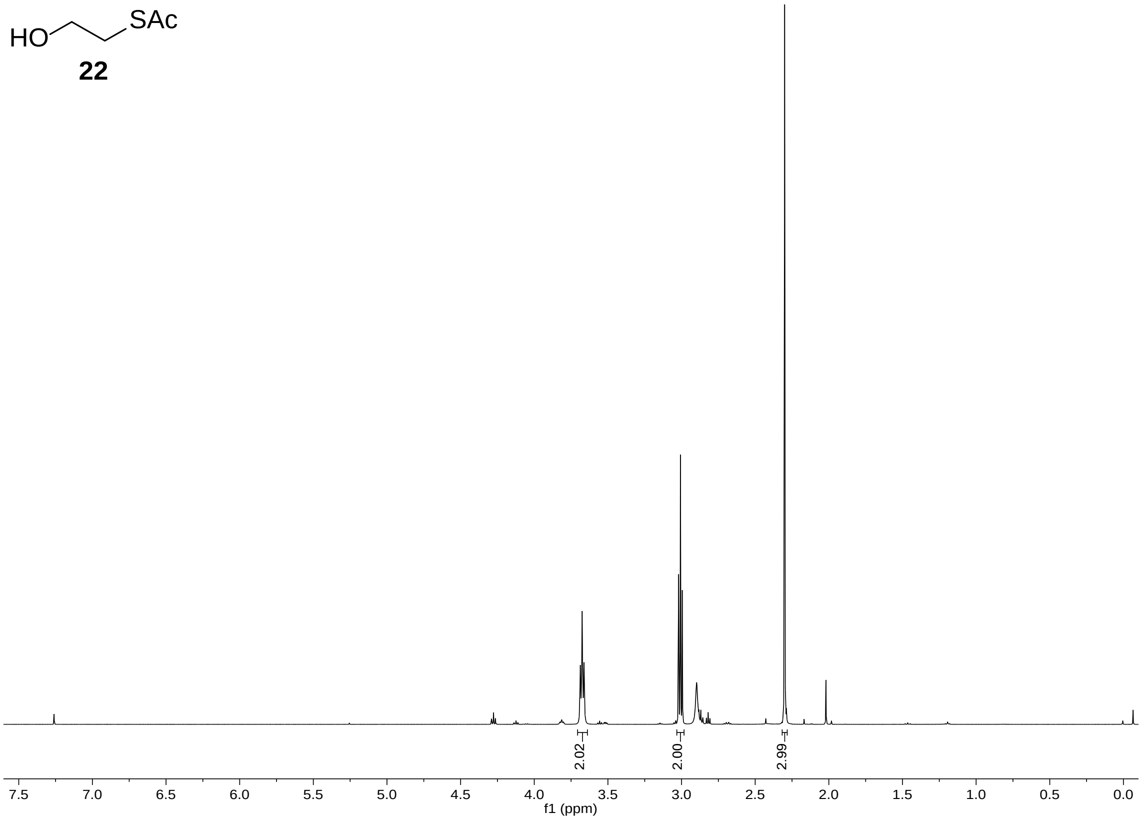
HO
SAc
22
2.02
2.00
2.99
7.5 7.0 6.5 6.0 5.5 5.0 4.5 4.0 3.5 3.0 2.5 2.0 1.5 1.0 0.5 0.0
f1 (ppm)

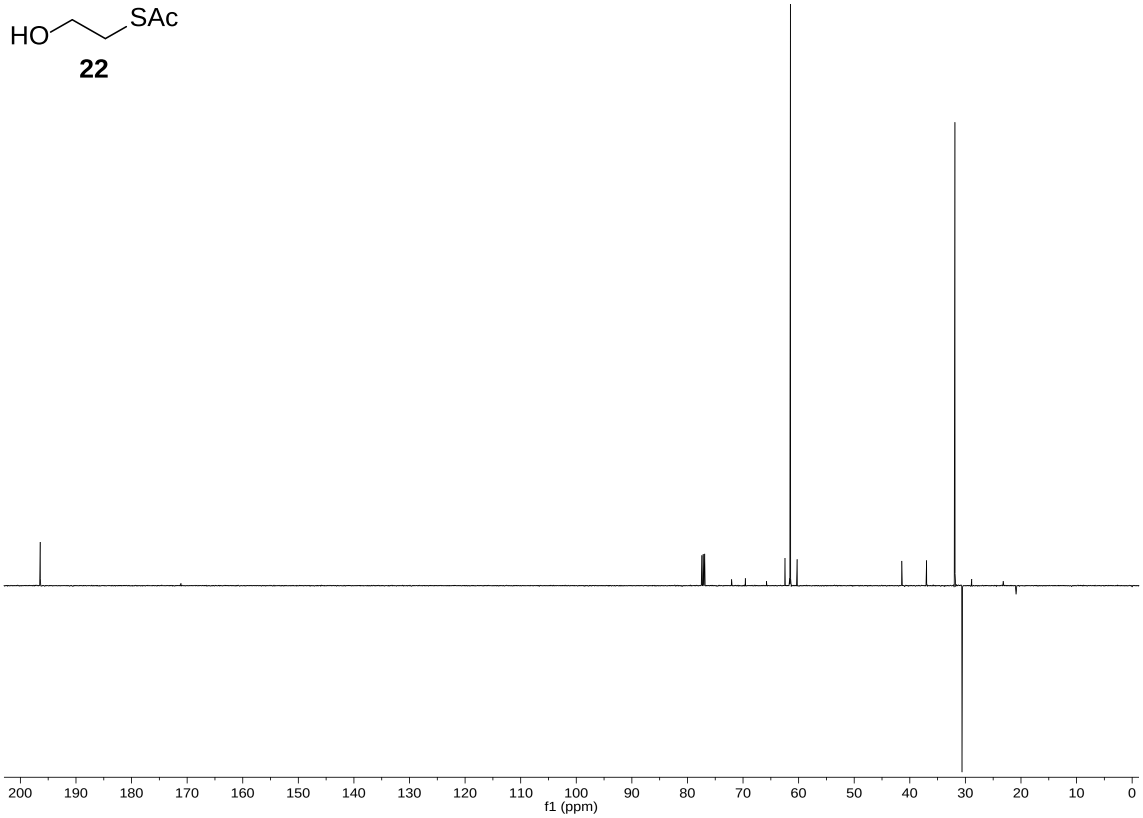
HO
SAc
22
200 190 180 170 160 150 140 130 120 110 100 90 80 70 60 50 40 30 20 10 0
f1 (ppm)

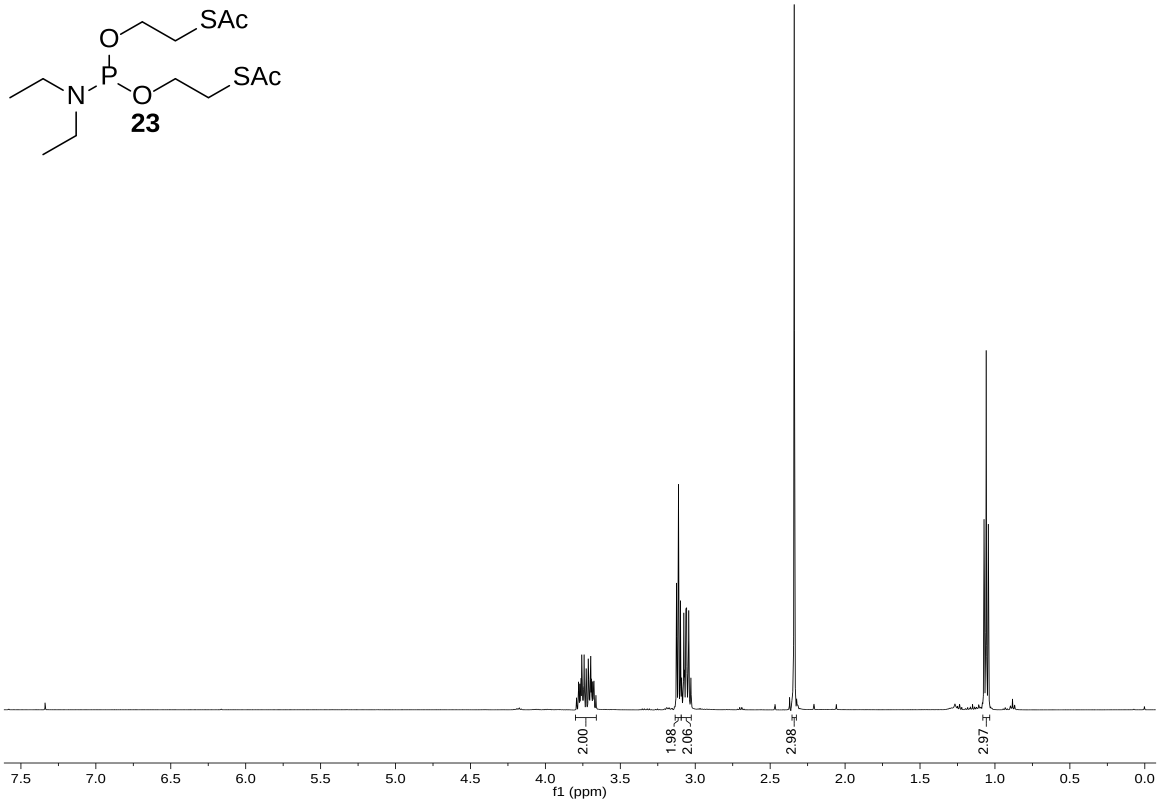
SAc
O
P
N
O
SAc
23
2.00
1.98
2.06
2.98
2.97
7.5 7.0 6.5 6.0 5.5 5.0 4.5 4.0 3.5 3.0 2.5 2.0 1.5 1.0 0.5 0.0
f1 (ppm)

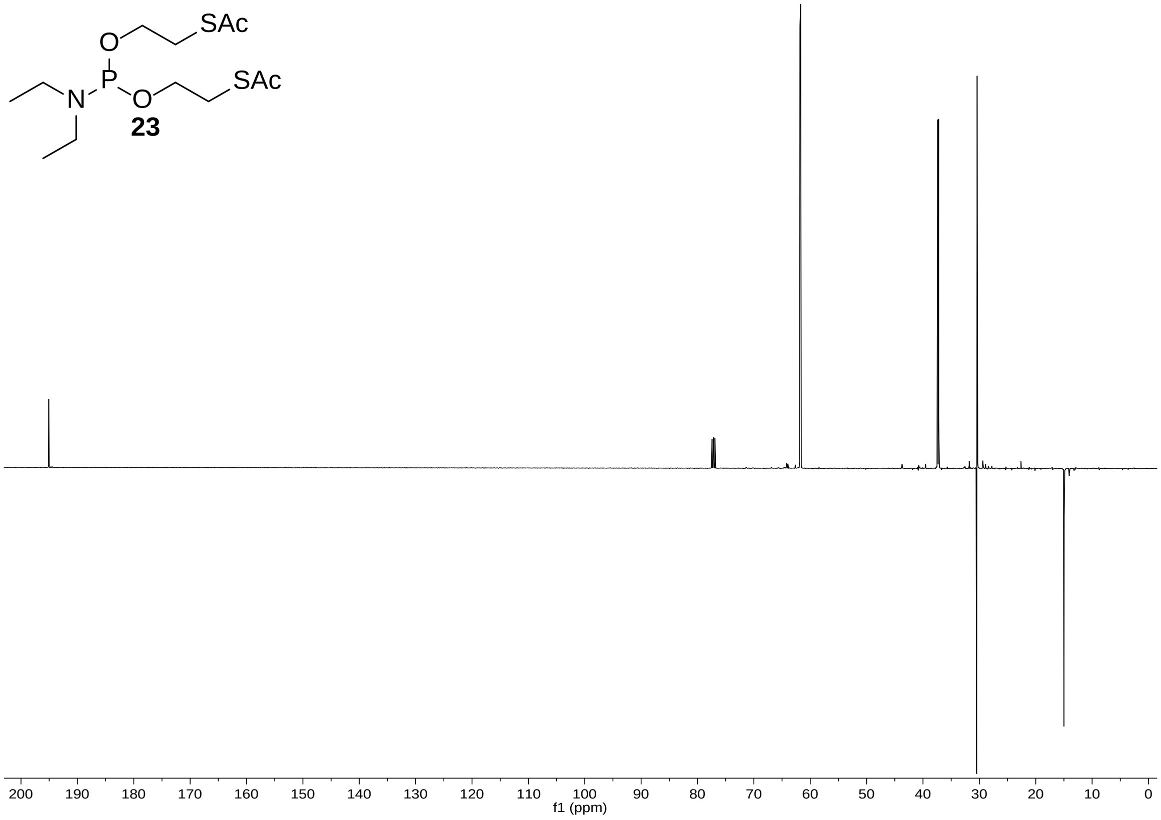
SAc
O
P
N
O
SAc
23
200 190 180 170 160 150 140 130 120 110 100 90 80 70 60 50 40 30 20 10 0
f1 (ppm)

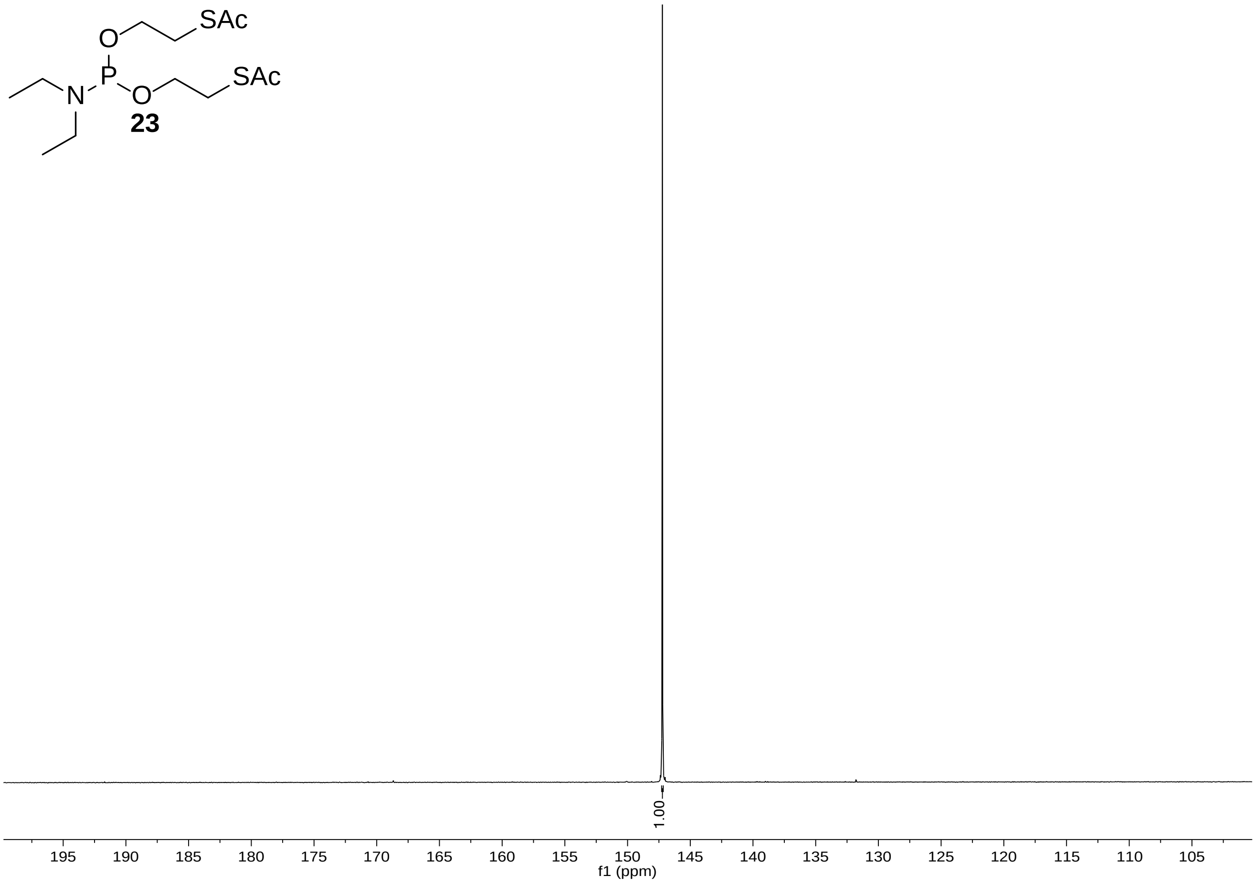
SAc
SAc
O
P
N
O
23
1.00
195 190 185 180 175 170 165 160 155 150 145 140 135 130 125 120 115 110 105
f1 (ppm)

[EMPTY]

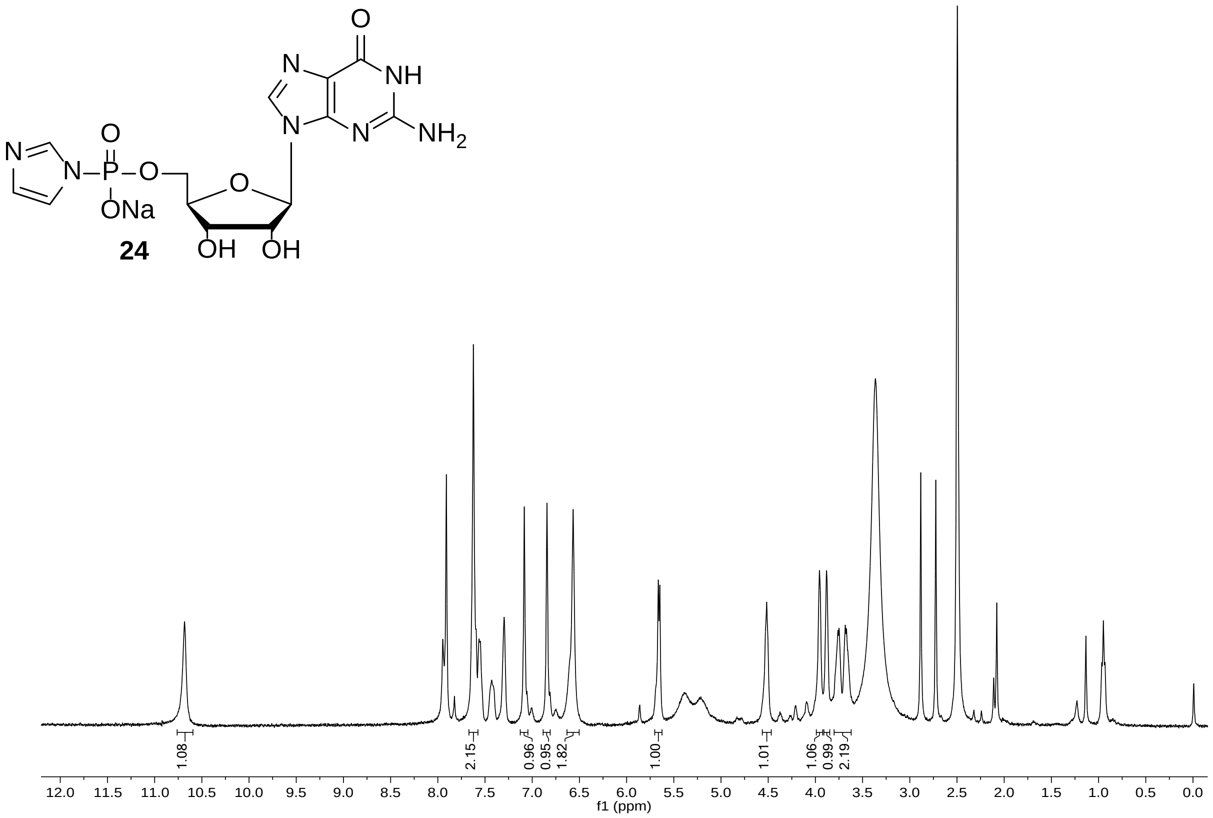

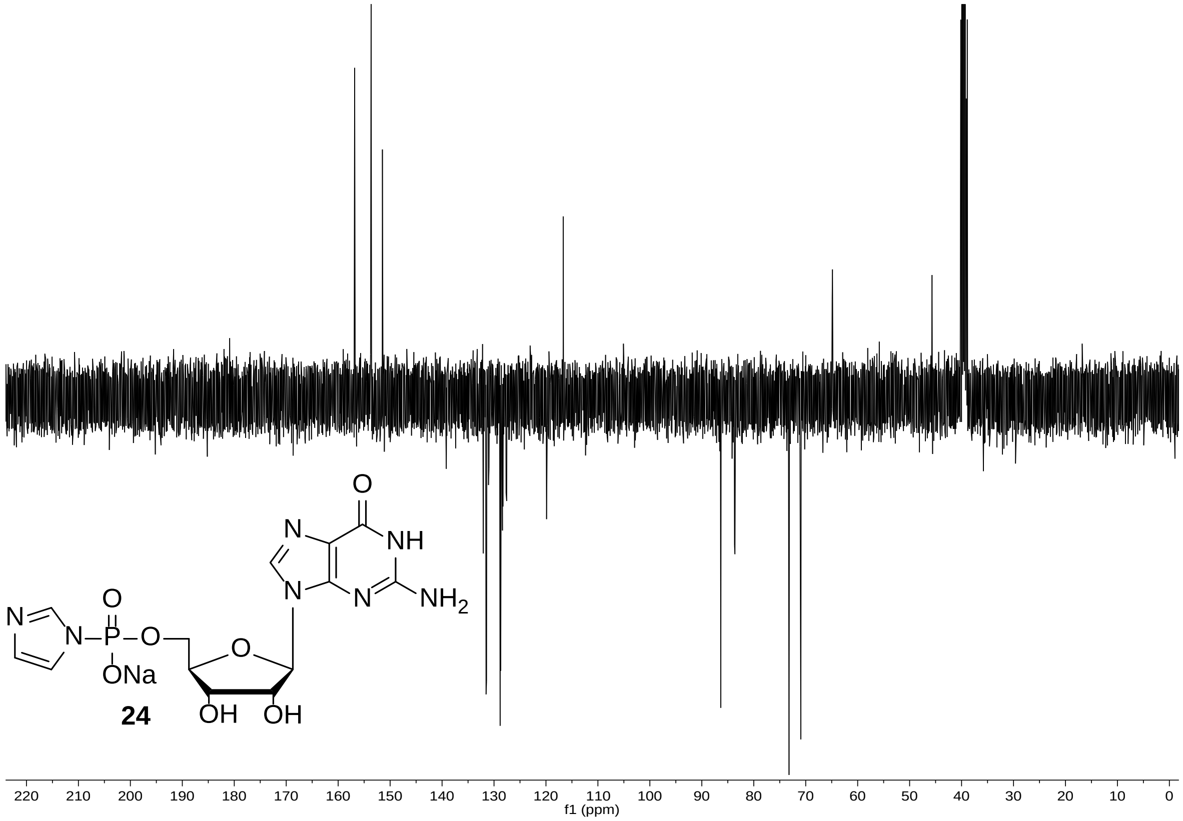

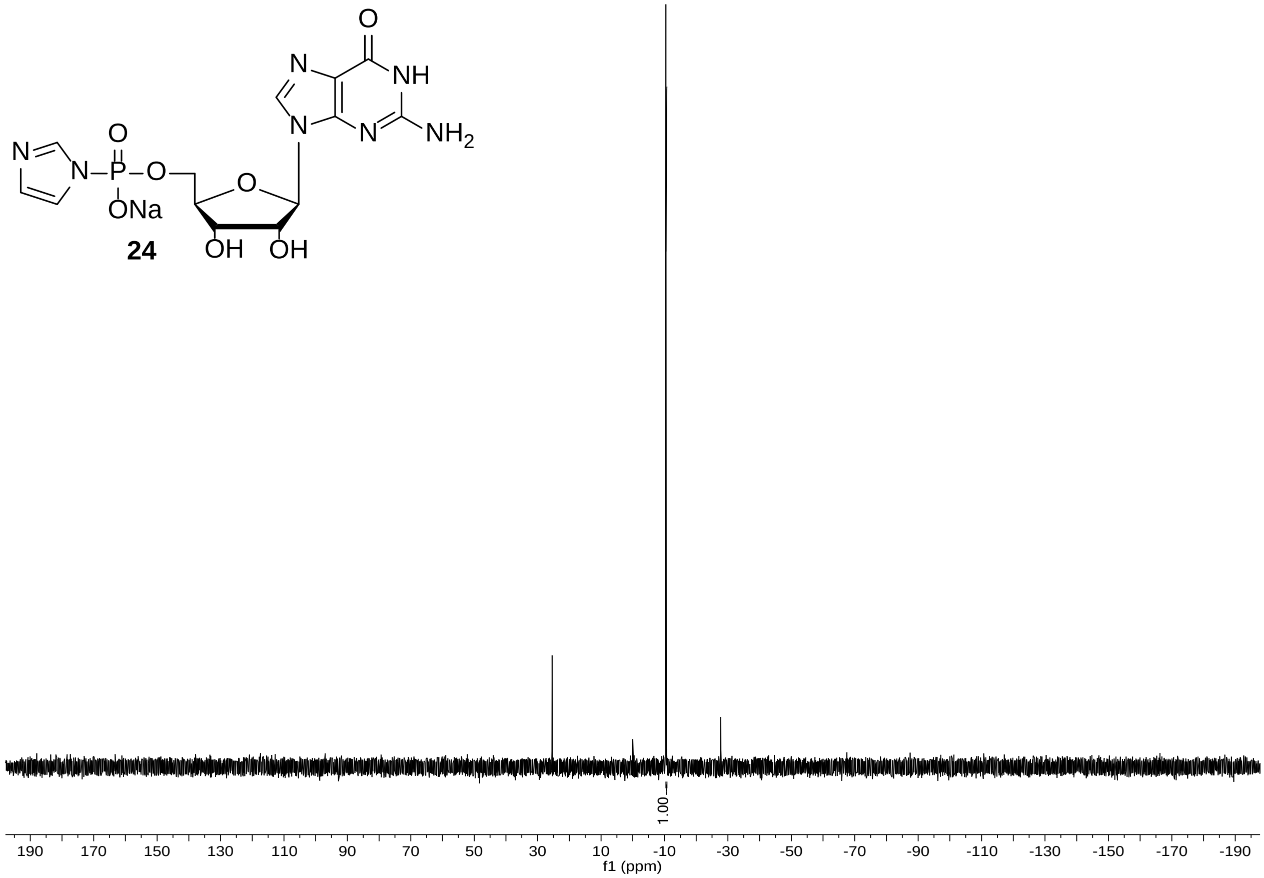
24
1.00
190 170 150 130 110 90 70 50 30 10 -10 -30 -50 -70 -90 -110 -130 -150 -170 -190
f1 (ppm)

[EMPTY]

Fluorinated rhamnosides inhibit cellular fucosylation.
Supporting information

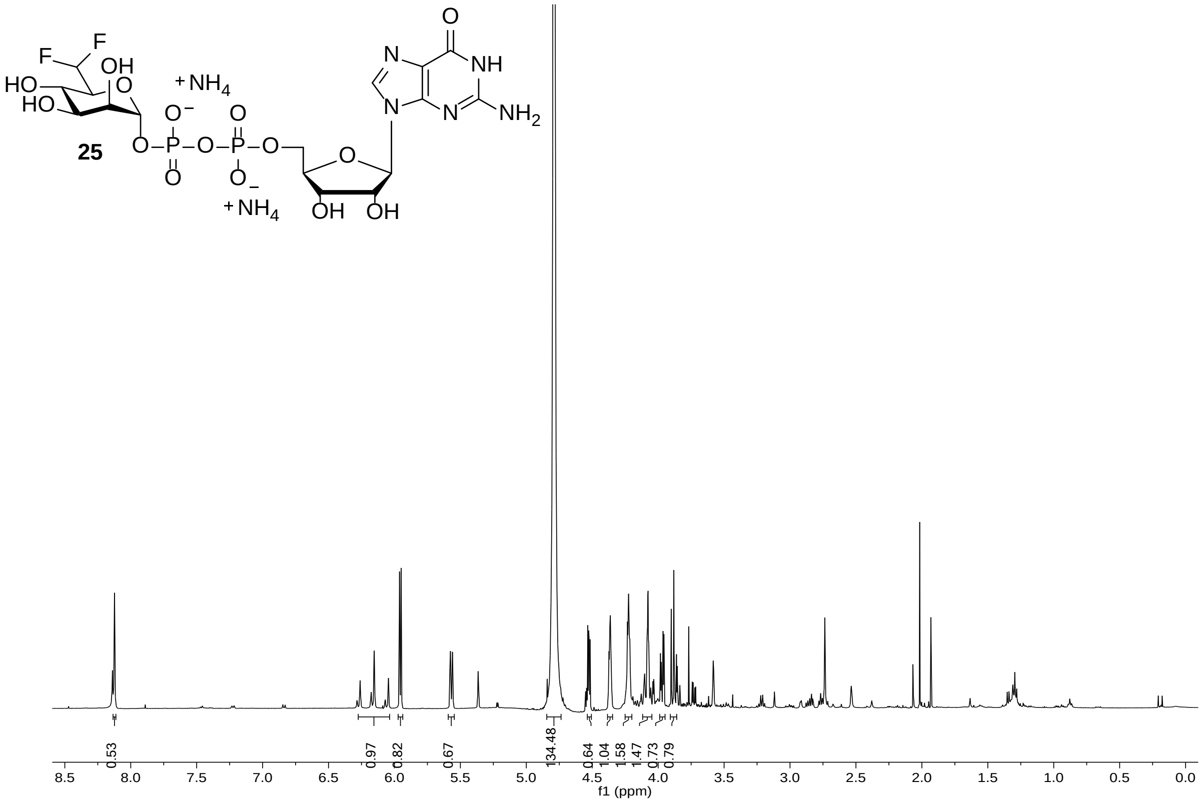

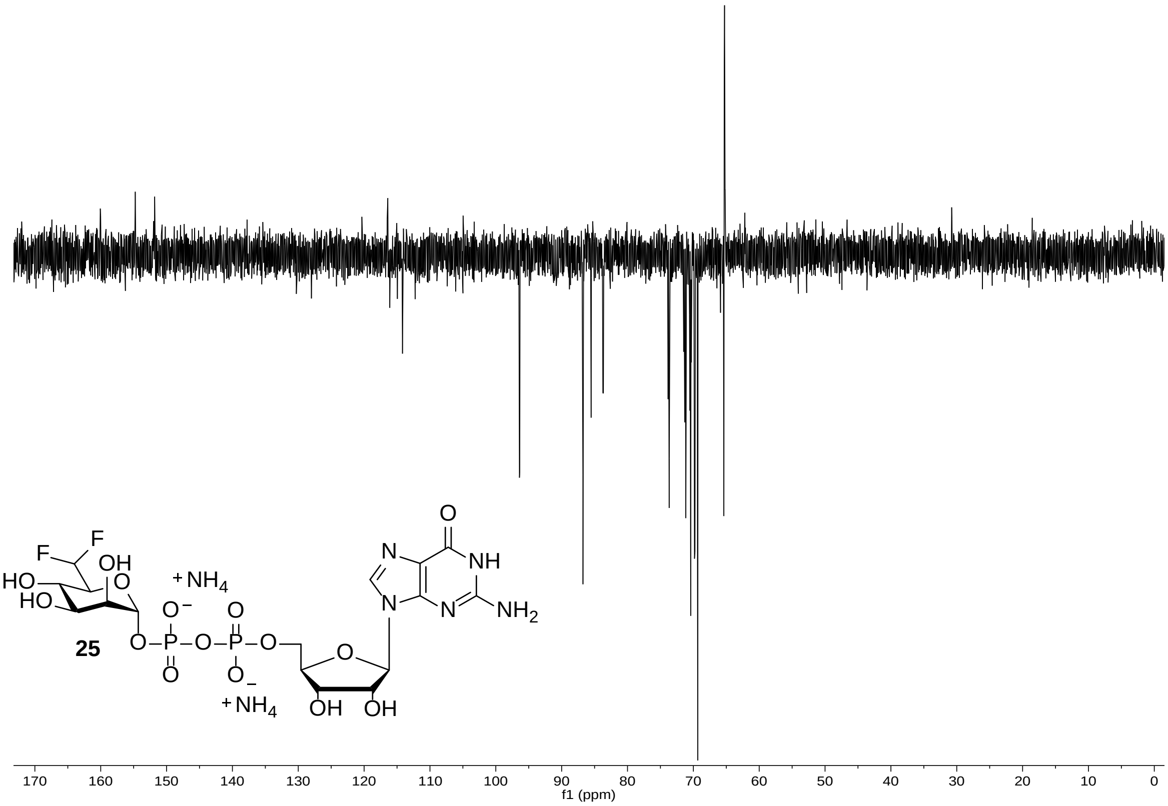

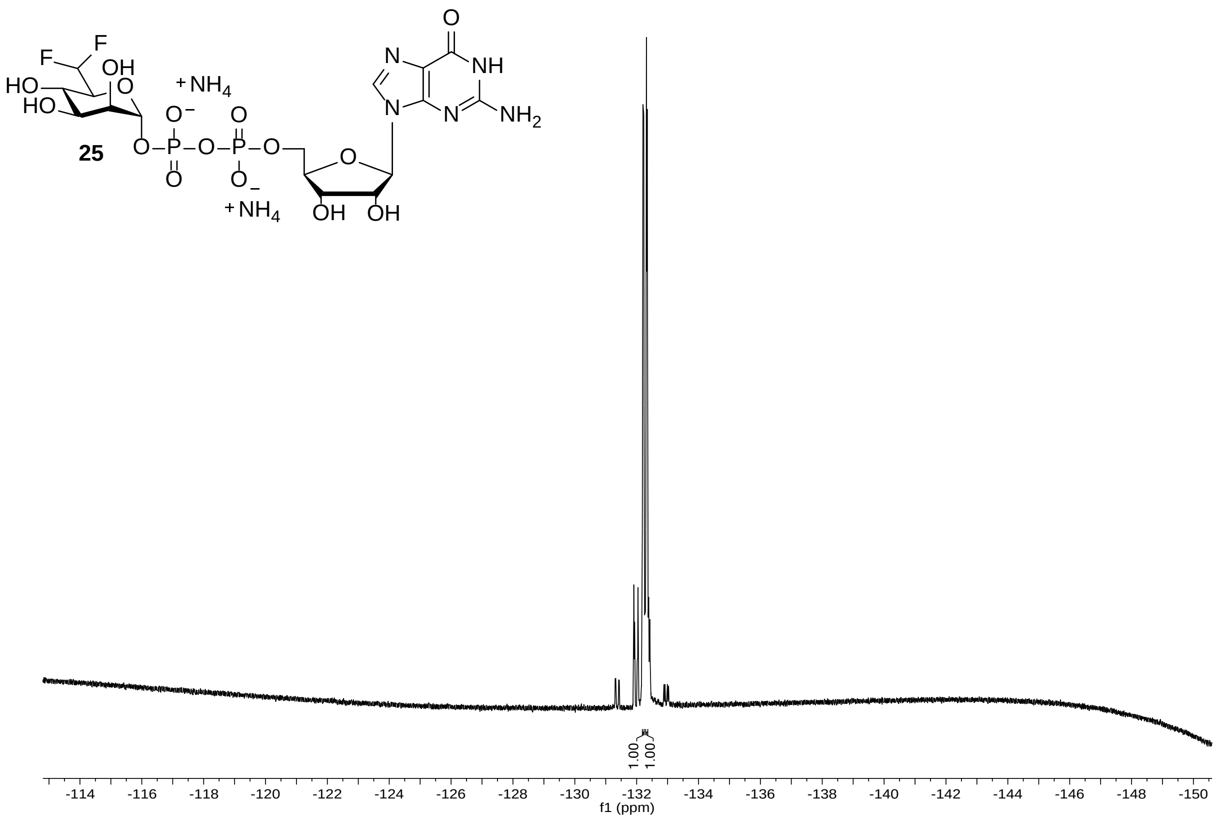
25
1.00
1.00
-114 -116 -118 -120 -122 -124 -126 -128 -130 -132 -134 -136 -138 -140 -142 -144 -146 -148 -150
f1 (ppm)

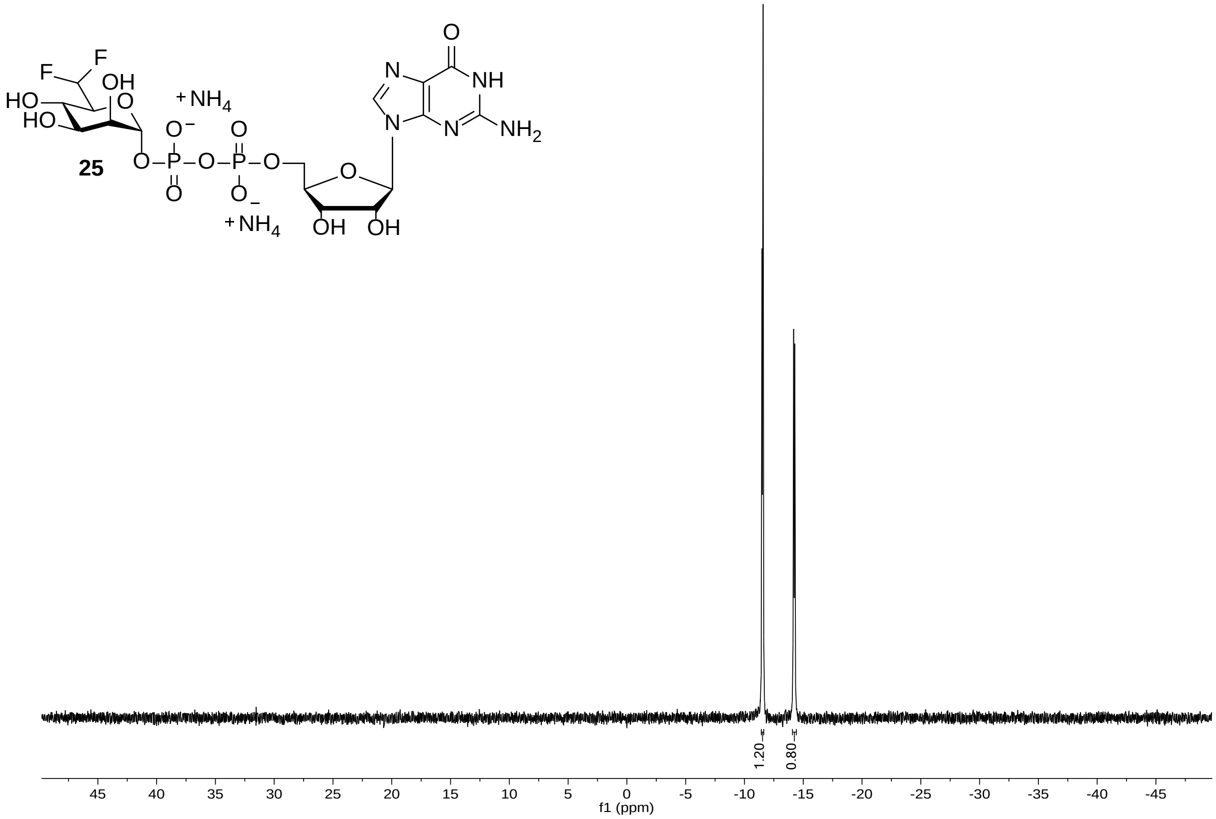
25
1.20
0.80
45 40 35 30 25 20 15 10 5 0 -5 -10 -15 -20 -25 -30 -35 -40 -45
f1 (ppm)

References

1. Pfeiffer, M.; Johansson, C.; Krojer, T.; Kavanagh, K. L.; Oppermann, U.; Nidetzky, B., A Parsimonious Mechanism of Sugar Dehydration by Human GDP-Mannose-4,6-dehydratase. *ACS Catalysis* **2019,** *9* (4), 2962-2968.
2. Yu, S.-H.; Boyce, M.; Wands, A. M.; Bond, M. R.; Bertozzi, C. R.; Kohler, J. J., Metabolic labeling enables selective photocrosslinking of O-GlcNAc-modified proteins to their binding partners. *Proceedings of the National Academy of Sciences* **2012,** *109* (13), 4834-4839.
3. Timmer, B. J. J.; Flos, M. A.; Jørgensen, L. M.; Proverbio, D.; Altun, S.; Ramström, O.; Aastrup, T.; Vincent, S. P., Spatially well-defined carbohydrate nanoplatforms: synthesis, characterization and lectin interaction study. *Chemical Communications* **2016,** *52* (83), 12326-12329.
4. Khan, S. H.; Jain, R. K.; Abbas, S. A.; Matta, K. L., Synthesis of some monodeoxyfluorinated methyl and 4-nitrophenyl α-d-mannobiosides and a related 4-nitrophenyl α-d-mannotrioside. *Carbohydrate Research* **1990,** *198* (2), 259-273.
5. Crich, D.; Vinogradova, O., Synthesis and Glycosylation of a Series of 6-Mono-, Di-, and Trifluoro S-Phenyl 2,3,4-Tri-O-benzyl-thiorhamnopyranosides. Effect of the Fluorine Substituents on Glycosylation Stereoselectivity. *Journal of the American Chemical Society* **2007,** *129* (38), 11756-11765.
6. Tosin, M.; Murphy, P. V., Synthesis of Structurally Defined Scaffolds for Bivalent Ligand Display Based on Glucuronic Acid Anilides. The Degree of Tertiary Amide Isomerism and Folding Depends on the Configuration of a Glycosyl Azide. *The Journal of Organic Chemistry* **2005,** *70* (10), 4107-4117.
7. Nakai, K.; Takagi, Y.; Tsuchiya, T., Synthesis and antitumor activity of 7-O-[2,6-dideoxy-2-fluoro-5-C-(trifluoromethyl)-α-l-talopyranosyl]-daunomycinone and -adriamycinone. *Carbohydrate Research* **1999,** *316* (1), 47-57.
8. Liras, M.; García, O.; Guarrotxena, N.; Palacios-Cuesta, M.; Quijada-Garrido, I., Versatile thiolated thermosensitive polymers synthesized by ATRP of MEO2MA and AcSEMA, a new methacrylic monomer with a protected thiol group. *Polymer Chemistry* **2013,** *4* (24), 5751-5759.
9. Li, T.; Tikad, A.; Fu, H.; Milicaj, J.; Castro, C. D.; Lacritick, M.; Pan, W.; Taylor, E. A.; Vincent, S. P., A General Strategy to Synthesize ADP-7-Azido-heptose and ADP-Azido-mannoses and Their Heptosyltransferase Binding Properties. *Organic Letters* **2021,** *23* (5), 1638-1642.
