# Supplementary material for: Fluorinated rhamnosides inhibit cellular fucosylation
Source: Nat Commun. 2021 Dec 2;12:7024. doi: 10.1038/s41467-021-27355-9 (PMC8640046; doi:10.1038/s41467-021-27355-9)
Supplement: Supplementary file 2 — Reporting Summary [file 41467_2021_27355_MOESM2_ESM.pdf]

nature portfolio

Corresponding author(s): Johan F.A. Pijnenborg; Thomas J. Boltje

Last updated by author(s): Nov 5, 2021

Reporting Summary

Nature Portfolio wishes to improve the reproducibility of the work that we publish. This form provides structure for consistency and transparency in reporting. For further information on Nature Portfolio policies, see our Editorial Policies and the Editorial Policy Checklist.

Statistics

For all statistical analyses, confirm that the following items are present in the figure legend, table legend, main text, or Methods section.

n/a	Confirmed	
☐	☒	The exact sample size (*n*) for each experimental group/condition, given as a discrete number and unit of measurement
☐	☒	A statement on whether measurements were taken from distinct samples or whether the same sample was measured repeatedly
☐	☒	The statistical test(s) used AND whether they are one- or two-sided<br>*Only common tests should be described solely by name; describe more complex techniques in the Methods section.*
☐	☒	A description of all covariates tested
☐	☒	A description of any assumptions or corrections, such as tests of normality and adjustment for multiple comparisons
☐	☒	A full description of the statistical parameters including central tendency (e.g. means) or other basic estimates (e.g. regression coefficient) AND variation (e.g. standard deviation) or associated estimates of uncertainty (e.g. confidence intervals)
☒	☐	For null hypothesis testing, the test statistic (e.g. *F*, *t*, *r*) with confidence intervals, effect sizes, degrees of freedom and *P* value noted<br>*Give P values as exact values whenever suitable.*
☒	☐	For Bayesian analysis, information on the choice of priors and Markov chain Monte Carlo settings
☒	☐	For hierarchical and complex designs, identification of the appropriate level for tests and full reporting of outcomes
☒	☐	Estimates of effect sizes (e.g. Cohen's *d*, Pearson's *r*), indicating how they were calculated

Our web collection on statistics for biologists contains articles on many of the points above.

Software and code

Policy information about availability of computer code

Data collection	FlowJo V10 (FlowJo LLC); Compass Data Analysis (Bruker) version 5.1
Data analysis	Microsoft Excel 365; Graphpad Prism 5. Python (v 3.8.5 & v 3.9.2) (Pandas (v 1.1.2 & v 1.3.2) and Seaborn (v 0.11 & v 0.11.1) libraries); MOE's 2020 QuickPrep module; MestReNova v14.1.0-24037

For manuscripts utilizing custom algorithms or software that are central to the research but not yet described in published literature, software must be made available to editors and reviewers. We strongly encourage code deposition in a community repository (e.g. GitHub). See the Nature Portfolio guidelines for submitting code & software for further information.

Data

Policy information about availability of data

All manuscripts must include a data availability statement. This statement should provide the following information, where applicable:

- Accession codes, unique identifiers, or web links for publicly available datasets
- A description of any restrictions on data availability
- For clinical datasets or third party data, please ensure that the statement adheres to our policy

The authors declare that the data supporting the findings of this study are available within the paper and its supplementary information files. All raw data files are stored on the Radboud University data management server and available upon request. For in silico experiments we obtained data from the PDB-redo database (https://pdb-redo.eu/ , PDB code 6GPJ). The GMDS digestion data was subjected to a Mascot MS/MS ion search against the SwissProt data bank (https://www.matrixscience.com/).

Field-specific reporting

Please select the one below that is the best fit for your research. If you are not sure, read the appropriate sections before making your selection.

☒ Life sciences ☐ Behavioural & social sciences ☐ Ecological, evolutionary & environmental sciences

For a reference copy of the document with all sections, see nature.com/documents/nr-reporting-summary-flat.pdf

Life sciences study design

All studies must disclose on these points even when the disclosure is negative.

Sample size	There were no sample sizes calculated. Flow cytometry were measured to contain at least 10000 gated cells which is common practice to obtain statistically relevant data.
Data exclusions	No data was excluded from analyses
Replication	All data from cellular assays was at least biological n=3, meaning they were obtained from cell lines at different passage numbers. IC50-measurement was performed in duplicate. For cellular assays and IC50, the data was averaged over all replicates, so all attempts of replication are represented in the manuscript with the exception of a single experiment for the lectin specificity assay where the control sample gave no signal. Synthetic procedures towards Fucotrim I and Fucotrim II were replicated successfully over 3 times for resynthesis of the active compounds.
Randomization	Randomization was not applicable. All samples in cell culture, after addition of different compounds, were treated equally during workup. To avoid bias of position on culture plates, orders of samples on the plate were occasionally switched. Enzymatic assay samples for IC50 were measured with LC-MS in a randomized order.
Blinding	Blinding was not relevant for this work, because no group allocation was performed/necessary

Reporting for specific materials, systems and methods

We require information from authors about some types of materials, experimental systems and methods used in many studies. Here, indicate whether each material, system or method listed is relevant to your study. If you are not sure if a list item applies to your research, read the appropriate section before selecting a response.

Materials & experimental systems

n/a	Involved in the study	
☒	☐	Antibodies
☐	☒	Eukaryotic cell lines
☒	☐	Palaeontology and archaeology
☒	☐	Animals and other organisms
☒	☐	Human research participants
☒	☐	Clinical data
☒	☐	Dual use research of concern

Methods

n/a	Involved in the study	
☒	☐	ChIP-seq
☐	☒	Flow cytometry
☒	☐	MRI-based neuroimaging

Eukaryotic cell lines

Policy information about cell lines

Cell line source(s)	THP-1 cells (TIB-202, ATCC), Jurkat cells (TIB-152, ATCC), EL-4 cells (TIB-39, ATCC) were kindly provided by the department of Radiotherapy and Oncoimmunology, Radboud University Medical Center, Nijmegen
Authentication	none of the cell lines were authenticated prior or after the experiments
Mycoplasma contamination	All cell lines were tested negative before they were first cultured in the lab. No mycoplasma tests were performed at the end of the experiments
Commonly misidentified lines (See ICLAC register)	No cell lines, listed in the ICLAC database of commonly misidentified lines were used

Flow Cytometry

Plots

Confirm that:

- [x] The axis labels state the marker and fluorochrome used (e.g. CD4-FITC).
- [x] The axis scales are clearly visible. Include numbers along axes only for bottom left plot of group (a 'group' is an analysis of identical markers).
- [x] All plots are contour plots with outliers or pseudocolor plots.
- [x] A numerical value for number of cells or percentage (with statistics) is provided.

Methodology

Sample preparation	THP-1 cells (TIB-202, ATCC) and Jurkat cells (TIB-152, ATCC) were cultured in RPMI-1640 medium containing 2 mM Glutamine and 25 mM HEPES (GibcoTM, Life Technologies), supplemented with 10% v/v heat-inactivated fetal bovine serum (FBS) (GibcoTM, Life Technologies) and 1X antibiotic-antimycotic solution (100 units/mL of penicillin, 100 µg/mL of streptomycin, and 0.25 µg/mL Fungizone) (GibcoTM, Life Technologies) and passaged every 3-4 day by seeding a fraction of 0.5-2 mln cells of the culture per 10 mL medium. EL-4 cells (TIB-39, ATCC) were cultured in DMEM medium (GibcoTM, Life Technologies), supplemented with 10% v/v heat-inactivated fetal bovine serum (FBS) (GibcoTM, Life Technologies), 1X antibiotic-antimycotic solution (100 units/mL of penicillin, 100 µg/mL of streptomycin, and 0.25 µg/mL Fungizone) (GibcoTM, Life Technologies) and 2 mM Glutamine (GibcoTM, Life Technologies) and passaged every 2-4 days by seeding a fraction of 0.5-1 mln cells of the culture per 10 mL medium. All cells were cultured at 37°C, 5% CO2 in a humidified incubator. The source of cells was always from continuous cell culture in logarithmic growth for at least 3 passages after defrosting.
Instrument	Beckman & Dickinson FACS-Calibur
Software	FlowJo V10; Microsoft Excel 365; Graphpad Prism 5
Cell population abundance	For all samples (with the exeption for samples with toxic concentrations of compounds) viable single cells accounted for at least 70%-95% of the total events.
Gating strategy	For all samples, single viable cells were gated from debris and clustered cells in the FSC/SSC plots. PE fluorescence was measured on all viable cells without further gating.

- [x] Tick this box to confirm that a figure exemplifying the gating strategy is provided in the Supplementary Information.
